# Lifetime chemical sensor arrays of organic fluorophores for bacterial fingerprinting

**DOI:** 10.1038/s41467-026-72342-7

**Published:** 2026-04-28

**Authors:** Yanzi Zhou, Charles Lochenie, Sheelagh Duncan, Jennifer Marshall, Matthieu Vermeren, David H. Dockrell, Bethany Mills, Marc Vendrell

**Affiliations:** 1 Centre for Inflammation Research, Institute for Regeneration and Repair, The University of Edinburgh, Edinburgh, UK; 2 IRR Chemistry Hub, Institute for Regeneration and Repair, The University of Edinburgh, Edinburgh, UK; 3 Baillie Gifford Pandemic Science Hub, Centre for Inflammation Research, Institute for Regeneration and Repair, The University of Edinburgh, Edinburgh, UK

**Keywords:** Chemical biology, Chemical tools, Bacteriology

## Abstract

The rise of multi-drug resistant bacteria constitutes a global challenge with direct impact on millions of patients worldwide. The identification of bacterial species is essential to avoid unnecessary use of antibiotics and to minimize the emergence of additional resistance; however, there are few chemical strategies to identify resistant species in a rapid and unbiased manner. Cross-reactive arrays combine multiple sensor components to produce distinctive fingerprints for similar targets; herein, we present a cross-reactive sensing array built with long lifetime organic fluorophores. To the best of our knowledge, we synthesize the largest collection to date of bioconjugatable triangulenium fluorophores by including heteroatom and side-chain diversification for spectral and lifetime diversity, as well as water-soluble and orthogonal moieties for peptide tagging and compatibility with biological samples. After optimization, we evolve a four-fluorophore sensor array that correctly assigns all seven ESKAPEE bacterial species by combining their optical signatures. Lifetime chemical sensor arrays will open avenues for concentration-independent, reliable and sensitive optical detection without the need for prior knowledge of molecular targets.

## Abstract (web summary)

Infections caused by multi-drug resistant bacteria present a global health challenge, so the identification of bacterial species is essential to avoid the unnecessary use of antibiotics and to minimize the emergence of additional resistance. Here, the authors present a lifetime cross-reactive sensor array that correctly assigned all seven ESKAPEE bacterial species by combining their optical signatures.

## Introduction

Infectious diseases represent one of the global challenges of the twenty-first century, particularly due to the rise of antimicrobial-resistant pathogens^1^. Recent studies report that the number of deaths caused by multi-drug-resistant bacteria (i.e., gram-positive and gram-negative ESKAPEE bacteria, which are a priority to the WHO and a threat to human health) exceeds several million worldwide every year^2^. Effective strategies to maximize therapeutic efficacy while minimizing additional resistance involve the identification of bacterial species to choose suitable antimicrobials and avoid the use of unnecessary antibiotics^3^; however, the identification of resistant bacteria is hampered by (1) the paucity of strategies that do not rely on genomic amplification (e.g., polymerase chain reaction (PCR)-based sensors), and (2) the ever-growing diversification of bacterial species with concomitant lack of acquired resistance biomarkers^4,5^. To this end, the design of sensing approaches that can identify resistant bacteria without prior knowledge of their molecular targets could represent a promising avenue for the microbiological analysis of ESKAPEE bacterial species.

The majority of sensing strategies for bacterial detection -including optical and electrochemical sensors- rely on traditional lock-and-key recognition^6^. These systems require specific targeting biomolecules, which cannot always resolve subtle differences between similar cell types^7^. On the other hand, cross-reactive arrays exploit the combined discriminatory power of multiple components to produce unique fingerprints for closely-related targets. Building on the seminal work by the groups of Walt and Suslick in optical sensing arrays^8,9^, cross-reactive sensors using metal nanoparticles, organic semiconductor films, biopolymers, and optical reporters have been described^10–12^. For instance, Reuveni, Shabat, Fridman, and co-workers recently developed a chemiluminescence array of phenoxydioxetanes to classify bacterial cells on the basis of their enzymatic activity^13^; however, optical arrays based on intensity measurements are dependent on the concentrations of both individual sensors and targets (e.g., bacterial cell density), which hinders their reproducibility in complex clinical samples.

Fluorescence lifetimes report the average time that fluorophores spend in their excited state before emitting a photon and returning to the ground state^14^. Because the lifetimes of organic fluorophores relate to their surrounding microenvironments, they are excellent reporters of cellular features, even when those are undefined at the molecular level^15,16^. Furthermore, unlike intensity readouts, fluorescence lifetime measurements are independent of instrumentation and fluorophore concentration, which facilitates their application in cross-reactive arrays. Our group and others have demonstrated that the structural fine-tuning of organic fluorophores enables their use in multi-color fluorescence imaging^17,18^; however, a major limitation in the design of fluorescence lifetime sensor arrays is the lack of chemical toolboxes with (1) diverse and long lifetimes above autofluorescence levels (>5 ns) and (2) biocompatible and water-soluble groups for further conjugation and biological sampling.

Here, we present the chemical design and characterization of triangulenium (TA) fluorophores alongside their application in fluorescence lifetime chemical sensor arrays. To the best of our knowledge, this work represents one of the first examples of a lifetime sensor array built with organic fluorophores. Our TA fluorophore collection exhibited broad chemical diversity (e.g., positive and negative charges, reactive groups for bioconjugation) and suitable optical properties (e.g., long fluorescence lifetimes, signal reproducibility, photostability) to construct cross-reactive arrays for sensing applications. As an exemplar proof-of-concept, we demonstrated that the combination and optimization of TA fluorophores -via modification with bacteria-targeting peptides- resulted in arrays for identification of all seven ESKAPEE bacterial species (*Enterococcus faecium, Staphylococcus aureus, Klebsiella pneumoniae, Acinetobacter baumannii, Pseudomonas aeruginosa, Enterobacter species*, and *Escherichia coli*). This strategy holds potential to accelerate the chemical design of optical sensing platforms without requiring molecular knowledge on target composition and with potential applications in many different biological systems.

## Results

### Design, synthesis, and characterization of a library of bioconjugatable TA fluorophores for FLIM applications

Triangulenium structures have been reported as a robust chemical scaffold to synthesize stable fluorophores with long lifetimes in the 5–30 ns range^19–26^. Several TA fluorophores have been described for fluorescence lifetime imaging microscopy (FLIM) of mammalian cells, G4 quadruplex DNA, and intracellular pH^27–30^; however, their application in other biological systems has been hindered by the poor solubility in aqueous media and limited strategies for bioconjugation^31,32^. Another interesting feature of TA fluorophores is their suitability for optochemical tuning via substitution of the bridging atoms in their core, as reported for other fluorophores^33–38^. Given all these properties, we envisioned that the chemical modification of TA fluorophores with variable bridging atoms as well as side chains of different lengths and electrostatic charges would render collections of TA fluorophores with diverse spectra and lifetimes as well as improved aqueous solubility.

**Fig. 1:**
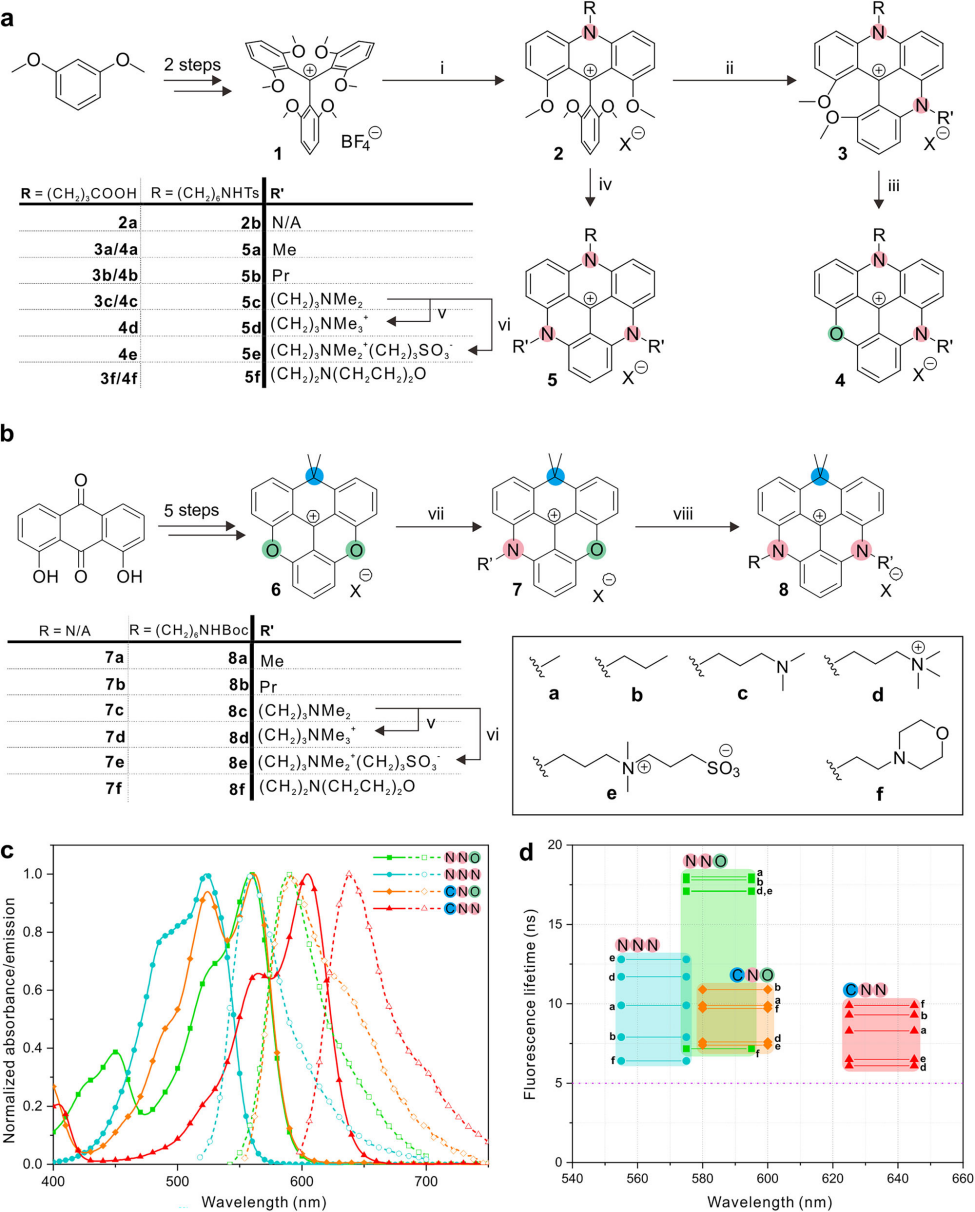
Synthesis and optical characterization of TA fluorophores. **a** Synthesis of asymmetric N-bridged TA fluorophores (**4**) and symmetric N-bridged TA fluorophores (**5**). Reaction conditions: (i) RNH_2_, MeCN, r.t., 18 h; (ii) R’NH_2_, MeCN, 66 °C, 18 h; (iii) Py·HCl, 220 °C, 15 min; (iv) R’NH_2_, NMP, 210 °C, 18 h; (v) MeI, Na_2_CO_3_, DMF, r.t., 3 h; (vi) sultone, DIPEA, DMF, r.t., 72 h. **b** Synthesis of asymmetric C-bridged TA fluorophores (**7**) and symmetric C-bridged TA fluorophores (**8**). Reaction conditions: (vii) R’NH_2_, NMP, 90 °C, 18 h; (viii) RNH_2_, NMP, 90 °C, 18 h. **c** Absorbance (solid lines) and emission (dashed lines) spectra for representative TA fluorophores from all four families (5 μM). **d** Plot of fluorescence lifetimes vs absorbance maxima (left symbol) and emission maxima (right symbol) for all TA fluorophores (5 μM). X^−^: PF_6_^−^, TFA^−^ (trifluoroacetate) or FA^−^ (formate).

We designed the synthesis of two families of *N*-bridged asymmetric (**4**, DAOTA^39^, Fig. 1a) and N-bridged symmetric TA fluorophores (**5**, TATA^40^, Fig. 1a) starting from the triarylcarbenium **1**, which was obtained in multigram scale and in two steps following reported procedures^40^. First, we prepared two precursors incorporating either carboxylic acid or amine conjugatable groups (**2a** with γ-aminobutyric acid, **2b** with a tosyl-protected hexamethylenediamine). The former was reacted with primary amines of different length under mild heating (~65 °C) to afford the ring-open compounds (**3a,** **3b,** **3c**, and **3f**; Fig. 1a). The corresponding asymmetric TA fluorophores (**4a,** **4b,** **4c**, and **4f**; Fig. 1a) were isolated via ring-closure in neat melted pyridinium chloride and purification by preparative HPLC. Furthermore, the TA fluorophores **4d** and **4e** bearing positively and negatively charged moieties (i.e., trimethylammonium and sulfonate salts, respectively) were prepared by alkylation of compound **4c** because the corresponding amines degraded during ring closure at temperatures above 200 °C (Fig. 1a). In parallel, the synthesis of symmetric TA fluorophores was performed only with the precursor **2b** as we observed decomposition of the carboxylic acid in compound **2a** at high temperatures (>200 °C) that were necessary to form the triple-aza bridge. The tosyl protecting group proved superior to *tert*-butyloxycarbonyl under these experimental conditions, and we isolated the TA fluorophores (**5a, 5b, 5c**, and **5f**; Fig. 1a) in 20-30% recovery yields, with the exception of compound **5f**, where lower yields were obtained due to the instability of *N*-(aminoethyl)morpholine at high temperatures. Analogous to the synthesis of compounds **4d** and **4e**, we prepared the corresponding charged TA fluorophores **5d** and **5e** by alkylation of compound **5c** (Fig. 1a).

We also prepared two families of *C*-bridged TA fluorophores (**7**, CAOTA^26^; **8**, CDATA^41^, Fig. 1b) from the common dioxatriangulenium salt precursor **6**, which was obtained in gram scale following reported methods^26^. In this case, the sequential introduction of primary amines via aromatic nucleophilic substitution required lower temperatures (~90 °C) than for compounds **4** and **5** (Fig. 1b), and it was compatible with a Boc-protected hexamethylenediamine spacer (Fig. 1b). All *C*-bridged TA fluorophores (**7a,** **7b,** **7c,** **7f,** **8a,** **8b,** **8c**, and **8f**, Fig. 1b) were isolated by preparative HPLC in reasonable yields reaching 40%, with the above-mentioned exception for morpholine-containing fluorophores (**7f** and **8f**). To overcome this limitation in scale-up efforts, we developed a more efficient synthetic strategy where morpholine-containing fluorophores were prepared from the chemically stable ethanolamine precursor (Supplementary Note 1). The charged TA fluorophores (**7d,** **7e,** **8d,** **8e**; Fig. 1b) were respectively prepared from compounds **7c** and **8c** as above described for *N*-bridged compounds. The final 20 TA fluorophores were obtained in the 5–10 mg range and in purities >95% (full synthetic and characterization details included in the Supplementary Information). Notably, all fluorophores were soluble in aqueous media, including a maximum of 1% DMSO, and TA fluorophores with charged chains (**4d**/**e,** **5d**/**e,** **7d**/**e**, and **8 d**/**e**) proved to be fully water soluble at concentrations up to 500 μM.

Next, we determined the steady-state fluorescence properties of all TA fluorophores. The four families of TA fluorophores could be broadly classified into three separate spectral regions depending on the combination of bridging atoms (Fig. 1c and Supplementary Fig. 1). Specifically, the TA family **5a**–**f** with three N bridging atoms (TATA) displayed the shortest absorbance and emission maxima wavelengths (e.g., 520/560 nm) whereas the TA families **4a**–**f** (with two N atoms and one O atom, DAOTA) and **7a**–**f** (with one C atom, one N atom and one O atom, CAOTA) showed red-shifted absorbance and emission spectra (e.g., 560/590 nm). Finally, the TA fluorophores **8a**–**f** (with one C atom and two N atoms, CDATA) exhibited the longest bathochromic shifts (e.g., 600/635 nm). Notably, different substitution patterns did not affect the absorbance or emission spectra, which indicates that the bridging atoms are the main structural feature to define excitation and emission wavelengths (Table 1 and Supplementary Fig. 1).

**Table 1:** Summary of photophysical properties of TA fluorophores

TA fluorophores	λ_abs_ (nm)	λ_em_ (nm)	τ (ns)	Φ_PL_	Brightness (M^−1^ cm^−1^)
**4a**	554	588	20.9	0.49	3518
**4b**	557	588	20.9	0.54	3904
**4d**	554	588	20.5	0.32	1491
**4e**	554	588	19.3	0.27	1604
**4f**	557	588	7.2	0.08	5529
**5a**	521	560	10.3	0.30	3564
**5b**	523	560	8.7	0.47	7708
**5d**	522	564	12.1	0.25	3878
**5e**	529	578	14.2	0.17	2030
**5f**	525	568	7.5	0.13	2422
**7a**	560	592	9.9	0.22	1505
**7b**	562	592	10.9	0.26	1677
**7d**	560	588	7.6	0.20	1528
**7e**	560	590	7.4	0.18	1292
**7f**	562	594	9.0	0.03	223
**8a**	603	638	8.3	0.16	1462
**8b**	604	638	9.3	0.19	2510
**8d**	599	634	6.1	0.11	1222
**8e**	599	634	6.5	0.11	965
**8f**	605	644	9.4	0.04	361

Values determined in acetonitrile or water, depending on solubility. Φ_PL_: photoluminescence quantum yields; τ: fluorescence lifetimes.

We also investigated the time-resolved spectroscopic properties of the entire library. All TA fluorophores showed long excited-state lifetimes between 6 and 20 ns in water or acetonitrile (Fig. 1d and Supplementary Figs. 2–5). These values are substantially longer than those of commercial dyes with similar excitation/emission wavelengths (e.g., fluorescein (490/520 nm; τ: 4.2 ns), BODIPY-FL (500/530 nm; τ: 5.2 ns), rhodamine B (546/567 nm; τ: 1.7 ns), cyanine 3 (550/570 nm; τ: 0.1 ns))^42,43^. Among the different families, we found longer lifetimes and higher quantum yields for families **4** and **5** when compared to **7** and **8**, likely due to the latter containing a C bridge atom in their cores. Exceptionally, the morpholine-substituted derivatives (**4f** and **5f**) exhibited much lower lifetimes than the rest of the series (**4f**: 7.2 ns; **5f**: 7.5 ns). These results are likely due to the photoinduced electron transfer (PeT) quenching effect of the morpholine moiety, as reported for other fluorophores^44,45^. Finally, we calculated both radiative decay rates (k_r_) and non-radiative decay rates (k_nr_) (Supplementary Fig. 6). This analysis confirmed that TA fluorophores from families **7** and **8** exhibit higher k_nr_ rates than their counterparts in families **4** and **5**, in agreement with the shorter lifetimes detected for the former. In addition, families **4** and **5** showed slight variations in k_nr_ rates whereas k_r_ decreased with an increase of the length of the side chains. On the contrary, families **7** and **8** showed large variations in k_nr_ rates, while k_r_ rates remained relatively unaffected by chain length. Altogether, these results demonstrate that chemical fine-tuning of the triangulenium scaffold can produce TA fluorophores with a wide range of fluorescence lifetimes to build cross-reactive sensor arrays.

### An untargeted lifetime sensor array for ESKAPEE bacterial species

We started the design of lifetime sensor arrays by combining six TA fluorophores with different core bridging atoms and side chains. The first selection of TA fluorophores included members from all four families (**4,** **5,** **7** and **8**) with different excitation and emission profiles, and with reasonable quantum yields (i.e., not below 10%) to enable relatively fast fluorescence lifetime measurements. We also included TA fluorophores with charged moieties (e.g., positive charges, zwitterionic side chains) to diversify cellular recognition and uptake. On the basis of these considerations, we built a first cross-reactive array combining the TA fluorophores **4b,** **5b,** **7b,** **8b,** **8d**, and **8e**, and evaluated their capacity for the individual identification of all seven ESKAPEE bacterial species using fluorescence lifetime microscopy. Given that triangulenium structures display reasonable affinity for polynucleotides^27,41,46^, we envisaged that their binding to DNA would alter their fluorescence lifetimes and provide unique signatures of bacterial intracellular environments.

**Fig. 2:**
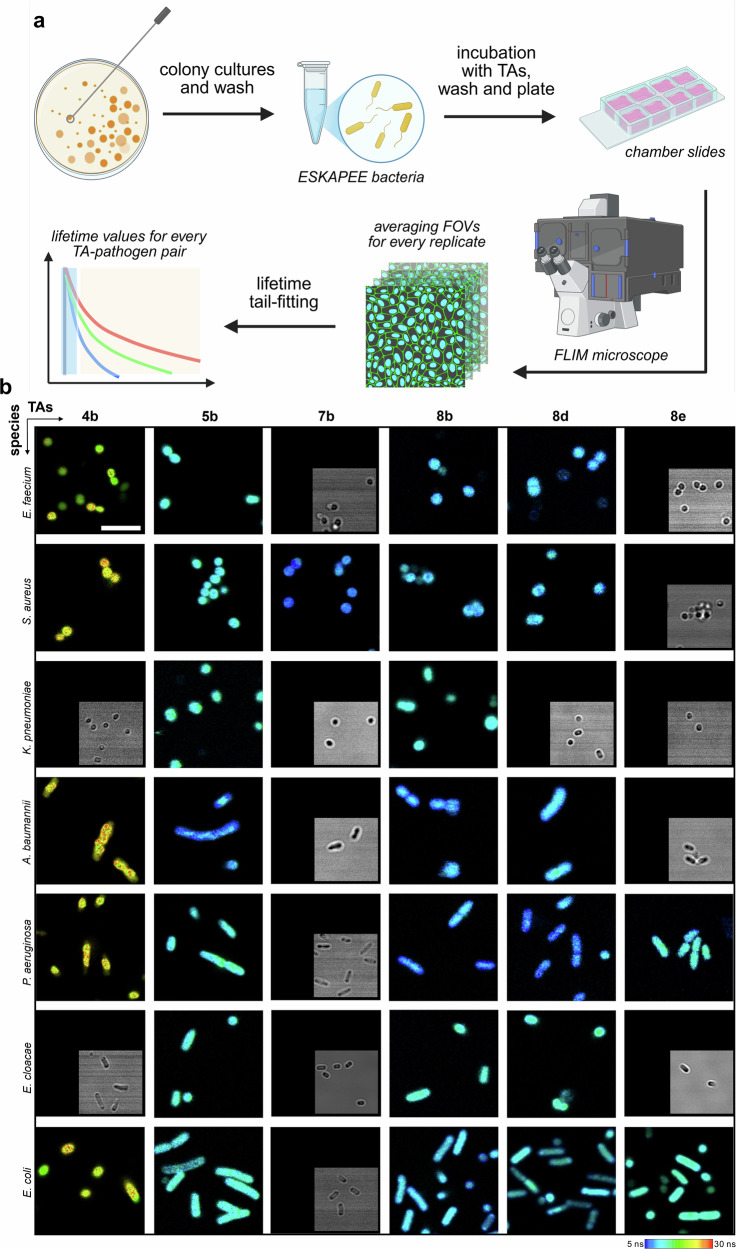
TA fluorophores show diverse uptake and intracellular fluorescence lifetimes in ESKAPEE bacteria. **a** Workflow of bacterial cell imaging using a TA-based lifetime sensor array. **b** Representative FLIM microscopy images (from 10 independent images of at least 2 independent biological replicates) of planktonic cultures of ESKAPEE bacteria after incubation with TA fluorophores (**4b,** **5b,** **7b,** **8b**: all at 5 µM; **8d** and **8e**: all at 10 µM). Bacteria were cultured until mid-log phase, incubated with TA fluorophores for 30 min at 37 °C and washed prior to imaging. Images were recorded at different excitation wavelengths (530 nm (**4b**), 488 nm (**5b**), 523 nm (**7b**) and 580 nm (**8b,** **8d** and **8e**); emission ranges: 550–700 nm (**4b**), 508-650 nm (**5b**), 543–700 nm (**7b**) and 600–800 nm (**8b,** **8d** and **8e**), with repetition rates of 2.5 MHz, 5 MHz, 5 MHz and 5 MHz, respectively. Pseudocolored images presented with a linear LUT (5–30 ns). Inserts: brightfield microscopy images included to show bacterial cells within the field-of-view when no fluorescence labeling was observed. Scale bar: 5 µm.

ESKAPEE bacterial species are considered a threat to human health because of their acquisition of resistance genes against a large range of drugs, including last-resort antimicrobials^47^. They include two gram-positive bacteria (i.e., *E. faecium* and *S. aureus*) and five gram-negative bacteria (i.e., *K. pneumoniae, A. baumannii*, *P. aeruginosa, Enterobacter spp.*(e.g., *E. cloacae))* and *E. coli*) and represent a biologically-diverse collection to test the discriminatory capabilities of cross-reactive arrays (Fig. 2a)^48^. We cultured all ESKAPEE bacteria individually under the same experimental conditions until they reached an exponential growth phase; then, we incubated them for 30 min with the TA fluorophores and imaged the cells under FLIM. For every pathogen-TA combination, we acquired a minimum of 5 fields of view for each biological repeat (representative images for all combinations shown in Fig. 2b; fluorescence intensity and FLIM images in Supplementary Figs. 7 and 8). Next, we determined their lifetimes using the tail-fit model, where the short-lived contributions from autofluorescence signals were clearly distinguishable from the TA fluorophores. Experimental procedures for bacterial cell culture, image analysis, and lifetime data treatments are detailed in the Supporting Information and in the extended Supplementary Note 2.

**Fig. 3:**
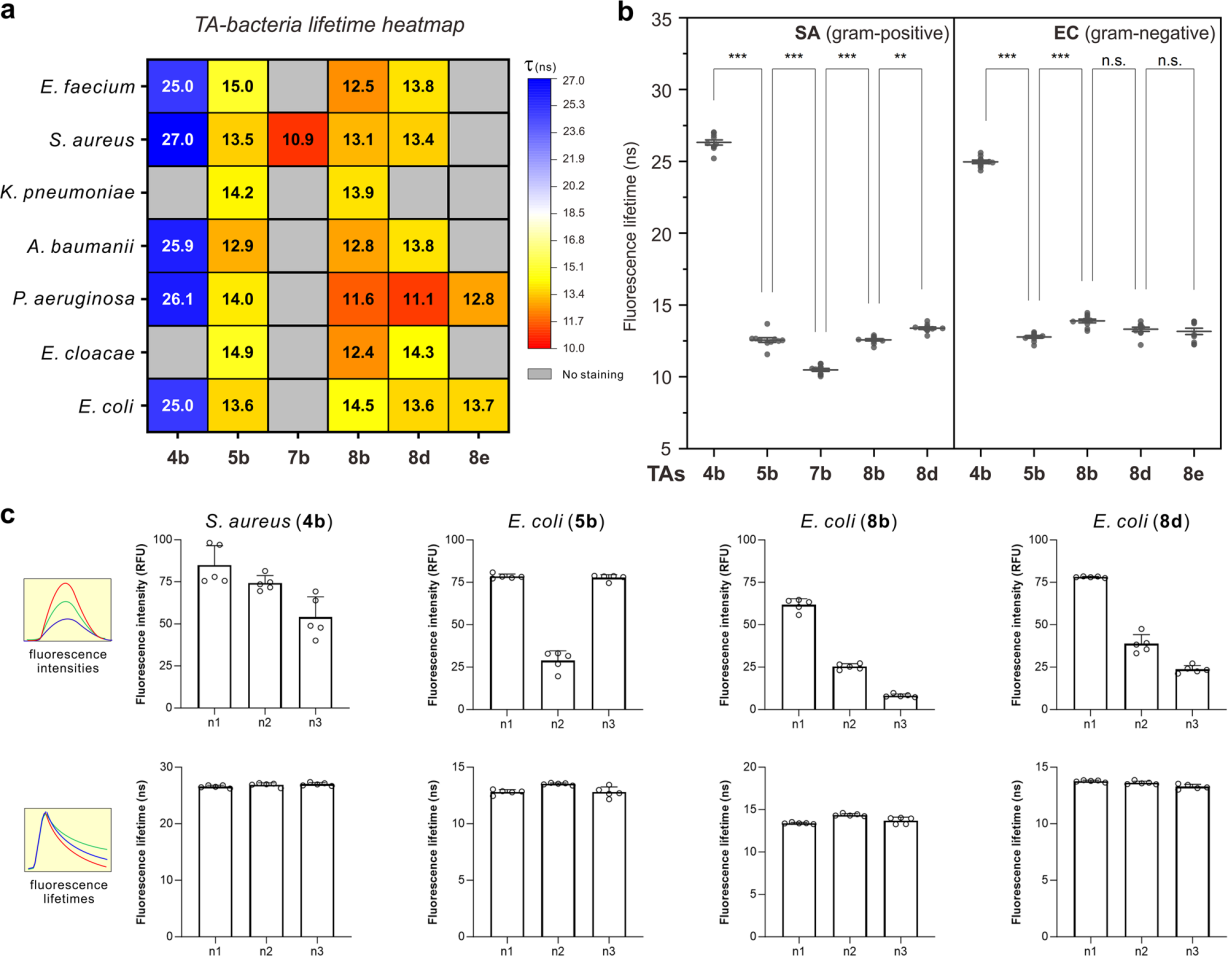
Cross-reactive arrays of untargeted TA fluorophores can distinguish most—but not all—ESKAPEE bacterial species. **a** Pseudocolored heatmap of lifetimes of the TA fluorophore array in ESKAPEE bacterial species. Gray squares indicate absence of cell labeling. **b** Fluorescence lifetimes -extracted from FLIM images- for representative gram-positive (*S. aureus*) and gram-negative (*E. coli*) bacteria after labeling with TA fluorophores. Values as means ± SEM obtained from 10 measurements across independent biological replicates. Statistical analysis performed by ANOVA (n.s. for *p* > 0.05, ** for *p* < 0.01, *** for *p* < 0.001). *P* values (*S. aureus*): 8.9 × 10^−22^ (**4b** vs **5b**), 2.3 × 10^−9^ (**5b** vs **7b**), 3.8 × 10^−12^ (**7b** vs **8b**), 1.8 × 10^−3^ (**8b** vs **8d**); (*E. coli*): 1.8 × 10^−6^ (**4b** vs **5b**), 1.6 × 10^−24^ (**5b** vs **8b**), 5.2 × 10^−2^ (**8b** vs **8d**), 0.08 (**8d** vs **8e**). Full statistical analysis across all bacterial species is presented in Supplementary Figs. 10 and 11. **c** Relative fluorescence intensity units (top panels) and fluorescence lifetimes (bottom panels) for different TA fluorophores (**4b** and **5b**: 5 μM; **8b** and **8d**: 10 μM) in gram-positive and gram-negative bacteria across three independent biological replicates (*n*1–*n*3, values as means ± SEM). Each datapoint represents the mean fluorescence intensity (top panels) or the median fluorescence lifetime (bottom panels) for individual fields of view.

Notably, the readouts of the array showed diversity in both bacterial cell uptake and intracellular fluorescence lifetimes. Some TA fluorophores (i.e., fluorophores **5b,** **8b** and **8d**) exhibited long lifetimes across all ESKAPEE bacterial species, whereas other fluorophores were only detectable by FLIM in some species (e.g., fluorophore **7b** showed only bright signals in gram-positive *S. aureus*; fluorophore **8e** only labeled gram-negative *E. coli* and *P. aeruginosa*) (Fig. 3a). With the exception of an unusually broad distribution for compound **5b**, TA fluorophores presented normal distributions of fluorescence lifetimes that were on average longer in bacterial cells than in buffered solution (Supplementary Figs. 9 and 10). Among the TA fluorophores **8b,** **8d**, and **8e**, we qualitatively observed that the fluorophore **8e**, which contained hydrophilic zwitterionic side chains (net charge of +1), showed poorer uptake than the fluorophore **8b**, which included propyl side chains (net charge of +1). Moreover, the fluorophore **8d**, which contained positively-charged side chains (net charge of +3), was able to label most bacterial species. These observations suggest that both lipophilicity and positive charges may enhance the permeability of TA fluorophores to bacterial cell envelopes. Furthermore, the combination of fluorescence lifetimes in a single heatmap plot featured different fingerprints for different bacteria (i.e., gram-positive and gram-negative bacteria, Fig. 3a). Our results also highlighted variable degrees of statistical significance (Fig. 3b and Supplementary Fig. 11) and an outstanding dynamic range for the TA fluorophores (i.e., from 10 to 30 ns), which is beyond other fluorophores employed in FLIM studies^49–51^. We confirmed that the behavior of TA fluorophores was due to interactions with bacterial DNA by comparing their lifetimes before and after incubation with different DNA sources. We observed that the fluorescence lifetimes of TA-labeled bacteria were similar to those of DNA-bound TA fluorophores (Supplementary Fig. 12), as well as independent of pH and viscosity (Supplementary Figs. 13 and 14). Finally, we analyzed the experimental deviation of both fluorescence intensity and lifetime readouts of TA-labeled bacteria across independent biological replicates. As shown in Fig. 3c, we found much higher consistency across biological replicates for lifetime values over fluorescence intensity values. These results corroborate the chemical robustness of TA fluorophores and their suitability for building lifetime cross-reactive arrays.

Finally, we performed principal component analysis (PCA)^52,53^ to classify all seven ESKAPEE bacterial species. Using the combined readouts of the six TA fluorophores (**4b,** **5b,** **7b,** **8b,** **8d** and **8e**), we could distinguish most bacterial species by three-component analysis, where the largest variance contributions arose TA fluorophores **4b,** **8d**, and **8e** and the contributions from fluorophores **5b** and **8b** were negligible because of their similar lifetimes in different bacterial species (Supplementary Fig. 15). Projected results along the PC1-PC2 axis with 95% confidence intervals showed that most ESKAPEE bacterial species were distinguishable, with only some exceptions (i.e., *E. faecium* from *A. baumannii* and *P. aeruginosa* from *E. coli*, Supplementary Fig. 15). Altogether, these results confirmed that (1) the combined fluorescence lifetimes of untargeted TA fluorophores produced differential fingerprints for most ESKAPEE bacterial species and (2) enhanced bacterial species discrimination required expanding the TA fluorophore toolbox towards other cellular microenvironments.

### TA-peptide conjugates magnify the differences between ESKAPEE bacterial species

**Fig. 4:**
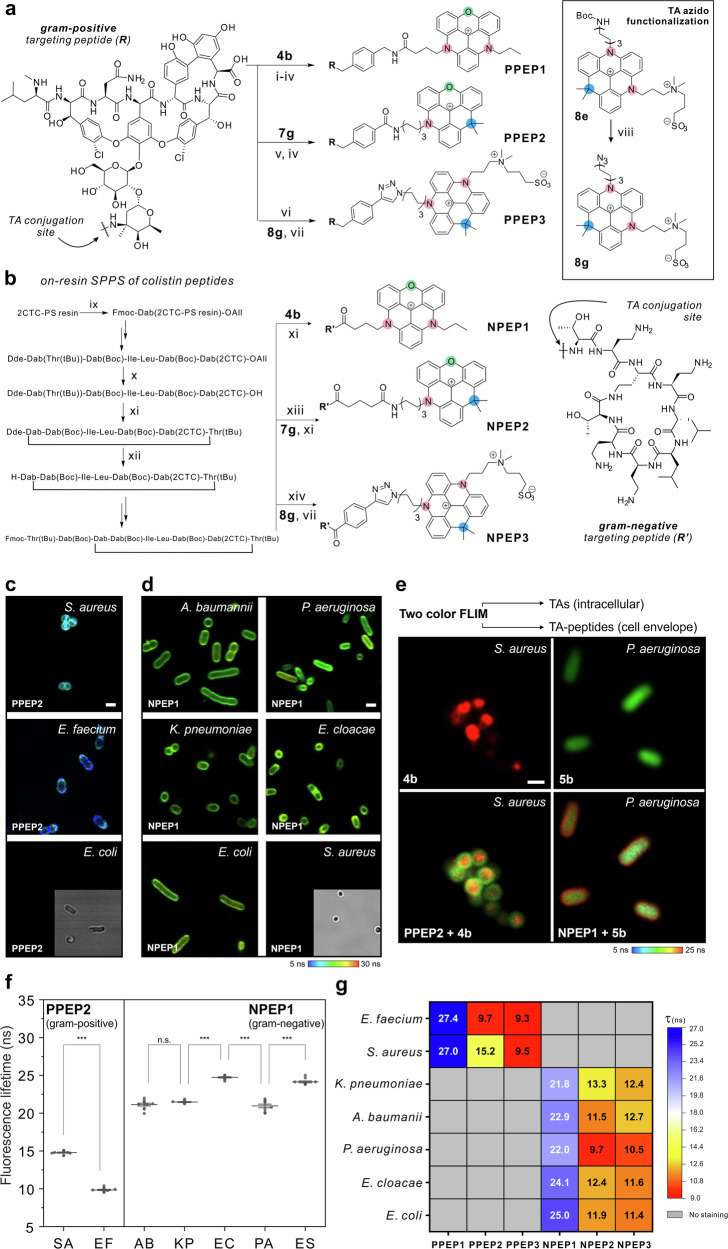
Synthesis and characterization and FLIM images of TA-peptides targeting gram-positive and gram-negative bacteria. **a**, **b** Synthetic scheme and chemical structures of TA-vancomycin derivatives (**PPEP1-PPEP3**) and TA-colistin derivatives (**NPEP1-NPEP3**). Conditions: (i) (4-(TBDMS)oxymethyl)phenylmethanamine, DCC, DMAP, MeCN, r.t., 48 h; (ii) 1 M TBAF in THF, r.t., 16 h; (iii) DMP, DCM, r.t., 4 h; (iv) vancomycin HCl, DIPEA, DMF, 55 °C at 4 h then NaBH_3_CN, TFA, MeOH, r.t., 2 h; (v) 4-(hydroxymethyl)benzaldehyde, DCC, DMAP, THF, r.t., 1 h; (vi) vancomycin HCl, 4-ethynylbenzaldehyde, DIPEA, DMF, 55 °C at 4 h then NaBH_3_CN, TFA, MeOH, r.t., 2 h; (vii) CuSO_4_, sodium ascorbate, L-histidine, H_2_O, r.t., 30 min; (viii) TFA in DCM, r.t., 1 h, then imidazole-1-sulfonyl azide hydrochloride, K_2_CO_3_, CuSO_4_, MeOH, r.t., 2 h; (ix) Fmoc-Dab(NH_2_)-OAll, Et_3_N, DCM, r.t., 24 h, (x) Pd(PPh_3_)_4_, PhSiH_3_, DCM, 2 h, r.t.; (xi) DIC, Oxyma, DMF, r.t., 1 h; (xii) NH_2_OH·HCl, imidazole, DMF, r.t., 3 h; (xiii) glutaric anhydride, pyridine, DMF, r.t., 1 h; (xiv) 4-ethynylbenzaldehyde, DIC, Oxyma, DMF, r.t., 1 h. **c**, **d** Representative FLIM microscopy images (from 10 independent images of at least 2 independent biological replicates) of planktonic cultures of ESKAPEE bacteria after incubation with TA-peptides (both **PPEP2** and **NPEP1** at 2.5 µM). Bacteria were cultured until mid-log phase growth, incubated with TA-peptides for 15 min at 37 °C, and washed prior to imaging. Images were recorded at different excitation wavelengths (530 nm (**PPEP1** and **NPEP1**), 523 nm (**PPEP2** and **NPEP2**) and 580 nm (**PPEP3** and **NPEP3**); emission ranges: 550–700 nm (**PPEP1** and **NPEP1**), 543–700 nm (**PPEP2** and **NPEP2**) and 600–800 nm (**PPEP3** and **NPEP3**), with repetition rates of 2.5 MHz, 5 MHz, and 5 MHz, respectively. Pseudocolored images presented with a linear LUT (5–30 ns). Inserts: brightfield microscopy images included to show bacteria within the field-of-view when no fluorescence staining was detected. Scale bar: 5 µm. **e** Representative FLIM microscopy images (from 3 independent experiments) of *S. aureus* and *P. aeruginosa* after incubation with both TA fluorophore and TA-peptides (**4b**: 5 µM, **PPEP2**: 2.5 µM, **5b**: 5 µM, **NPEP1**: 2.5 µM). Bacteria were cultured until mid-log phase growth, incubated with TA fluorophores for 30 min and TA-peptides for 15 min at 37 °C, and washed prior to imaging. Images were recorded at 530 nm excitation wavelengths; emission range: 550–700 nm, and repetition rate of 2.5 MHz. Pseudocolored images presented with a linear LUT (5–25 ns). Scale bar: 5 µm. **f** Fluorescence lifetimes -extracted from FLIM images- for ESKAPEE bacteria labeled with **PPEP2** or **NPEP1**. Values as means ± SEM obtained from 10 measurements across independent biological replicates. Statistical analysis performed by ANOVA (n.s. for *p* > 0.05, *** for *p* < 0.001). *P* values: 2.1 × 10^−19^ (SA vs EF), 0.109 (AB vs KP), 1.2 × 10^−16^ (KP vs EC), 1.7 × 10^−11^ (EC vs PA), 7.1 × 10^−10^ (PA vs ES). Full statistical analysis across all bacterial species is presented in Supplementary Fig. 20. **g** Pseudocolored heatmap of median lifetimes recorded for all ESKAPEE bacterial species with TA-peptides. Gray squares indicate the absence of cell labeling.

In order to improve the discriminatory power of cross-reactive TA sensor arrays and leverage the bioconjugatable properties of our TA fluorophores, we prepared TA-peptides that could locate within cellular envelopes and therefore complement the intracellular readouts of DNA-binding TA fluorophores. For this purpose, we focused on two cyclic antimicrobial peptides (i.e., vancomycin and colistin) that can interact with either peptidoglycan chains of gram-positive bacteria and lipids (e.g., lipid A) on the outer cell membrane of gram-negative bacteria, respectively. Both vancomycin and colistin were coupled to three different TA fluorophores (i.e., fluorophores **4b,** **7g**, and **8g**—an azido-containing derivative of **8e**—Fig. 4a). For vancomycin derivatives (**PPEP1**-**3**, Fig. 4a), we derivatized its primary amine via reductive amination using amine-containing or carboxylic acid-containing benzaldehyde linkers. This approach enabled direct coupling to carboxylic acid-functionalized TA fluorophore (**4b**) and amine-functionalized TA fluorophore (**7g**); however, it was incompatible with compound **8e** due to reduction of the sulfonate groups under reductive conditions. We circumvented this limitation with an alkyne-modified benzaldehyde linker that was readily conjugated to the azide-functionalized **8g** (prepared in two steps from **8e**, Fig. 4a) via copper-catalyzed alkyne-azide cycloaddition (CuAAC).

For colistin derivatives (**NPEP1-3**), we designed an on-resin synthetic strategy using conventional methods in solid-phase peptide synthesis (SPPS)^54–57^, with TA fluorophores being coupled to the N-terminal end of the cyclic peptide (Fig. 4b). This approach afforded reasonable crude mixtures (e.g., HPLC-MS purities >90%) and good recovery yields topping 50% (full synthetic and characterization details in Supporting Information). This modular synthesis could be in principle applied to other cyclic antimicrobial peptides (e.g., polymyxins) to accelerate the discovery of drugs for gram-negative bacteria. Like vancomycin conjugates, we coupled TA fluorophores to the N-terminal end using direct amide formation (**4b** to form **NPEP1**), amide formation via a short succinic acid spacer (**7g** to form **NPEP2**) or CuAAC between an alkyne-containing benzoic acid spacer and compound **8g** to form **NPEP3**. All six TA-peptides (**PPEP1-3** and **NPEP1-3**) were isolated by preparative HPLC with purities >95% in 5–30 mg amounts (characterization details in Supporting Information). We determined the photophysical properties for all TA-peptides, which showed similar absorbance and emission spectra, fluorescence lifetimes, and quantum yields than their corresponding TA fluorophores (Supplementary Fig. 16 and Supplementary Table 1).

Next, we used the same experimental procedure to acquire FLIM images of all ESKAPEE bacterial species after incubation with the six TA-peptides (**PPEP1-3,** **NPEP1-3**). Vancomycin derivatives (**PPEP1-3**) only stained gram-positive bacteria, and colistin derivatives (**NPEP1-3**) only labeled gram-negative bacteria (Fig. 4c and Supplementary Figs. 17 and 18)^58–61^. Notably, peptide conjugation could improve the cell labeling capabilities of some TA fluorophores. For instance, the TA fluorophore **7b** showed fluorescence emission only in *S. aureus* but not in other bacteria, while the **7b**-peptide conjugates (i.e., **PPEP2** and **NPEP2**) showed bright signals across all gram-positive bacteria and gram-negative bacteria. We also confirmed the compartmentalization of TA fluorophores (intracellular) and TA-peptides (cell envelope) by co-incubation of TA and TA-peptide pairs and FLIM under a single excitation channel. As shown in Fig. 4e, we acquired two-color FLIM images of *S. aureus* with **PPEP2** (~15 ns) and its corresponding TA fluorophore **4b** (~27 ns), and of *P. aeruginosa* with **NPEP1** (~22 ns) and its corresponding TA fluorophore **5b** (~4 ns). These results suggested that the combined readouts of TA fluorophores and TA-peptides could enhance the discrimination of bacterial species, given the large differences in lifetimes and cell localization.

Finally, we analyzed the fluorescence lifetime signatures of the TA-peptides in all ESKAPEE bacterial species. TA-peptides (**PPEP1-3** and **NPEP1-3**) presented normal distributions of fluorescence lifetimes in bacterial cell envelopes (Fig. 4f and Supplementary Fig. 19). Analogous to DNA-binding TA fluorophores, the longer lifetimes of TA-peptides in bacterial cells are likely due to the binding of vancomycin moieties to peptidoglycans in gram-positive bacteria (for **PPEP1-3**) or to the binding of colistin moieties to lipopolysaccharide molecules in gram-negative bacteria (for **NPEP1-3**)^62,63^. Notably, some TA-peptides showed different lifetimes across strains. For instance, **PPEP2** exhibited the best discrimination between gram-positive bacteria, and **NPEP1** displayed the largest variations in lifetime values between gram-negative bacteria (Supplementary Fig. 20). Altogether, these results confirmed (1) the full compatibility of TA fluorophores with solid-phase peptide chemistry, (2) the differential compartmentalization of TA fluorophores and TA-peptides, and (3) the potential of TA-peptides to distinguish between ESKAPEE bacterial species using FLIM.

### Cross-reactive sensor arrays combining TA fluorophores and TA-peptides enable lifetime-based assignation of ESKAPEE bacterial species

Given the different environments for TA fluorophores and TA-peptides in bacterial cells, we designed cross-reactive lifetime sensor arrays that could correctly assign all seven ESKAPEE bacterial species by combining DNA-binding TA fluorophores and cell envelope-binding TA-peptides. In order to generate arrays with excellent discriminatory power and yet the smallest number of molecular components, we performed a two-component PCA using the imaging datasets of the initial four best TA fluorophores (**4b,** **7b,** **8d**, and **8e**)—we excluded **5b** and **8b** because they were the least contributive—and all six TA-peptides (**PPEP1-3** and **NPEP1-3**). We found that the TA-peptide pair (**PPEP1**-**NPEP1**) provided the best discrimination among bacteria, followed by the **PPEP2**-**NPEP2** pair and lastly the **PPEP3**-**NPEP3** (Supplementary Fig. 21). Therefore, we analyzed the performance of a six-member cross-reactive array (**4b,** **7b,** **8d,** **8e,** **PPEP1** and **NPEP1**) and found that the combination of TA fluorophores and TA-peptides could discern between species that were not clearly distinguished in the first TA fluorophore array, namely (1) the two gram-negative *E. coli* and *P. aeruginosa* and (2) the gram-positive *E. faecium* and the gram-negative *A. baumannii*. On the other hand, the array consisting of compounds **4b,** **7b,** **8d,** **8e,** **PPEP1**, and **NPEP1** lacked discrimination between the two gram-positive *S. aureus* and *E. faecium*.

**Fig. 5:**
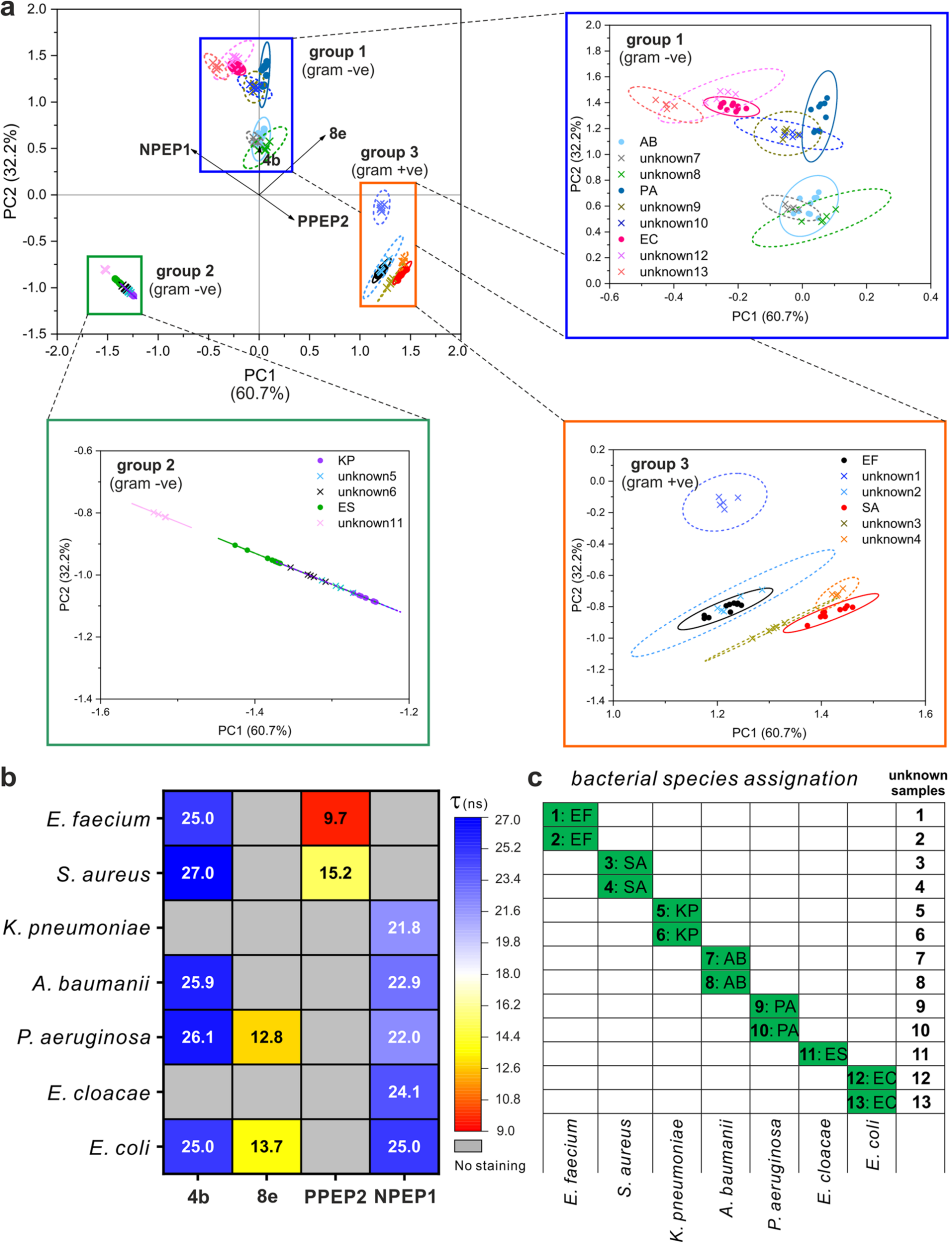
TA-based cross-reactive arrays enable lifetime-based assignation of ESKAPEE bacterial species. **a** PCA plots of the ESKAPEE bacterial panel (dots and solid confidence ellipses) and 13 unknown ESKAPEE samples (crosses and dashed confidence ellipses) using the optimal TA cross-reactive array (**4b,** **8e,** **PPEP1** and **NPEP2**). Results of 10 independent images are plotted from multiple repeats with 95% confidence intervals. **b** Pseudocolored heatmap of median lifetimes recorded for all ESKAPEE bacterial species with the optimal fluorescence lifetime cross-reactive array. Gray squares indicate the absence of cell labeling. **c** Bacterial species assignment of 13 unknown ESKAPEE samples using Random Forest analysis (experimental details in Supplementary Information and sample details in Supplementary Table 3).

To address this point, we replaced **PPEP1** with **PPEP2** because the latter exhibited substantially different lifetimes between *S. aureus* (~15 ns) and *E. faecium* (~10 ns). The resulting new array (**4b,** **7b,** **8d,** **8e,** **PPEP2,** **NPEP1**) was able to identify all seven ESKAPEE species with ≥95% confidence (Supplementary Fig. 21). Finally, we refined the array by considering the smallest number of TA fluorophores and TA-peptides without losing discrimination between bacterial species. For this purpose, we excluded the two least contributing TA fluorophores (**7b** and **8d**) and built a final array with only two TA fluorophores (**4b** and **8e**) and two TA-peptides (**PPEP2** and **NPEP1**). This final four-member array classified all seven ESKAPEE species across three main areas: one including the gram-negative *A. baumannii*, *P. aeruginosa*, and *E. coli*, a second one containing the gram-negative *E. cloacae* and *K. pneumoniae*, and a third one with only gram-positive *S. aureus* and *E. faecium* (Fig. 5a, b). Notably, the fluorescence lifetimes of the four-member cross-reactive array (**4b,** **8e,** **PPEP2,** **NPEP1**) retained the ≥95% confidence of the previous six-member array. Furthermore, we analyzed the sensing capabilities of the combined fluorescence intensity values—as opposite to lifetime values—of the same four TA components. As shown in Supplementary Fig. 22, the combined fluorescence intensity values could not distinguish most ESKAPEE bacterial species, highlighting the unique discriminatory power of lifetime-based cross-reactive arrays.

Having optimized a cross-reactive lifetime sensor array able to distinguish all seven ESKAPEE bacterial species, we examined its performance in 13 unknown samples of ESKAPEE bacteria where their species were blinded during the study (details in Methods). All samples were tested with the final four-member array (**4b,** **8e,** **PPEP2,** **NPEP1**), and their lifetime values were acquired under the same experimental procedures. For this, we developed a workflow that included incubation of the bacterial samples with the cross-reactive array, lifetime acquisition, and data analysis within a few hours (Supplementary Fig. 23). For the assignation of the bacterial species in the unknown samples, we employed the collected data in all seven ESKAPEE species as the training set and the supervised machine learning method Random Forest^64,65^. Random Forest analysis correctly assigned the bacterial species of all 13 ESKAPEE samples (Fig. 5c), even though the unknown samples included six different strains that were not part of the original training set (Supplementary Table 3). Altogether, these results highlight the resolving power of TA-based lifetime sensor arrays to characterize similar cells without extensive prior knowledge of molecular targets.

Finally, we assessed the performance of our cross-reactive array in clinically-relevant settings by spiking peripheral blood biosamples from healthy human donors with gram-positive *S. aureus*, gram-negative *E. coli*, or both pathogens. We utilized the final four-member array (**4b,** **8e,** **PPEP2,** **NPEP1**) and the same workflow for unknown samples (Supplementary Fig. 24). After plotting the results into the PCA space followed by Random Forest analysis, we correctly assigned both *S. aureus* and *E. coli* bacterial species to the different blood biosamples (Supplementary Fig. 25) as well as in spiked synthetic urine (Supplementary Fig. 26). These proof-of-concept results indicate the potential application of lifetime cross-reactive sensor arrays for the identification and assignation of ESKAPEE bacterial pathogens in complex biosamples.

## Discussion

This study presents a first-in-class cross-reactive chemical sensor array exclusively built with long-lifetime organic fluorophores. Cross-reactive arrays combine the readouts of multiple individual sensors (e.g., colorimetric dyes, biopolymers) to generate cell-specific fingerprints^8,9^. An important feature of cross-reactive arrays is that they do not always require prior knowledge of cellular targets, which enables one to discern between phenotypes that may not have been fully characterized or for which specific probes are unavailable. To date, most optical sensing arrays rely on intensity-based readouts (e.g., absorbance, fluorescence, chemiluminescence), which are hampered by their dependence on sensor concentration and limited reproducibility across biological systems.

The fluorescence lifetimes of organic fluorophores are excellent indicators of cellular microenvironments and -unlike intensity readouts- they are independent of experimental variables like fluorophore concentration^14^. To date, fluorescence lifetime arrays have been hampered by the lack of biocompatible fluorophores emitting diverse and long lifetimes. Conventional organic fluorophores (e.g., BODIPYs, cyanines, rhodamines) exhibit short lifetime values (i.e., <5 ns) overlapping with cell autofluorescence. Herein, we have synthesized a collection of 20 long-lifetime TA fluorophores by diversifying the triangulenium scaffold. Optimal synthetic routes for heteroatom and side-chain diversification resulted in four families of TA fluorophores with variable excitation/emission wavelengths (exc: 520-600 nm, em: 580–640 nm) and lifetime values ranging from 6 to 30 ns. Furthermore, TA fluorophores included water-solubilizing moieties (e.g., amines, carboxylic acids, ammonium, and sulfonate groups) for enhanced biocompatibility and reactive moieties for peptide tagging using SPPS.

We combined several TA fluorophores to generate lifetime arrays that could correctly assign all seven ESKAPEE bacterial species. By combining in vitro fluorescence assays and FLIM images of bacterial cultures, we confirmed that unmodified TA fluorophores bind intracellular bacterial DNA while TA-peptides, including vancomycin or colistin moieties, accumulate on the cell envelopes of gram-positive and gram-negative bacteria, respectively. Notably, the differential fluorescence lifetimes of TA fluorophores and TA-peptides enabled the optimization of the first lifetime-based cross-reactive sensor array able to distinguish all seven ESKAPEE bacterial species, which cannot be achieved by conventional fluorescence intensity-based measurements. We also demonstrated the application of our array by correctly assigning the bacterial species of 13 unknown ESKAPEE samples, even when different strains were tested. Furthermore, the relatively short assay time and avoidance of time-consuming bacterial cultures underscore its translational potential. We envision that TA-based probes will open avenues in pathogen surveillance and precision medicine, and that lifetime cross-reactive arrays will accelerate the design of novel sensing strategies to distinguish cell types, metabolic states, and phenotypes^15,16^, even when detailed information about their molecular targets is unavailable.

## Methods

All research complies with the relevant ethical regulations approved by the University of Edinburgh (25-EMREC-023).

### General information

Chemicals and solvents were purchased from commercial laboratory suppliers as AR grade and used without further purification. All amino acids were obtained from Iris Biotech GmbH. Microwave reactions were conducted using an Initiator+ microwave synthesizer (Biotage) under controlled conditions, with stirring at 1200 rpm and a constant temperature maintained as the reaction parameter. Thin-layer chromatography was performed on pre-coated Merck silica gel 60 F254 plates (under UV light at 254 nm and 365 nm). Column chromatography was performed using Merck 60 silica gel (particle size 0.040–0.063 mm) or a Teledyne Isco system, with eluent systems as specified. Automated Solid-Phase Peptide Synthesis (SPPS) was performed using a Biotage Alstra peptide synthesizer. HPLC-MS analysis was carried out on an Agilent 1260 Infinity system at 35 °C, utilizing a Kinetex XB-C_18_ column (4.6 mm × 50 mm, 5 μm). The mobile phases consisted of H₂O + 0.1% trifluoroacetic acid (TFA) and ACN + 0.1% TFA, with gradient elution at a flow rate of 20 mL min⁻¹. NMR spectra were acquired using 400 MHz, 500 MHz, or 600 MHz NMR spectrometers. Chemical shifts are reported in parts per million (ppm, δ) and are described as s (singlet), d (doublet), t (triplet), dd (doublet of doublets), m (multiplet), or br s (broad singlet), with reference to the solvent peak. High-resolution mass spectrometry (HRMS) data were obtained in ESI positive mode using an LTQ-FT Ultra mass spectrometer. MALDI-TOF mass spectrometry was conducted using a MALDI-TOF mass spectrometer, with the analysis focused on the appropriate base peak ion in positive reflector mode. Photophysical properties were characterized using a UV-1900i Shimadzu spectrophotometer for absorbance measurements and a Cytation3 Imaging Reader (BioTek) for emission studies. Fluorescence lifetime measurements were performed with a spectrofluorometer FS5 (Edinburgh Instruments) equipped with a 450 nm laser, while fluorescence lifetime imaging was carried out using a Leica SP8 FALCON system. Data analysis was conducted using Origin Pro 2024 and Python.

### Fluorescence lifetime measurements

Fluorescence lifetime values were measured using an FS5 spectrometer (Edinburgh Instruments) in time-correlated single photon counting mode. Samples were prepared at 10 μM concentration at the indicated conditions and excited using EPL-450 diode laser. The resulting fluorescence decays were fitted with single or double exponential decays.

### Fluorescence quantum yield measurements

Rhodamine 6G, rhodamine B, and cresyl violet were used as standards (e.g., respective quantum yields for rhodamine 6G (in EtOH), rhodamine B (in EtOH), and cresyl violet (in MeOH) are 0.94, 0.31, and 0.54). Probes were diluted independently from a 5 mM stock solution in ACN or H_2_O to produce samples with 5 concentration gradients. Emission spectra were recorded at excitation wavelengths that overlapped with the reference absorption. Quantum yields were calculated with the equation:

$${\Phi }_{S}={\Phi }_{R}\frac{{{Grad}}_{S}\,}{{{Grads}}_{R}}{\left(\frac{{n}_{S}}{{n}_{R}}\right)}^{2}$$

where Φ is the fluorescence quantum yield, Grad are gradients of the plot of integrated fluorescence intensity against absorbance under the excitation wavelength, n are the refractive index of the solvents used. Subscripts S and R refer to samples and standards, respectively.

### Bacteria labeling procedures

Bacterial colonies were picked from agar plates and inoculated into LB broth (*S. aureus*, *P. aeruginosa*, *E. coli*, and *K. pneumoniae*) or BHI broth (*E. faecium*, *A. baumannii*, and *E. cloacae*). Cultures were incubated for 4 h in 10 mL aliquots at 37 °C with shaking. The broth was removed, and the sample was washed three times with saline (centrifuged at 13,000 rpm for 3 min each time). The resulting bacterial pellet was resuspended in 4 mL of saline to get bacteria aliquot with an OD_600_ of 0.1. Bacteria were then stained with probes for 30 min (for TA fluorophores) or 15 min (for TA-peptides) at 37 °C. After this, the sample was washed three times and resuspended in 300 µL of saline. A 150 µL aliquot of the bacteria was added to µ-slide 18-well glass bottom chamber (IBIDI®) (pre-coated with poly-D-Lysine). The wells were allowed to stand at r.t. for 15 min before imaging.

### Blood biosample experiments

Blood was collected from healthy human volunteers (male and female volunteers, age range: 20–60 years-old) following written informed consent and ethical approval at the University of Edinburgh (25-EMREC-023). All donor samples were anonymized following blood sample collection, and information on age, sex, and gender was not available to the researchers. Colonies of *E. coli* (ATCC 25922) and *S. aureus* (USA300) were spiked into 1 mL human peripheral blood and mixed thoroughly. Deionized water was added to the blood-bacteria mixture (1:50 ratio). The mixture was kept at r.t. for 10 min, centrifuged at 4000 × *g* for 10 min, and the pellet was retained. This centrifugation step was repeated once more. The resulting pellet was cultured in 10 mL LB broth for 4 h. Before imaging, bacteria were washed three times with saline. The bacterial mixture (*S. aureus*: *E. coli* 1:1, OD_600_ = 0.2) was stained separately with **4b,** **8e,** **PPEP2**, and **NPEP1** at final concentrations of 5, 10, 2.5, and 2.5 µM, respectively (in saline buffer). Samples with **4b** and **8e** were incubated for 30 min at 37 °C, while those stained with **PPEP2** and **NPEP1** were incubated for 15 min at 37 °C. Bacteria were then washed twice with saline before imaging. As controls, *E. coli* and *S. aureus* were incubated in blood and stained with **4b,** **8e,** **PPEP2**, and **NPEP1** following the same protocol used for the mixed culture.

### Fluorescence intensity and FLIM microscopy

Both fluorescence intensity and lifetime images were acquired in a S5 Leica STELLARIS 8 FALCON FLIM confocal microscope with a 63×/1.40 oil lens. Image analysis was performed using Single Molecule Detection module of Leica Application Suite X (LAS X). FLIM images of bacteria incubated with TA fluorophores were recorded using excitation at 530 nm with a repetition rate of 2.5 MHz, or at 488, 523, and 580 nm with a repetition rate of 5 MHz, respectively. FLIM images were collected with line repetitions 16 and frame repetitions 4. The image format was 512 × 512 pixels (0.09 × 0.09 mm pixel size), and collection speed was 100 Hz. Tunable white light laser power was set to 3%. A minimum five FLIM images were collected for each sample. FLIM images were fitted using precise tail fit, using LAS X fluorescence lifetime image fitting module.

#### Synthetic procedures

Compounds **1** and **6** were prepared as previously reported in literature^32^.

10-(3-carboxypropyl)-9-(2,6-dimethoxyphenyl)-1,8-dimethoxy-9,10-dihydroacridin-9-ylium hexafluorophosphate (compound **2a**). Compound **1** (1 eq., 500 mg, 0.98 mmol), GABA (2.13 eq., 215 mg, 2.09 mmol), and DIPEA (1.1 eq., 185 µL, 1.08 mmol) were dissolved in NMP (10 mL), and the reaction mixture was stirred for 16 h at r.t. The reaction mixture was then added to an acidic (pH 2) saturated aqueous KPF_6_ solution (300 mL), which led to the precipitation of the crude. The crude was filtered out, washed with water (2 × 25 mL), and air-dried. Finally, the solid was washed with diethyl ether (2 × 25mL), and dried in vacuo to yield compound **2a** as red powder, which was used without further purification (327 mg, 60%).

9-(2,6-dimethoxyphenyl)-1,8-dimethoxy-10-(6-(4-methylphenylsulfonamido)hexyl)-9,10-dihydroacridin-9-ylium trifluoroacetate (compound **2b**). *N*-p-Toluylsulfonyl-hexamethylenediamine was synthesized as described in literature^66^. Compound **1** (1 eq., 500 mg, 0.98 mmol), N-p-toluylsulfonyl-hexamethylenediamine (2.13 eq., 564 mg, 2.09 mmol), and DIPEA (1.1 eq., 185 µL, 1.08 mmol) were dissolved in NMP (10 mL), and the reaction mixture was stirred for 16 h at r.t. The reaction mixture was then added to an acidic (pH 2) saturated aqueous KPF_6_ solution (300 mL), which led to the precipitation of the crude. The crude was filtered out, washed with water (300 mL), and air-dried. The crude was purified by preparative HPLC to yield compound **2b** as a red powder (371 mg, 51%).

5-(3-carboxypropyl)-1,13-dimethoxy-9-methyl-5,9-dihydroquinolino[2,3,4-kl]acridin-13b-ylium trifluoroacetate (compound **3a**). Compound **2a** (1 eq., 20 mg, 0.033 mmol), methylamine hydrochloride (37 eq., 247 mg, 1.22 mmol), and DIPEA (37 eq., 0.15 mL, 1.22 mmol) were dissolved in ACN (3 mL) in a sealed tube. The mixture was reacted at 60 °C for 4 h, then methylamine hydrochloride (37 eq., 277 mg, 1.37 mmol) was added, and the mixture was stirred at 60 °C for 16 h. The reaction mixture was let to cool down to r.t. and poured into an acidic (pH 2) saturated aqueous KPF_6_ solution to precipitate the crude. The crude was filtered out, washed with water (300 mL), and air-dried. The crude was purified by preparative HPLC to yield compound **3a** as a green powder (7 mg, 37%).

8-(3-carboxypropyl)-12-methyl-8,12-dihydrobenzo[ij]xantheno[1,9,8-cdef][2,7]naphthyridin-3a2-ylium trifluoroacetate (compound **4a**). Compound **3a** (7 mg) was added to molten pyridinium chloride (10 g) at 210 °C. The mixture was stirred at 210 °C for 15 min. The mixture was cooled down to r.t. and poured into acidic (pH 2) saturated aqueous KPF_6_ solution (300 mL) to precipitate the crude. The crude was filtered out, washed with water (300 mL), and air-dried. The crude was purified by preparative HPLC to yield compound **4a** as a red powder (4.6 mg, 80%).

^1^H NMR (500 MHz, CD_3_CN) δ 8.29 (t, J = 8.6 Hz, 1H), 8.12 (td, J = 8.4, 4.8 Hz, 2H), 7.86 (s, 1H), 7.77 (d, J = 8.6 Hz, 1H), 7.71 (d, J = 8.8 Hz, 1H), 7.60 (d, J = 8.6 Hz, 1H), 7.33 (t, J = 7.7 Hz, 2H), 4.75–4.60 (m, 2H), 4.10 (s, 3H), 2.69 (t, J = 6.6 Hz, 2H), 2.26–2.12 (m, 2H).

^13^C NMR (126 MHz, CD_3_CN) δ 173.5, 152.6, 152.5, 141.7, 140.9, 140.5, 139.8, 139.7, 138.5, 138.4, 111.6, 111.5, 110.3, 109.4, 109.2, 108.4, 108.3, 107.6, 107.4, 106.0, 105.8, 47.1, 35.1, 20.3.

HRMS (ESI+): m/z calc. for C_24_H_19_N_2_O_3_+: 383.1390; found: 383.1401.

5-(3-carboxypropyl)-1,13-dimethoxy-9-propyl-5,9-dihydroquinolino[2,3,4-kl]acridin-13b-ylium trifluoroacetate (compound **3b**). Compound **2a** (1 eq., 30 mg, 0.05 mmol) was placed in a sealable tube and dissolved in ACN (3 mL). Excess propylamine (60 eq., 0.3 mL, 3 mmol) was added. The mixture was stirred at 70 °C overnight. The mixture was cooled down to r.t. and poured into acidic (pH 2) saturated KPF_6_ aq. (300 mL) to precipitate the crude. The crude was filtered out, washed with water (300 mL), and air-dried. The crude was purified by preparative HPLC to yield compound **3b** as a green powder (12 mg, 40%).

8-(3-carboxypropyl)-12-propyl-8,12-dihydrobenzo[ij]xantheno[1,9,8-cdef][2,7]naphthyridin-3a2-ylium trifluoroacetate (compound **4b**). Compound **3b** (12 mg, 0.02 mmol) was added into molten pyridinium chloride (10 g) at 210 °C. The mixture was stirred at 210 °C for 15 min. The mixture was cooled down to r.t. and poured into acidic (pH 2) saturated aqueous KPF_6_ solution to precipitate the crude. The crude was filtered out, washed with water (300 mL), and air-dried. The crude was purified by preparative HPLC to yield compound **4b** as a red powder (8.4 mg, 80%).

^1^H NMR (500 MHz, CD_3_CN) δ 8.24 (t, J = 8.6 Hz, 1H), 8.05 (ddd, J = 8.8, 8.2, 5.0 Hz, 2H), 7.64 (d, J = 8.8 Hz, 1H), 7.58 (d, J = 8.6 Hz, 1H), 7.53 (d, J = 8.8 Hz, 1H), 7.45 (d, J = 8.7 Hz, 1H), 7.25 (d, J = 8.2 Hz, 2H), 4.51–4.43 (m, 2H), 4.43–4.31 (m, 2H), 2.68 (t, J = 6.6 Hz, 2H), 2.54 (m, 2H), 2.22–2.07 (m, 2H), 1.19 (t, J = 7.4 Hz, 3H).

^13^C NMR (126 MHz, CD_3_CN) δ 173.6, 152.4, 140.9, 140.8, 139.7, 139.7, 139.6, 138.5, 138.5, 111.5, 109.2, 109.0, 108.4, 108.3, 107.5, 107.4, 105.9, 105.7, 49.2, 47.1, 29.4, 20.0, 18.8, 10.1.

HRMS (ESI+): m/z calc. for C_26_H_23_N_2_O_3_+: 411.1703; found: 411.1699.

5-(3-carboxypropyl)-9-(3-(dimethylamino)propyl)-1,13-dimethoxy-5,9-dihydroquinolino[2,3,4-kl]acridin-13b-ylium trifluoroacetate (compound **3c**). Compound **2a** (1 eq., 40 mg, 0.066 mmol) was placed in a sealable tube and dissolved in ACN (3 mL). Excess dimethylaminopropylamine (60 eq., 409 mg, 3.96 mmol) was added. The mixture was stirred at 60 °C overnight. The mixture was cooled down to r.t. and then poured into an acidic (pH 2) saturated aqueous KPF_6_ solution to precipitate the crude. The crude was filtered out, washed with water (300 mL), and air-dried. The crude was purified by preparative HPLC to yield compound **3c** as a green powder (12 mg, 30%).

8-(3-carboxypropyl)-12-(3-(dimethylamino)propyl)-8,12-dihydrobenzo[ij]xantheno[1,9,8-cdef][2,7]naphthyridin-3a2-ylium trifluoroacetate (compound **4c**). Compound **3c** (12 mg, 0.022 mmol) was added to molten pyridinium chloride (10 g) at 210 °C. The mixture was reacted at 210 °C for 15 min, then was cooled down to r.t. and poured into an acidic (pH 2) saturated aqueous KPF_6_ solution to precipitate the crude. The crude was filtered out, washed with water (300 mL), and air-dried. The crude was purified by preparative HPLC to yield compound **4c** as a red powder (10 mg, 82%).

8-(3-carboxypropyl)-12-(3-(trimethylammonio)propyl)-8,12-dihydrobenzo[ij]xantheno[1,9,8-cdef][2,7]naphthyridin-3a2-ylium trifluoroacetate (compound **4d**). Compound **4c** (1 eq., 10 mg, 0.02 mmol) was dissolved in dry DMF (2 mL). Methyl iodide (1.5 eq., 2 μL, 0.03 mmol) and Na_2_CO_3_ (5 eq., 13 mg, 0.1 mmol) were added. The mixture was stirred at r.t. for 3 h. The mixture was purified by preparative HPLC to yield compound **4d** as a red powder (3.8 mg, 27%).

^1^H NMR (500 MHz, DMSO-d_6_) δ 8.27 (t, J = 8.5 Hz, 1H), 8.14 (t, J = 8.4 Hz, 1H), 8.08–7.91 (m, 3H), 7.80 (d, J = 8.9 Hz, 1H), 7.68 (d, J = 8.6 Hz, 1H), 7.41 (d, J = 8.1 Hz, 1H), 7.32 (s, 1H), 4.59 (t, J = 8.1 Hz, 2H), 4.48 (s, 2H), 3.72 (t, 2H), 3.16 (s, 9H), 2.31 (t, 2H), 2.28 (t, J = 5.6 Hz, 2H), 1.75-1.95 (m, 2H).

^13^C NMR (126 MHz, DMSO-d_6_) δ 174.4, 165.4, 152.2, 140.6, 140.1, 139.7, 138.9, 110.3, 109.8, 108.8, 108.5, 107.1, 106.1, 62.5, 53.1, 48.5, 44.8, 33.7, 22.3, 19.8.

HRMS (ESI+): m/z calc. for C_29_H_30_N_3_O_3_+, 468.2281; found: 468.2270.

3-((3-(12-(3-carboxypropyl)benzo[ij]xantheno[1,9,8-cdef][2,7]naphthyridin-3a2-ylium-8(12H)-yl)propyl)dimethylammonio)propane-1-sulfonate trifluoroacetate (compound **4e**). Compound **4c** (1 eq., 10 mg, 0.02 mmol) was dissolved in dry DMF (2 mL). Sultone (1.1 eq., 3 mg, 0.022 mmol) and DIPEA (1.1 eq., 4 μL, 0.022 mmol) were added. The mixture was stirred at r.t. for 2 days, then purified by preparative HPLC to yield compound **4e** as a red powder (4.4 mg, 32%).

^1^H NMR (500 MHz, MeOD) δ 8.31 (t, J = 8.5 Hz, 1H), 7.99 (ddd, J = 12.9, 10.5, 5.4 Hz, 3H), 7.81 (d, J = 8.9 Hz, 1H), 7.71 (d, J = 8.5 Hz, 1H), 7.63 (d, J = 8.9 Hz, 1H), 7.01 (t, J = 7.6 Hz, 2H), 4.69–4.50 (m, 6H), 3.70–3.58 (m, 3H), 3.22 (s, 6H), 2.93 (t, J = 6.7 Hz, 2H), 2.68–2.45 (m, 4H), 2.39–2.21 (m, 4H).

^13^C NMR (126 MHz, MeOD) δ 179.0, 159.9, 159.6, 142.8, 142.1, 141.8, 138.8, 137.3, 137.2, 136.8, 118.9, 113.0, 112.8, 107.6, 106.8, 105.5, 104.4, 102.8, 102.7, 62.6, 59.9, 54.8, 54.8, 50.3, 33.4, 22.4, 19.5, 18.6.

HRMS (ESI+): m/z calc. for C_31_H_34_N_3_O_6_S+: 576.2163; found: 576.2165.

5-(3-carboxypropyl)-1,13-dimethoxy-9-(2-morpholinoethyl)-5,9-dihydroquinolino[2,3,4-kl]acridin-13b-ylium trifluoroacetate (compound **3f**). Compound **2a** (1 eq., 40 mg, 0.07 mmol) was placed in a sealable tube and dissolved in MeCN (3 mL). Excess 4-(2-aminoethyl) morpholine (60 eq., 525 µL, 4.0 mmol) was added. The mixture was stirred at 60 °C overnight, then cooled down and purified by preparative HPLC to yield compound **3f** as a green powder (20 mg, 44%).

8-(3-carboxypropyl)-12-(2-morpholinoethyl)-8,12-dihydrobenzo[ij]xantheno[1,9,8-cdef][2,7]naphthyridin-3a2-ylium trifluoroacetate (compound **4f**).

Compound **3f** (20 mg, 0.031 mmol) was added to molten pyridinium chloride (10 g) at 210 °C. The mixture was reacted at 210 °C for 15 min. 5 mL water was added into the mixture, and the crude was purified by preparative HPLC to yield compound **4 f** as a reddish powder (15 mg, 81%).

^1^H NMR (500 MHz, CD_3_CN) δ 8.29 (t, J = 8.6 Hz, 1H), 8.13 (td, J = 8.5, 4.8 Hz, 2H), 7.73 (d, J = 8.8 Hz, 1H), 7.67 (d, J = 8.6 Hz, 2H), 7.59 (d, J = 8.4 Hz, 1H), 7.36 (dd, J = 8.1, 2.7 Hz, 2H), 4.77 (s, 2H), 4.60–4.52 (m, 2H), 3.74 (s, 4H), 3.05 (s, 4H), 2.81 (s, 4H), 2.69 (t, J = 6.6 Hz, 2H).

^13^C NMR (126 MHz, CD_3_CN) δ 173.6, 152.7, 141.1, 140.2, 139.9, 139.6, 138.7, 138.5, 111.7, 109.4, 109.1, 108.6, 108.4, 107.9, 107.7, 106.0, 53.4, 47.2, 29.4, 20.1.

HRMS (ESI+): m/z calc. for C_29_H_28_N_3_O_4_+, 482.2074; found: 482.2072.

4,8-dimethyl-12-(6-(4-methylphenylsulfonamido)hexyl)-8,12-dihydro-4H-benzo[1,8][2,7]naphthyridino[3,4,5,6-klmn]acridin-3a2-ylium trifluoroacetate (compound **5a**).

Compound **2b** (1 eq., 150 mg, 0.20 mmol) was placed in a sealable tube and dissolved in NMP (5 mL). Excess methylamine hydrochloride (25 eq., 340 mg, 5.04 mmol) and DIPEA (25 eq., 0.64 mL, 5.04 mmol) were added. The mixture was allowed to react at 210 °C. After 4 h, another portion of methylamine hydrochloride (12.5 eq., 168 mg, 2.52 mmol) was added. The mixture was stirred at 210 °C overnight. The reaction mixture was let to cool down to r.t. and then poured into an acidic (pH 2) saturated aqueous KPF_6_ solution to precipitate the crude. The crude was filtered out, washed with water (300 mL), and air-dried. The crude was purified by preparative HPLC to yield compound **5a** as a red powder (40 mg, 30%).

^1^H NMR (500 MHz, CD_3_CN) δ 7.91 (t, J = 8.5 Hz, 2H), 7.84 (t, J = 8.5 Hz, 1H), 7.76 (dd, J = 8.4, 1.9 Hz, 2H), 7.41 (dd, J = 8.5, 2.6 Hz, 2H), 7.03 (t, J = 8.5 Hz, 4H), 6.96 (d, J = 8.5 Hz, 2H), 5.77 (s, 1H), 3.96 (dd, J = 11.5, 5.4 Hz, 2H), 3.51 (s, 6H), 2.90 (t, J = 6.0 Hz, 2H), 2.43 (s, 3H), 1.71 (t, J = 8.4 Hz, 2H), 1.50 (dt, J = 29.0, 6.5 Hz, 6H).

^13^C NMR (126 MHz, CD_3_CN) δ 159.6, 159.3, 143.4, 140.5, 140.4, 139.6, 139.0, 137.6, 137.5, 137.3, 129.6, 126.8, 109.4, 105.1, 105.0, 105.0, 47.6, 42.7, 35.0, 29.1, 25.7, 25.5, 24.1, 20.5.

HRMS (ESI+): m/z calc. for C_34_H_35_N_4_O_2_S+: 563.2475; found: 563.2489.

4-(6-(4-methylphenylsulfonamido)hexyl)-8,12-dipropyl-8,12-dihydro-4H-benzo[1,8][2,7]naphthyridino[3,4,5,6-klmn]acridin-3a2-ylium trifluoroacetate (compound **5b**).

Compound **2b** (1 eq., 150 mg, 0.20 mmol) was placed in a sealable tube and dissolved in NMP (5 mL). Excess propylamine (25 eq., 420 μL, 5.04 mmol) was added. The mixture was allowed to react at 210 °C. After 4 h, another portion of propylamine (12.5 eq., 210 μL, 2.52 mmol) was added. The mixture was allowed to react at 210 °C overnight. Next, the reaction mixture was cooled down to r.t. and poured into an acidic (pH 2) saturated aqueous KPF_6_ solution to precipitate the crude. The crude was filtered out, washed with water (300 mL), and air-dried. The crude was purified by preparative HPLC to yield compound **5b** as a red powder (47 mg, 32%).

^1^H NMR (500 MHz, CD_3_CN) δ 8.01 (td, J = 8.6, 2.5 Hz, 3H), 7.73 (d, J = 8.3 Hz, 2H), 7.42–7.35 (m, 2H), 7.23 (dd, J = 8.6, 3.5 Hz, 4H), 7.18 (d, J = 8.5 Hz, 2H), 5.55 (t, J = 6.3 Hz, 1H), 4.20 (td, J = 11.1, 7.9 Hz, 6H), 2.88 (q, J = 6.5 Hz, 2H), 2.41 (s, 3H), 1.93–1.87 (m, 4H), 1.85–1.77 (m, 2H), 1.61–1.39 (m, 6H), 1.18 (t, J = 7.4 Hz, 6H).

^13^C NMR (126 MHz, CD_3_CN) δ 143.4, 140.4, 140.3, 140.2, 137.6, 137.5, 129.6, 126.8, 110.4, 105.1, 105.0, 49.1, 47.6, 42.7, 29.1, 25.8, 25.5, 24.2, 20.4, 18.0, 10.0.

HRMS (ESI+): m/z calc. for C_38_H_43_N_4_O_2_S+: 619.3101; found: 619.3108.

4,8-bis(3-(dimethylamino)propyl)-12-(6-(4-methylphenylsulfonamido)hexyl)-8,12-dihydro-4H-benzo[1,8][2,7]naphthyridino[3,4,5,6-klmn]acridin-3a2-ylium trifluoroacetate (compound **5c**). Compound **2b** (1 eq., 150 mg, 0.20 mmol) was placed in a sealable tube and dissolved in NMP (5 mL). Excess dimethylaminopropylamine (25 eq., 640 µL, 5.04 mmol) was added. The mixture was allowed to react at 210 °C overnight. The reaction mixture was cooled down to r.t. and then poured into an acidic (pH 2) saturated aqueous KPF_6_ solution to precipitate the crude. The crude was filtered out, washed with water (300 mL), and air-dried. The crude was purified by preparative HPLC to yield compound **5c** as a red powder (17 mg, 10%).

4-(6-(4-methylphenylsulfonamido)hexyl)-8,12-bis(3-(trimethylammonio)propyl)-8,12-dihydro-4H-benzo[1,8][2,7]naphthyridino[3,4,5,6-klmn]acridin-3a2-ylium trifluoroacetate (compound **5d**). Compound **5c** (1 eq., 10 mg, 0.013 mmol) was dissolved in dry DMF (2 mL). Methyl iodide (3.3 eq., 3 μL, 0.043 mmol) and Na_2_CO_3_ (10 eq., 13 mg, 0.13 mmol) were added. The reaction was allowed to react at r.t. for 3 h. The mixture was purified by preparative HPLC to yield compound **5d** as a red powder (2.8 mg, 20%).

^1^H NMR (500 MHz, D_2_O) δ 8.00 (dt, J = 16.7, 8.5 Hz, 3H), 7.39 (m, 2H), 7.20 (d, J = 7.9 Hz, 2H), 7.15–7.03 (m, 6H), 4.11 (m, 4H), 3.92 (m, 2H), 3.54–3.47 (m, 4H), 3.00 (s, 18H), 2.64 (m, 2H), 2.21 (m, 3H), 2.13 (m, 4H), 1.61 (s, 2H), 1.25 (d, J = 39.8 Hz, 6H).

^13^C NMR (126 MHz, D_2_O) δ 170.9, 160.2, 144.7, 139.9, 139.6, 139.2, 138.1, 138.0, 135.4, 129.9, 126.2, 109.7, 109.4, 105.9, 104.9, 104.5, 62.8, 52.9, 43.0, 42.0, 27.8, 24.8, 24.4, 20.4, 18.6.

HRMS (ESI+): m/z calc. for C_44_H_59_N_6_O_2_S_3_ (3+), 245.1468; found: 245.1467.

3,3’-(((12-(6-(4-methylphenylsulfonamido)hexyl)-4H-benzo[1,8][2,7]naphthyridino[3,4,5,6-klmn]acridine-3a2-ylium-4,8(12H)-diyl)bis(propane-3,1-diyl))bis(dimethylammonionediyl))bis(propane-1-sulfonate) trifluoroacetate (compound **5e**). Compound **5c** (1 eq., 20 mg, 0.024 mmol) was dissolved in dry DMF (1 mL). Sultone (4 eq., 12 mg, 0.096 mmol) and DIPEA (4 eq., 16 µL, 0.096 mmol) were added, and the mixture was allowed to react at r.t. for 2 days. The mixture was purified by preparative HPLC to yield compound **5e** as a red powder (2.5 mg, 10%).

^1^H NMR (500 MHz, D_2_O) δ 8.34 (s, 1H), 8.02–7.82 (m, 3H), 7.16 (t, J = 12.6 Hz, 2H), 7.07 (d, J = 7.9 Hz, 2H), 7.01 (d, J = 8.3 Hz, 2H), 6.97 (d, J = 7.8 Hz, 2H), 6.92 (d, J = 6.6 Hz, 2H), 4.01 (s, 4H), 3.69 (s, 2H), 3.52 (s, 4H), 3.38 (s, 4H), 3.05 (s, 12H), 2.80 (s, 4H), 2.49 (s, 2H), 2.09 (s, 10H), 1.40 (s, 2H), 1.25–1.02 (m, 6H).

^13^C NMR (126 MHz, D_2_O) δ 169.4, 144.2, 139.6, 139.4, 139.2, 138.1, 135.3, 129.7, 129.6, 126.0, 125.5, 109.4, 109.2, 105.5, 105.1, 104.6, 61.8, 59.8, 51.1, 47.1, 42.3, 28.1, 25.2, 24.8, 23.8, 20.5, 18.2.

HRMS (ESI+): m/z calc. for C_48_H_65_N_6_O_8_S_3_+, 949.4020; found: 949.4019.

4-(6-(4-methylphenylsulfonamido)hexyl)-8,12-bis(2-morpholinoethyl)-8,12-dihydro-4H-benzo[1,8][2,7]naphthyridino[3,4,5,6-klmn]acridin-3a2-ylium trifluoroacetate (compound **5f**).

Compound **2b** (60 mg, 0.080 mmol) was added to molten pyridinium chloride (10 g) at 210 °C. The mixture was reacted at 210 °C for 15 min, then cooled down to r.t. and poured into an acidic (pH 2) saturated aqueous KPF_6_ solution to precipitate the crude. The crude was filtered out, washed with water (300 mL), and air-dried. The crude was purified by preparative HPLC to yield compound **9** as a red powder (42 mg, 80%). Compound **9** (1 eq., 42 mg, 0.064 mmol) was placed in a sealable tube and dissolved in NMP (3 mL). Excess ethylenediamine (1 mL) was added. The mixture was allowed to react at 120 °C overnight. The mixture was purified by preparative HPLC to yield compound **10** as a red powder (12 mg, 26%). Compound **10** (1 eq., 12 mg, 0.017 mg) was dissolved in ACN (5 mL), and ditosylate (3 eq., 15 mg, 0.05 mmol), dry K_2_CO_3_ (6 eq., 15 mg, 0.10 mmol) were added to the mixture. The mixture was allowed to reflux for 4 h and purified by preparative HPLC to yield compound **5f** as a red powder (1.5 mg, 10%).

^1^H NMR (601 MHz, CD_3_CN) δ 8.04 (td, J = 8.5, 2.4 Hz, 3H), 7.75 (d, J = 8.3 Hz, 2H), 7.39 (d, J = 7.9 Hz, 2H), 7.32–7.16 (m, 6H), 6.27 (s, 1H), 4.42 (t, J = 7.0 Hz, 4H), 4.28–4.16 (m, 2H), 3.72–3.60 (m, 8H), 2.89 (t, J = 6.6 Hz, 2H), 2.82 (t, J = 7.2 Hz, 4H), 2.62 (s, 8H), 2.41 (s, 3H), 1.86–1.75 (m, 2H), 1.61–1.50 (m, 6H).

^13^C NMR (151 MHz, CD_3_CN) δ 166.1, 143.2, 140.6, 140.3, 137.8, 137.7, 137.5, 129.6, 126.8, 110.4, 105.4, 105.3, 105.3, 66.5, 53.8, 53.1, 46.1, 42.7, 29.1, 25.7, 25.5, 20.4.

HRMS (ESI+): m/z calc. for C_44_H_53_N_6_O_4_S+, 761.3844; found: 761.3831.

8,12,12-trimethyl-8,12-dihydrobenzo[1,8]isochromeno[3,4,5,6-klmn]acridin-3a2-ylium trifluoroacetate (compound **7a**). Compound **6** (1 eq., 300 mg, 0.707 mmol) was dissolved in NMP (3 mL). Methylamine (33% in methanol) (3 eq., 260 μL, 19 mmol) was added to the mixture. The mixture was heated to 90 °C in the microwave for 2 h. Another portion of methylamine (33% in methanol) (1.5 eq., 130 μL, 9.465 mmol) was added and reacted for another 2 h. Next, the mixture was cooled down to r.t. and poured into an acidic (pH 2) saturated aqueous KPF_6_ solution to precipitate the crude. The crude was filtered out, washed with water (300 mL), and air-dried. The crude was purified by preparative HPLC to yield compound **7a** as a reddish powder (108 mg, 35%).

^1^H NMR (500 MHz, CD_3_CN) δ 8.42 (dd, J = 9.0, 8.1 Hz, 1H), 8.35 (dd, J = 9.0, 7.5 Hz, 1H), 8.18 (d, J = 9.0 Hz, 1H), 8.13–8.01 (m, 2H), 7.95 (d, J = 8.9 Hz, 1H), 7.90 (dd, J = 7.7, 0.9 Hz, 1H), 7.67–7.56 (m, 2H), 4.41 (s, 3H), 1.92 (s, 6H).

^13^C NMR (126 MHz, CD_3_CN) δ 153.4, 148.8, 148.6, 143.2, 140.7, 139.7, 138.7, 137.7, 124.1, 123.7, 115.2, 115.2, 114.4, 112.4, 109.6, 108.4, 40.5, 37.1, 34.5.

HRMS (ESI+): m/z calc. for C_23_H_18_NO+: 324.1383; found: 324.1377.

12,12-dimethyl-8-propyl-8,12-dihydrobenzo[1,8]isochromeno[3,4,5,6-klmn]acridin-3a2-ylium trifluoroacetate (compound **7b**). Compound **6** (1 eq., 300 mg, 0.707 mmol) was dissolved in NMP (3 mL). Propylamine (3 eq., 175 µL, 2.12 mmol) was added. The mixture was heated to 90  °C in the microwave for 4 h. Then the mixture was cooled down to r.t. and poured into an acidic (pH 2) saturated aqueous KPF_6_ solution to precipitate the crude. The crude was filtered out, washed with water (300 mL), and air-dried. The crude was purified by preparative HPLC to yield compound **7b** as a reddish powder (148 mg, 45%).

^1^H NMR (500 MHz, CD_3_CN) δ 8.40 (dd, J = 9.0, 8.1 Hz, 1H), 8.34 (dd, J = 9.0, 7.5 Hz, 1H), 8.16 (dd, J = 9.0, 0.7 Hz, 1H), 8.11–8.01 (m, 2H), 7.98–7.93 (m, 1H), 7.90 (dd, J = 7.9, 0.9 Hz, 1H), 7.61 (ddd, J = 17.1, 8.2, 0.7 Hz, 2H), 4.94–4.76 (m, 2H), 2.10 (dq, J = 16.3, 7.4 Hz, 2H), 1.91 (s, 6H), 1.25 (t, J = 7.4 Hz, 3H).

^13^C NMR (126 MHz, CD_3_CN) δ 153.4, 152.9, 149.0, 148.5, 143.2, 141.2, 140.0, 139.7, 138.8, 137.7, 124.2, 123.7, 115.2, 115.0, 114.6, 112.6, 112.3, 109.5, 108.4, 50.7, 40.5, 34.6, 20.2, 10.1.

HRMS (ESI+): m/z calc. for C_25_H_22_NO+: 352.1696; found: 352.1687.

8-(3-(dimethylamino)propyl)-12,12-dimethyl-8,12-dihydrobenzo[1,8]isochromeno[3,4,5,6-klmn]acridin-3a2-ylium trifluoroacetate (compound **7c**). Compound **6** (1 eq., 200 mg, 0.472 mmol) was dissolved in NMP (3 mL) in a sealed tube. Dimethylaminopropylamine (3 eq., 180 µL, 1.414 mmol) was added. The reaction was heated to 90 °C in the microwave for 4 h. Then the mixture was cooled down to r.t. and poured into an acidic (pH 2) saturated aqueous KPF_6_ solution to precipitate the crude. The crude was filtered out, washed with water (300 mL) and air-dried. The crude was purified by preparative HPLC to yield compound **7c** as a reddish powder (29 mg, 12%).

12,12-dimethyl-8-(3-(trimethylammonio)propyl)-8,12-dihydrobenzo[1,8]isochromeno[3,4,5,6-klmn]acridin-3a2-ylium trifluoroacetate (compound **7d**). Compound **7c** (1 eq., 200 mg, 0.370 mmol) was dissolved in dry DMF (5 mL). Methyl iodide (1.1 eq., 25 μL, 0.407 mmol) and Na_2_CO_3_ (5 eq., 7 mg, 0.05 mmol) were added, and the mixture was stirred at r.t. for 3 h. The mixture was purified by preparative HPLC to yield compound **7d** as a reddish powder (35 mg, 15%).

^1^H NMR (600 MHz, acetone-d_6_) δ 8.57–8.52 (m, 2H), 8.45 (dd, J = 9.0, 7.5 Hz, 1H), 8.34 (d, J = 8.9 Hz, 1H), 8.28 (dd, J = 7.5, 0.7 Hz, 1H), 8.17 (dd, J = 8.3, 7.8 Hz, 1H), 8.07 (dd, J = 7.9, 0.9 Hz, 1H), 7.79 (dd, J = 8.1, 0.6 Hz, 1H), 7.72 (dd, J = 8.3, 0.9 Hz, 1H), 5.32 (m, J = 8.2 Hz, 2H), 4.30–4.09 (m, 2H), 3.45 (s, 9H), 2.91 (m, J = 12.8, 8.8, 4.4 Hz, 2H), 2.00 (s, 6H).

^13^C NMR (151 MHz, acetone-d_6_) δ 153.5, 153.1, 149.3, 148.7, 143.8, 141.4, 140.4, 140.1, 139.4, 138.1, 124.5, 124.0, 115.3, 115.0, 114.6, 112.6, 112.3, 109.5, 108.8, 62.7, 62.7, 53.1, 53.1, 53.1, 45.9, 40.6, 34.7, 21.0.

HRMS (ESI+): m/z calc. for C_28_H_30_N_2_O_2_ (2+): 205.1174; found: 205.1173.

3-((3-(12,12-dimethylbenzo[1,8]isochromeno[3,4,5,6-klmn]acridin-3a2-ylium-8(12H)-yl)propyl)dimethylammonio)propane-1-sulfonate trifluoroacetate (compound **7e**). Compound **7c** (1 eq., 200 mg, 0.370 mmol) was dissolved in dry DMF (5 mL). Sultone (4 eq., 210 mg, 1.48 mmol) and DIPEA (4 eq., 260 µL, 1.48 mmol) were added. The mixture was stirred at r.t. for 3 days and purified by preparative HPLC to yield compound **7e** as a reddish powder (26 mg, 11%).

^1^H NMR (601 MHz, DMSO-d_6_) δ 8.52–8.48 (m, 1H), 8.46–8.40 (m, 2H), 8.26–8.22 (m, 2H), 8.10 (t, J = 8.0 Hz, 1H), 8.05–8.02 (m, 1H), 7.76 (d, J = 8.0 Hz, 1H), 7.72 (d, J = 8.3 Hz, 1H), 5.02 (s, 2H), 3.80–3.75 (m, 2H), 3.55–3.49 (m, 2H), 3.08 (s, 6H), 2.53 (t, J = 6.8 Hz, 2H), 2.44–2.36 (m, 2H), 2.18–2.11 (m, 2H), 1.90 (s, 6H).

^13^C NMR (151 MHz, DMSO-d_6_) δ 153.3, 152.8, 149.0, 148.7, 143.4, 141.1, 140.7, 140.0, 139.6, 138.3, 124.8, 124.4, 115.8, 115.6, 114.5, 112.6, 112.5, 110.2, 108.9, 63.3, 59.2, 51.0, 47.9, 46.3, 40.8, 35.5, 20.6, 19.5.

HRMS (ESI+): m/z calc. for C_30_H_33_N_2_O_4_S+: 517.2155; found: 517.2150.

12,12-dimethyl-8-(2-morpholinoethyl)-8,12-dihydrobenzo[1,8]isochromeno[3,4,5,6-klmn]acridin-3a2-ylium trifluoroacetate (compound **7f**). Compound **6** (1 eq., 30 mg, 0.075 mmol) was dissolved in NMP (2 mL). 4-(2-aminoethyl)morpholine (3 eq., 30 μL, 0.224 mmol) was added. The mixture was heated to 90 °C in the microwave for 3 h. Another portion of 4-(2-aminoethyl) morpholine (1 eq., 10 μL, 0.07 mmol) was added and reacted for another 2 h. The mixture was purified by preparative HPLC to yield compound **7f** as a reddish powder (6.5 mg, 16%).

^1^H NMR (500 MHz, CD_3_CN) δ 8.45 (dd, J = 8.8, 8.3 Hz, 1H), 8.36 (dd, J = 8.9, 7.6 Hz, 1H), 8.26 (d, J = 8.9 Hz, 1H), 8.13–8.05 (m, 3H), 7.93 (dd, J = 7.8, 0.8 Hz, 1H), 7.69 (d, J = 8.1 Hz, 1H), 7.64 (dd, J = 8.4, 0.8 Hz, 1H), 5.37–5.31 (m, 2H), 3.97 (t, J = 4.7 Hz, 4H), 3.56–3.51 (m, 2H), 3.33 (s, 4H), 1.92 (s, 6H).

^13^C NMR (126 MHz, CD_3_CN) δ 153.6, 153.2, 149.5, 148.7, 144.3, 141.4, 140.3, 140.0, 139.2, 138.2, 124.4, 123.9, 115.3, 114.6, 112.5, 112.3, 109.1, 109.0, 64.2, 52.4, 51.7, 44.0, 40.6, 34.5.

HRMS (ESI+): m/z calc. for C_28_H_27_N_2_O_2_+: 423.2067; found: 423.2060.

12,12-dimethyl-8-(6-aminohexyl)-8,12-dihydrobenzo[1,8]isochromeno[3,4,5,6-klmn]acridin-3a2-ylium trifluoroacetate (compound **7g**). Compound **6** (1 eq., 60 mg, 0.150 mmol) was dissolved in NMP (3 mL). N-Boc-1,6-hexanediamine (3 eq., 100 μL, 0.450 mmol) was added. The mixture was heated to 90 °C in the microwave for 4 h. Next, the mixture was cooled down to r.t. and poured into an acidic (pH 2) saturated aqueous KPF_6_ solution to precipitate the crude. The crude was filtered out, washed with water (300 mL), and air-dried. Then the crude was dissolved in MeCN:TFA (1:1) and reacted under r.t. for 30 min. The mixture was neutralized, and the crude was purified by preparative HPLC to yield compound **7g** as a reddish powder (28 mg, 30%).

^1^H NMR (500 MHz, CD_3_CN) δ 8.41 (dd, J = 8.9, 8.1 Hz, 1H), 8.34 (dd, J = 9.0, 7.5 Hz, 1H), 8.17 (d, J = 8.9 Hz, 1H), 8.11–8.07 (m, 2H), 8.04 (dd, J = 8.3, 7.8 Hz, 1H), 7.95 (d, J = 8.8 Hz, 1H), 7.89 (dd, J = 7.9, 0.9 Hz, 1H), 7.78 (s, 2H), 7.63–7.55 (m, 2H), 4.93–4.86 (m, 2H), 3.05–2.96 (m, 2H), 2.09–2.02 (m, 2H), 1.91 (s, 6H), 1.80–1.74 (m, 2H), 1.73–1.68 (m, 2H), 1.61–1.53 (m, 2H).

13 C NMR (126 MHz, CD_3_CN) δ 153.4, 152.9, 148.9, 148.5, 143.2, 141.2, 139.8, 138.9, 137.7, 124.2, 123.7, 115.2, 115.0, 112.3, 109.5, 108.4, 49.2, 40.5, 39.3, 34.6, 26.8, 26.4, 25.5.

HRMS (ESI+): m/z calc. for C_28_H_29_N_2_O+: 409.2274; found: 409.2277.

4-(6-((tert-butoxycarbonyl)amino)hexyl)-8,12,12-trimethyl-8,12-dihydro-4H-benzo[1,8]isoquinolino[3,4,5,6-klmn]acridin-3a2-ylium trifluoroacetate (compound **8a**). Compound **7a** (1 eq., 20 mg, 0.046 mmol) was dissolved in NMP (1 mL) in a sealed tube. N-Boc-1,6-hexanediamine (25 eq., 300 μL, 1.143 mmol) was added. The mixture was heated to 90 °C in the microwave for 14 h. Then, the mixture was cooled down to r.t. and poured into an acidic (pH 2) saturated aqueous KPF_6_ solution to precipitate the crude. The crude was filtered out, washed with water (300 mL), and air-dried. The crude was purified by preparative HPLC to yield compound **8a** as a blue powder (9 mg, 30%).

^1^H NMR (601 MHz, CD_3_CN) δ 8.31 (t, J = 8.5 Hz, 1H), 8.14–8.06 (m, 2H), 7.93–7.84 (m, 4H), 7.51 (t, J = 17.5, 5.7 Hz, 2H), 5.42–5.34 (m, 1H), 4.62 (s, 2H), 4.13 (s, 3H), 3.14–3.01 (m, 2H), 2.05–1.98 (m, 2H), 1.90 (s, 6H), 1.73–1.64 (m, 2H), 1.59–1.45 (m, 4H), 1.42 (s, 9H).

^13^C NMR (151 MHz, CD_3_CN) δ 155.9, 148.2, 148.1, 142.3, 141.6, 140.9, 140.6, 140.1, 138.5, 136.4, 136.3, 122.4, 122.3, 115.6, 114.3, 114.1, 114.0, 113.8, 104.9, 104.7, 77.9, 48.6, 40.0, 39.9, 39.5, 35.5, 30.0, 29.6, 27.6, 26.1, 26.0, 25.8, 25.5.

HRMS (ESI+): m/z calc. for C_34_H_40_N_3_O_2_+: 522.3115; found: 522.3101.

4-(6-((tert-butoxycarbonyl)amino)hexyl)-12,12-dimethyl-8-propyl-8,12-dihydro-4H-benzo[1,8]isoquinolino[3,4,5,6-klmn]acridin-3a2-ylium trifluoroacetate (compound **8b**). Compound **7b** (1 eq., 20 mg, 0.043 mmol) was dissolved in NMP (3 mL) in a sealed tube. N-Boc-1,6-hexanediamine (25 eq., 300 µL, 1.143 mmol) was added. The mixture was heated to 90 °C in the microwave for 14 h. The mixture was cooled down to r.t. and poured into an acidic (pH 2) saturated aqueous KPF_6_ solution to precipitate the crude. The crude was filtered out, washed with water (300 mL), and air-dried. The crude was purified by preparative HPLC to yield compound **8b** as a blue powder (8.6 mg, 30%).

^1^H NMR (601 MHz, CD_3_CN) δ 8.29 (t, J = 8.6 Hz, 1H), 8.10 (ddd, J = 9.0, 7.6, 1.6 Hz, 2H), 7.96–7.82 (m, 4H), 7.52 (t, J = 8.5 Hz, 2H), 5.33 (s, 1H), 4.59 (s, 4H), 3.09 (dd, J = 12.2, 6.2 Hz, 2H), 2.07–1.99 (m, 4H), 1.89 (s, 6H), 1.68 (dt, J = 15.0, 7.5 Hz, 2H), 1.56–1.48 (m, 4H), 1.42 (s, 9H), 1.24 (t, J = 7.4 Hz, 3H).

^13^C NMR (151 MHz, CD_3_CN) δ 159.5, 159.3, 155.9, 148.2, 148.2, 142.4, 140.7, 140.7, 140.1, 140.0, 138.5, 136.4, 136.3, 122.4, 115.7, 115.6, 114.2, 114.2, 113.8, 113.8, 104.7, 104.7, 77.9, 49.9, 48.6, 40.0, 39.9, 35.5, 29.6, 27.6, 26.0, 25.8, 25.5, 19.1, 10.1.

HRMS (ESI+): m/z calc. for C_36_H_44_N_3_O_2_+: 550.3428; found: 550.3433.

4-(6-((tert-butoxycarbonyl)amino)hexyl)-8-(3-(dimethylamino)propyl)-12,12-dimethyl-8,12-dihydro-4H-benzo[1,8]isoquinolino[3,4,5,6-klmn]acridin-3a2-ylium trifluoroacetate (compound **8c**). Compound **7c** (1 eq., 20 mg, 0.040 mmol) was dissolved in NMP (3 mL) in a sealed tube. N-Boc-1,6-hexanediamine (25 eq., 300 μL, 1.143 mmol) was added. The mixture was heated to 90 °C in the microwave for 14 h. The mixture was cooled down to r.t. and poured into an acidic (pH 2) saturated aqueous KPF_6_ solution to precipitate the crude. The crude was filtered out, washed with water (300 mL) and air-dried. The crude was purified by preparative HPLC to yield compound **8c** as a blue powder (5.6 mg, 20%).

4-(6-((tert-butoxycarbonyl)amino)hexyl)-12,12-dimethyl-8-(3-(trimethylammonio)propyl)-8,12-dihydro-4H-benzo[1,8]isoquinolino[3,4,5,6-klmn]acridin-3a2-ylium trifluoroacetate (compound **8d**). Compound **8c** (1 eq., 7 mg, 0.011 mmol) was dissolved in dry DMF (1 mL). Methyl iodide (3 eq., 2 µL, 0.030 mmol) and Na_2_CO_3_ (5 eq., 7 mg, 0.05 mmol) were added. The mixture was allowed to react at r.t. for 3 h. The crude was purified by preparative HPLC to yield compound **8d** as a blue powder (2.6 mg, 28%).

^1^H NMR (601 MHz, CD_3_CN) δ 8.34 (t, J = 8.6 Hz, 1H), 8.15–8.08 (m, 3H), 7.94–7.89 (m, 3H), 7.76 (d, J = 8.6 Hz, 1H), 7.57 (d, J = 8.6 Hz, 1H), 5.40 (s, 1H), 4.69 (d, J = 56.9 Hz, 4H), 3.98–3.93 (m, 2H), 3.19 (s, 9H), 3.09 (dd, J = 12.9, 6.5 Hz, 2H), 2.43 (dt, J = 16.3, 8.2 Hz, 2H), 2.04–2.00 (m, 2H), 1.90 (s, 6H), 1.71–1.67 (m, 2H), 1.57–1.49 (m, 4H), 1.42 (s, 9H).

^13^C NMR (126 MHz, CD_3_CN) δ 166.2, 148.3, 148.2, 142.6, 140.7, 140.6, 140.1, 140.0, 138.8, 136.6, 136.6, 122.6, 115.6, 114.3, 114.2, 114.0, 114.0, 105.2, 105.0, 77.9, 62.4, 53.1, 53.1, 53.1, 48.7, 45.0, 40.1, 39.8, 35.5, 29.6, 27.6, 26.1, 25.8, 25.6, 19.8.

HRMS (ESI+): m/z calc. for C_39_H_52_N_4_O_2_ (2+): 304.2039; found: 304.2046.

3-((3-(8-(6-((tert-butoxycarbonyl)amino)hexyl)-12,12-dimethyl-8,12-dihydro-4H-benzo[1,8]isoquinolino[3,4,5,6-klmn]acridin-3a2-ylium-4-yl)propyl)dimethylammonio)propane-1-sulfonate trifluoroacetate (compound **8e**). Compound **8c** (1 eq., 7 mg, 0.010 mmol) was dissolved in dry DMF (1 mL). Sultone (4 eq., 6 mg, 0.04 mmol) and DIPEA (4 eq., 7 µL, 0.04 mmol) were added. The mixture was allowed to react at r.t. for 3 days. The mixture was purified by preparative HPLC to yield compound **8e** as a blue powder (2 mg, 26%).

^1^H NMR (601 MHz, CD_3_CN) δ 8.36 (t, J = 8.6 Hz, 1H), 8.13 (ddd, J = 21.3, 8.8, 7.6 Hz, 2H), 8.04 (d, J = 8.7 Hz, 1H), 7.90 (ddd, J = 17.5, 8.3, 0.8 Hz, 3H), 7.71 (d, J = 8.6 Hz, 1H), 7.56 (d, J = 8.6 Hz, 1H), 5.35 (s, 1H), 4.63 (s, 4H), 3.77–3.71 (m, 2H), 3.71–3.65 (m, 2H), 3.12 (s, 6H), 3.09 (q, J = 6.5 Hz, 2H), 2.71 (t, J = 6.4 Hz, 2H), 2.51 (t, J = 8.3 Hz, 2H), 2.31–2.26 (m, 2H), 1.88 (s, 6H), 1.69 (t, J = 7.6 Hz, 2H), 1.57–1.48 (m, 4H), 1.42 (s, 9H), 1.35–1.28 (m, 2H).

^13^C NMR (126 MHz, CD_3_CN) δ 148.2, 142.6, 140.6, 140.5, 140.1, 140.0, 139.1, 136.8, 136.5, 122.6, 122.5, 115.6, 114.1, 113.9, 105.2, 62.7, 59.2, 51.3, 48.7, 46.9, 45.2, 40.1, 35.5, 29.6, 27.6, 26.1, 25.8, 25.6, 19.4, 19.2.

HRMS (ESI+): m/z calc. for C_41_H_55_N_4_O_5_+: 715.3888; found: 715.3867.

4-(6-((tert-butoxycarbonyl)amino)hexyl)-12,12-dimethyl-8-(2-morpholinoethyl)-8,12-dihydro-4H-benzo[1,8]isoquinolino[3,4,5,6-klmn]acridin-3a2-ylium trifluoroacetate (compound **8f**). Compound **6** (1 eq., 50 mg, 0.12 mmol) was dissolved in NMP (3 mL) in a sealed tube. N-Boc-1,6-hexanediamine (3 eq., 94 μL, 0.36 mmol) was added. The mixture was heated at 90 °C in a sealed tube for 4 h. Then the mixture was cooled down to r.t. and purified by preparative HPLC to yield compound **11** as a reddish powder (30 mg, 40%). Compound **11** (1 eq., 20 mg, 0.032 mmol) was dissolved in NMP (2 mL). Ethylenediamine (25 eq., 55 µL, 0.8 mmol) was added. The mixture was heated at 90 °C in a sealed tube for 14 h. The mixture was cooled down to r.t. and purified by preparative HPLC to yield compound **12** as a blue powder (5.7 mg, 27%). Compound **12** (1 eq., 30 mg, 0.046 mmol) was dissolved in dry ACN (3 mL). Ditosylate (1.5 eq., 30 mg, 0.068 mmol), dry K_2_CO_3_ (3 eq., 20 mg, 0.138 mmol) were added into the mixture. The mixture was allowed to reflux for 4 h. The mixture was filtered and then purified by preparative HPLC to yield compound **8f** as a blue powder (3.4 mg, 10%).

^1^H NMR (500 MHz, MeOD) δ 8.38 (t, J = 8.6 Hz, 1H), 8.16 (ddd, J = 8.8, 7.7, 5.9 Hz, 2H), 8.01–7.95 (m, 4H), 7.65 (dd, J = 15.2, 8.7 Hz, 2H), 5.19 (s, 1H), 3.79–3.75 (m, 4H), 3.11 (t, J = 6.7 Hz, 2H), 3.01 (t, J = 7.5 Hz, 2H), 2.75–2.71 (m, 4H), 2.21 (dd, J = 16.3, 8.5 Hz, 2H), 2.09–2.02 (m, 2H), 1.94 (s, 6H), 1.73 (dt, J = 14.9, 7.6 Hz, 2H), 1.64–1.51 (m, 6H), 1.44 (s, 9H).

^13^C NMR (126 MHz, MeOD) δ 148.3, 140.7, 140.3, 140.1, 138.6, 136.5, 136.3, 122.5, 113.6, 104.8, 104.6, 66.5, 53.7, 53.5, 46.0, 40.0, 35.3, 27.4, 26.1, 25.7, 22.3.

HRMS (ESI+): m/z calc. for C_39_H_49_N_4_O_3_+: 621.3799; found: 621.3779.

3-((3-(8-(6-((azido)hexyl)-12,12-dimethyl-8,12-dihydro-4H-benzo[1,8]isoquinolino[3,4,5,6-klmn]acridin-3a2-ylium-4-yl)propyl)dimethylammonio)propane-1-sulfonate trifluoroacetate (compound **8g**). Compound **8e** (1 eq., 10.0 mg, 0.014 mmol) was dissolved in 4 M HCl in dioxane, and stirred at r.t. for 30 min. The solvent was evaporated, then imidazole-1-sulfonyl azide HCl salt (1.2 eq., 2.9 mg, 0.017 mmol), K_2_CO_3_ (3.7 eq., 7 mg, 0.051 mmol), CuSO4 (2 mg), and MeOH (1 mL) were added, and the mixture was stirred at r.t. for 2 h. Crude compound **8g** was used for CuAAC reactions with peptides without further purification.

#### Peptide synthesis

##### Synthesis of Fmoc-L-Dab(NH_2_)-OAll

Fmoc-L-Dab(NH-Boc)-OH (1 eq., 0.5 g, 1.13 mmol), allyl bromide (1.2 eq., 120 μL, 1.36 mmol), DIPEA (1.2 eq., 240 μL, 1.36 mmol) were dissolved in 5 mL DMF. After reacting at r.t. for 18 h, the solution was precipitated in cold Et_2_O. The crude was washed 3 times with Et_2_O, then dried in air, before purification by flash chromatography (cyclohexane:EtOAc, 1:1). The Boc-protected amino acid was then treated with 4 M HCl in dioxane for 1 h at r.t. Removal of the volatiles yielded Fmoc-L-Dab(NH_2_)-OAll as the HCl salt (383 mg, 89%).

##### Resin loading and capping

2-Chlorotrityl chloride polystyrene resin (2-CTC, 1 eq., 610 mg, 1.55 mmol g^−1^) was swollen in DCM. Fmoc-L-Dab (NH_2_)-OAll (1.3 eq., 383.2 mg, 1.00 mmol) was dissolved in DCM (10 mL), and DIPEA (3 eq., 0.42 mL, 3.0 mmol) was added to the resin, which was shaken at r.t. overnight. The resin was then washed with DCM (3 × 10 mL). 10 mL capping solution (DIPEA:DCM:MeOH, 1:5:4) was added to the resin and shaken for 30 min at r.t. The resin was then filtered off, washed with DCM (3 × 10 mL), and dried *in vacuo*.

##### Fmoc removal

Fmoc removal was performed using 20% piperidine in DMF (3 + 10 min at r.t.), then washed 4 times with 9 mL DMF.

##### Amino acid couplings

Fmoc-AA-OH (3 eq.), DIC (3 eq.), and Oxyma (3 eq.) in DMF were added to the deprotected resins. The mixtures were allowed to react 1 h. The resins were washed 3 times with 9 mL DMF. Amino acids used: Fmoc-L-Dab (Boc)-OH, Fmoc-L-Leu-OH, Fmoc-D-Leu-OH, Fmoc-L-Dab(Boc)-OH, Dde-L-Dab(Fmoc)-OH, Fmoc-L-Thr(tBu)-OH.

##### N-terminal capping

A solution of pyridine:Ac_2_O:DMF (10:15:75) was added, and the resin was vortexed for 15 min at r.t., followed by 3 washes with 9 mL DMF.

##### All deprotection

The peptidyl resin Dde-Dab(Thr(tBu))-Dab(Boc)-leu-Leu-Dab(Boc)-Dab(2CTC-resin)-OAll was added into a solution of Pd(PPh_3_)_4_ (10%, 18.6 mg, 0.1 mmol), PhSiH_3_ (3 eq., 37 μL, 0.3 mmol), DCM (5 mL) and shaken at r.t. for 2 h. The resin was then washed with DCM (3 × 10 mL) and DMF (3 × 10 mL).

##### On-resin macrocyclization

DIC (3 eq., 2 M, 2 mL), Oxyma (3 eq., 2 M, 2 mL), and DMF (2 mL) were added to the peptidyl resin and shaken at r.t. for 1 h. The resin was then filtered off and washed with DMF (3×8 mL).

##### Dde removal

Dde removal was performed using hydroxylamine hydrochloride (0.12 g, 1.7 mmol), and imidazole (0.09 g, 1.3 mmol) in 4 mL DMF at r.t. for 3 h.

#### Synthesis of TA-peptides

The procedure above yielded 766 mg of resin-bound protected cyclised colistin bearing an N-terminal Fmoc group for further derivatization (loading: 0.2 mmol g^−1^).

##### NPEP1 peptide

Following Fmoc removal in 90 mg of colistin-bound resin, compound **4b** (5 mg, 1.1 eq.), DIC (3 eq., 2 M in DMF), and Oxyma (3 eq., 2 M in DMF) were added to the resin, and the resin was shaken at r.t. for 18 h. The resin was then washed with DMF (3 × 9 mL), DCM (3 × 9 mL), and dried in vacuo. Cleavage from resin and deprotection was performed by adding 4 mL TFA:TIS:H_2_O (95:2.5:2.5) and shaking at r.t. for 1 h. The resin was filtered off and treated once more with cleavage cocktail for 1 h. Upon completion, the resin was filtered off, the filtrates were combined, and the crude **NPEP1** was precipitated in cold Et_2_O (30 mL). The crude was then centrifuged and washed with cold Et_2_O (3 × 40 mL) to give crude **NPEP1**. Purification by preparative HPLC followed by lyophilisation yielded **NPEP1** as a red powder (5 mg, 45%).

HRMS (MALDI-TOF MS): m/z calc. 1321.7416; found: 1321.7481 [M + H +].

##### NPEP2 peptide

Following Fmoc removal in 110 mg of colistin-bound resin, a solution of 0.5 g glutaric anhydride and 0.8 mL pyridine in DMF (4 mL) was added and the suspension shaken at r.t. for 1 h. The resin was then filtered off and washed with DMF (3 × 9 mL). The resin was activated with DIC (3 eq., 2 M in DMF) and Oxyma (3 eq., 2 M in DMF) for 5 min at r.t., before compound **7g** (1.3 eq., 13 mg) was added, and the mixture shaken at r.t. for 18 h. The resin was then washed with DMF (3 × 9 mL), DCM (3 × 9 mL) and dried in vacuo. Cleavage from resin and deprotection was performed by adding 4 mL TFA:TIS:H_2_O (95:2.5:2.5) and shaking at r.t. for 1 h. The resin was filtered off and treated once more with cleavage cocktail for 1 h. Upon completion, the resin was filtered off, the filtrates were combined, and the crude **NPEP2** was precipitated in cold Et_2_O (30 mL). The crude was then centrifuged and washed with cold Et_2_O (3 × 40 mL) to give crude **NPEP2**. Purification by preparative HPLC followed by lyophilisation yielded **NPEP2** as a reddish powder (6 mg, 17%).

HRMS (MALDI-TOF MS): m/z calc. for 1433.8304; found: 1433.8379 [M + H +].

##### NPEP3 peptide

Following Fmoc removal in 145 mg of colistin-bound resin, 4-ethynylbenzoic acid (3 eq., 13 mg), DIC (3 eq., 2 M in DMF), and Oxyma (3 eq., 2 M in DMF) were added to the resin and shaken at r.t. for 1 h. The resin was then washed with DMF (3 × 9 mL) and DCM (3 × 9 mL) and dried *in vacuo*. Cleavage from resin and deprotection was performed by adding 4 mL TFA:TIS:H_2_O (95:2.5:2.5) and shaking at r.t. for 1 h. The resin was filtered off and treated once more with cleavage cocktail for 1 h. Upon completion, the resin was filtered off, the filtrates were combined, and the crude was precipitated in cold Et_2_O (30 mL) and used without further purification. To crude freshly prepared **8g** (5 mg, 0.008 mmol, 1 eq.) in 0.5 mL H_2_O was added CuSO_4_ (5 mg, 0.03 mmol, 4 eq.), sodium ascorbate (15.4 mg, 0.078 mmol, 10 eq.), and L-histidine (4.8 mg, 0.03 mmol, 4 eq.). This solution was added to a solution of crude peptide (36 mg, 0.012 mmol, 1.5 eq.) in 0.5 mL H_2_O. The mixture was stirred for 30 min at r.t. and purified by preparative HPLC to yield **NPEP3** as a blue powder (5 mg, 34%).

HRMS (MALDI-TOF MS): m/z calc. 1698.9433; found 1698.9378 [M + H +].

##### PPEP1 peptide

Vancomycin hydrochloride was purchased from Apollo Scientific and used without further purification. (4-(TBDMS)oxymethyl)phenylmethanamine was synthesized as reported^67^. Compound **4b** (1 eq., 22 mg, 0.042 mmol), (4-(TBDMS)oxymethyl)phenylmethanamine (1.1 eq., 11.6 mg, 0.046 mmol), DCC (1.1 eq., 9.53 mg, 0.046 mmol), DMAP (1.1 eq., 5.64 mg, 0.046 mmol) were dissolved in 5 mL ACN and reacted at r.t. for 2 days. The crude product was purified by preparative HPLC, yielding 25 mg of TA-linker. TBDMS deprotection was performed in 5 mL 1.0 M TBAF in THF and reacted at r.t. overnight. Next, the organic solvent was removed, and DCM (15 mL) was added to the crude and filtered. To the filtrate was added Dess-Martin periodinane (4.3 eq., 60 mg, 0.18 mmol) and the reaction was stirred at r.t. for 4 h. The reaction was quenched by addition of Na_2_S_2_O_3_ (0.6 g in 3 mL H_2_O) and NaHCO_3_ (1 g in 12 mL H_2_O) for 20 min. The mixture was extracted with DCM (3 × 15 mL), and the organic fractions were dried over Na_2_SO_4_, and then the volatiles removed in vacuo. The benzaldehyde-TA was used as obtained for the following step, and dissolved in 4 mL DMF, with vancomycin hydrochloride (1.5 eq., 93.6 mg, 0.063 mmol), and DIPEA (3 eq., 22 μL, 0.126 mmol) being added. The mixture was stirred at 55 °C for 4 h. Next, a solution of NaBH_3_CN (11 eq., 30 mg, 0.48 mmol) and TFA (3.2 eq., 10 μL, 0.134 mmol) in MeOH (1 mL) was added, and the mixture was stirred at r.t. for 2 h. The solution was purified by preparative HPLC to yield **PPEP1** as a red powder (12 mg, 13%).

HRMS (MALDI-TOF MS): m/z calc. 1961.6753; found, 1961.6782 [M + H +].

##### PPEP2 peptide

Vancomycin hydrochloride was purchased from Apollo Scientific and used without further purification. (4-(TBDMS)oxymethyl)phenylmethanamine was synthesized as reported^67^. Compound **7g** (1 eq., 20 mg, 0.040 mmol), 4-(hydroxymethyl)benzaldehyde (1.1 eq., 6.68 mg, 0.044 mmol), DCC (1.1 eq., 9.12 mg, 0.04 mmol), DMAP (1.1 eq., 5.4 mg, 0.044 mmol) were dissolved in 5 mL THF and reacted at r.t. for 18 h. The organic solvent was removed, and the crude was used directly for next step. To the crude, vancomycin hydrochloride (1.5 eq., 93.6 mg, 0.063 mmol), DIPEA (3 eq., 22 μL, 0.126 mmol) were dissolved in 4 mL DMF and added. The mixture was stirred at 55 °C for 4 h. Next, a solution of NaBH_3_CN (11 eq., 30 mg, 0.48 mmol), TFA (3.2 eq., 10 μL, 0.134 mmol) in MeOH (1 mL) was added, and the mixture was stirred at r.t. for 2 h. The solution was purified by preparative HPLC to yield **PPEP2** as a reddish powder (28 mg, 32%).

HRMS (MALDI-TOF MS): m/z calc.1974.6957; found 1974.7088 [M + H +].

##### PPEP3 peptide

Vancomycin hydrochloride was purchased from Apollo Scientific and used without further purification. (4-(TBDMS)oxymethyl)phenylmethanamine was synthesized as reported^67^. Vancomycin hydrochloride (1 eq., 200 mg, 0.135 mmol), 4-ethynylbenzaldehyde (1.5 eq., 26 mg, 0.20 mmol), DIPEA (3 eq., 70 μL, 0.405 mmol) were dissolved in 4 mL DMF. The mixture was allowed to react under 55 °C for 4 h, and added to a solution of NaBH3CN (11 eq., 30 mg, 0.48 mmol), TFA (3.2 eq., 10 μL, 0.134 mmol) in MeOH, and was allowed to react 2 h at r.t. The solution was purified by preparative HPLC to isolate alkyne-vancomycin as a white powder (130 mg). Compound **8g** (1 eq., 10 mg, 0.014 mmol), CuSO_4_ (4 eq., 8.7 mg, 0.055 mmol), sodium ascorbate (10 eq., 27.7 mg, 0.14 mmol), L-histidine (4 eq., 8.5 mg, 0.055 mmol), and alkyne-vancomycin (1.5 eq., 32.0 mg, 0.021 mmol) were dissolved 1 mL H_2_O and stirred at r.t. for 30 min. The solution was purified by preparative HPLC to yield **PPEP3** as a blue powder (5 mg, 12%).

HRMS (ESI+): m/z calc. 2204.8146; found 2204.8154 [M + H +].

### Reporting summary

Further information on research design is available in the Nature Portfolio Reporting Summary linked to this article.

## Supplementary information

Supplementary Information

Reporting Summary

Transparent Peer Review file

## Source data

Source data

## Data Availability

Data supporting the findings of this study are available from the corresponding author upon request. Source data are provided with this paper.
