# Supplementary material for: Lifetime chemical sensor arrays of organic fluorophores for bacterial fingerprinting
Source: Nat Commun. 2026 Apr 28;17:5806. doi: 10.1038/s41467-026-72342-7 (PMC13328534; doi:10.1038/s41467-026-72342-7)
Supplement: Supplementary file 1 — Supplementary Information [file 41467_2026_72342_MOESM1_ESM.pdf]

Supplementary Information

Lifetime chemical sensor arrays of organic fluorophores for bacterial fingerprinting

Yanzi Zhou, Charles Lochenie, Sheelagh Duncan, Jennifer Marshall, Matthieu Vermeren, David H. Dockrell, Bethany Mills, Marc Vendrell

Table of Contents

Chemical Structures and Synthetic Schemes

Supplementary Figures

Supplementary Tables

Supplementary Notes

NMR Spectra

Chemical Structures and Synthetic Schemes

10-(3-carboxypropyl)-9-(2,6-dimethoxyphenyl)-1,8-dimethoxy-9,10-dihydroacridin-9-ylium hexafluorophosphate (compound **2a**).

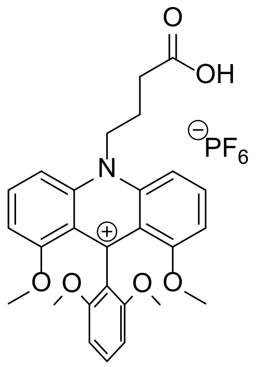

9-(2,6-dimethoxyphenyl)-1,8-dimethoxy-10-(6-(4-methylphenylsulfonamido)hexyl)-9,10-dihydroacridin-9-ylium trifluoroacetate (compound **2b**).

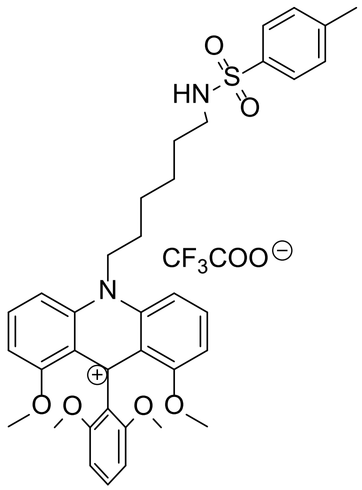

5-(3-carboxypropyl)-1,13-dimethoxy-9-methyl-5,9-dihydroquinolino[2,3,4-kl]acridin-13b-ylium trifluoroacetate (compound **3a**).

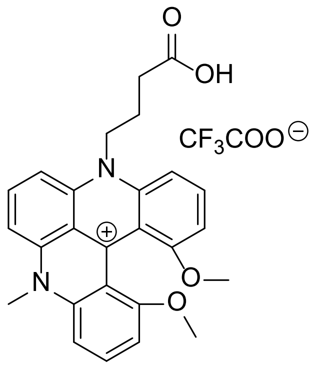

8-(3-carboxypropyl)-12-methyl-8,12-dihydrobenzo[ij]xantheno[1,9,8-cdef][2,7]naphthyridin-3a2-ylium trifluoroacetate (compound **4a**).

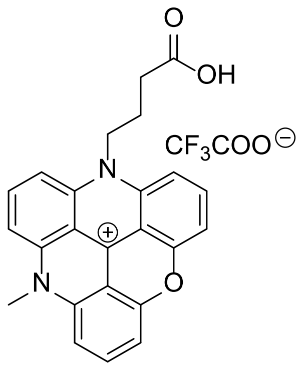

5-(3-carboxypropyl)-1,13-dimethoxy-9-propyl-5,9-dihydroquinolino[2,3,4-kl]acridin-13b-ylium trifluoroacetate (compound **3b**).

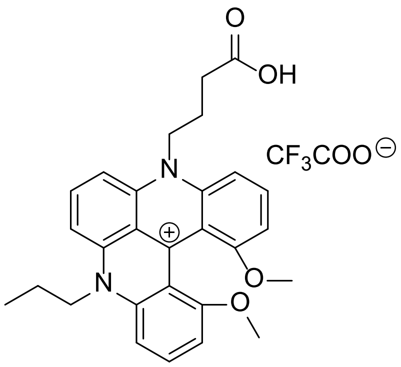

8-(3-carboxypropyl)-12-propyl-8,12-dihydrobenzo[ij]xantheno[1,9,8-cdef][2,7]naphthyridin-3a2-ylium trifluoroacetate (compound **4b**).

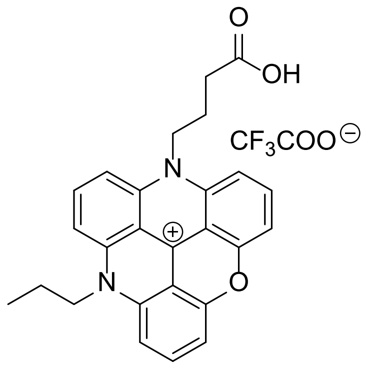

5-(3-carboxypropyl)-9-(3-(dimethylamino)propyl)-1,13-dimethoxy-5,9-dihydroquinolino[2,3,4-kl]acridin-13b-ylium trifluoroacetate (compound **3c**).

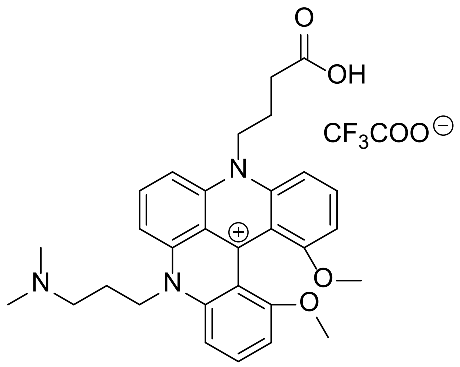

8-(3-carboxypropyl)-12-(3-(dimethylamino)propyl)-8,12-dihydrobenzo[ij]xantheno[1,9,8-cdef][2,7]naphthyridin-3a2-ylium trifluoroacetate (compound **4c**).

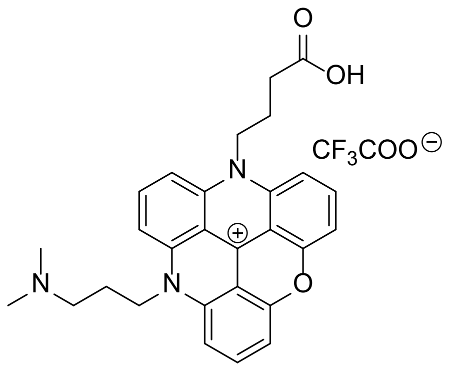

8-(3-carboxypropyl)-12-(3-(trimethylammonio)propyl)-8,12-dihydrobenzo[ij]xantheno[1,9,8-cdef][2,7]naphthyridin-3a2-ylium trifluoroacetate (compound **4d**).

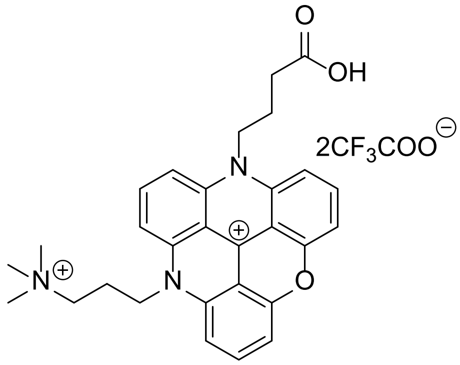

3-((3-(12-(3-carboxypropyl)benzo[ij]xantheno[1,9,8-cdef][2,7]naphthyridin-3a2-ylium-8(12H)-yl)propyl)dimethylammonio)propane-1-sulfonate trifluoroacetate (compound **4e**).

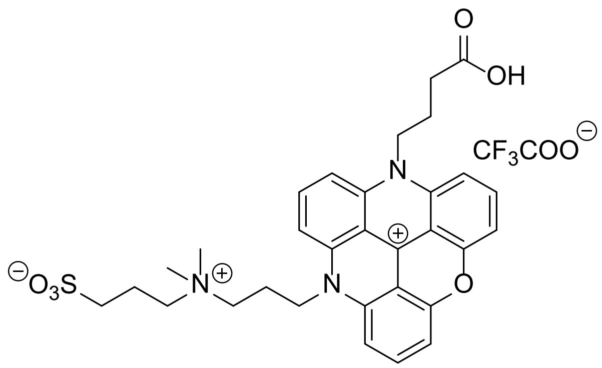

5-(3-carboxypropyl)-1,13-dimethoxy-9-(2-morpholinoethyl)-5,9-dihydroquinolino[2,3,4-kl]acridin-13b-ylium trifluoroacetate (compound **3f**).

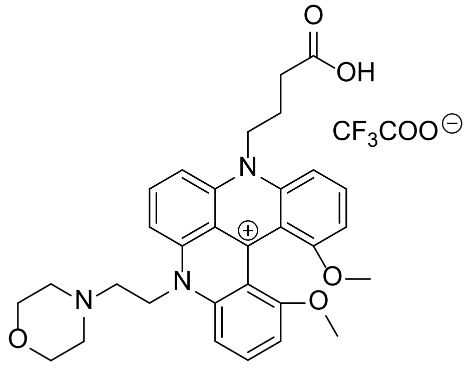

8-(3-carboxypropyl)-12-(2-morpholinoethyl)-8,12-dihydrobenzo[ij]xantheno[1,9,8-cdef][2,7]naphthyridin-3a2-ylium trifluoroacetate (compound **4f**).

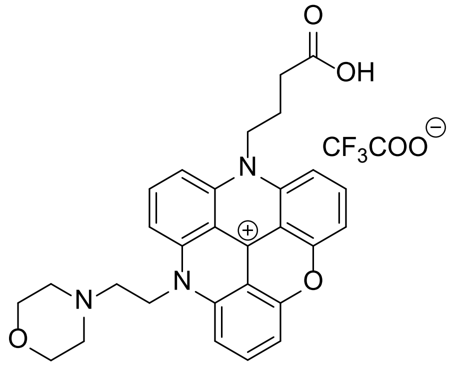

4,8-dimethyl-12-(6-(4-methylphenylsulfonamido)hexyl)-8,12-dihydro-4H-benzo[1,8][2,7]naphthyridino[3,4,5,6-klmn]acridin-3a2-ylium trifluoroacetate (compound **5a**).

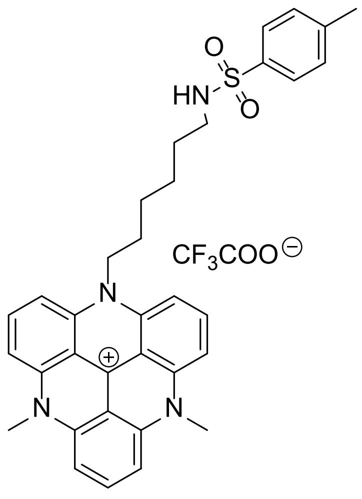

4-(6-(4-methylphenylsulfonamido)hexyl)-8,12-dipropyl-8,12-dihydro-4H-benzo[1,8][2,7]naphthyridino[3,4,5,6-klmn]acridin-3a2-ylium trifluoroacetate (compound **5b**).

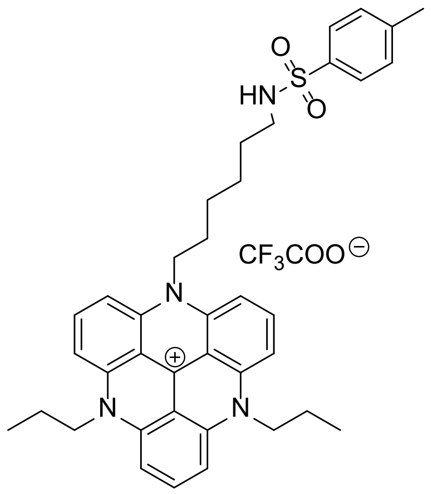

4,8-bis(3-(dimethylamino)propyl)-12-(6-(4-methylphenylsulfonamido)hexyl)-8,12-dihydro-4H-benzo[1,8][2,7]naphthyridino[3,4,5,6-klmn]acridin-3a2-ylium trifluoroacetate (compound **5c**).

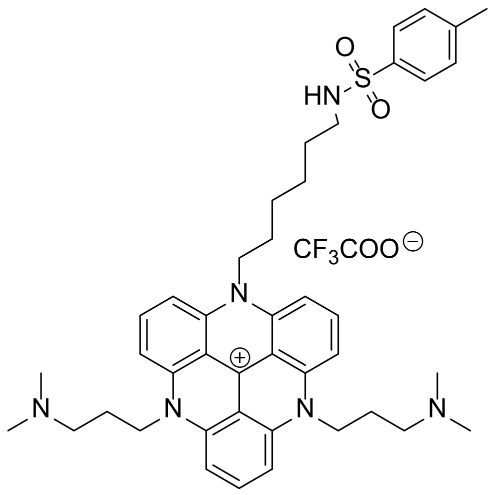

4-(6-(4-methylphenylsulfonamido)hexyl)-8,12-bis(3-(trimethylammonio)propyl)-8,12-dihydro-4H-benzo[1,8][2,7]naphthyridino[3,4,5,6-klmn]acridin-3a2-ylium trifluoroacetate (compound **5d**).

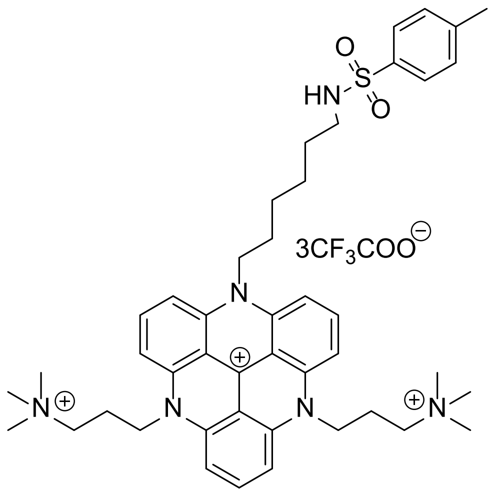

3,3'-(((12-(6-(4-methylphenylsulfonamido)hexyl)-4H-benzo[1,8][2,7]naphthyridino[3,4,5,6-klmn]acridine-3a2-ylium-4,8(12H)-diyl)bis(propane-3,1-diyl))bis(dimethylammonionediyl))bis(propane-1-sulfonate) trifluoroacetate (compound **5e**).

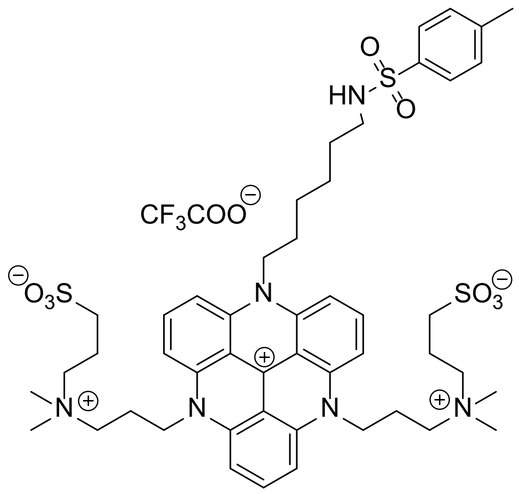

4-(6-(4-methylphenylsulfonamido)hexyl)-8,12-bis(2-morpholinoethyl)-8,12-dihydro-4H-benzo[1,8][2,7]naphthyridino[3,4,5,6-klmn]acridin-3a2-ylium trifluoroacetate (compound **5f**).

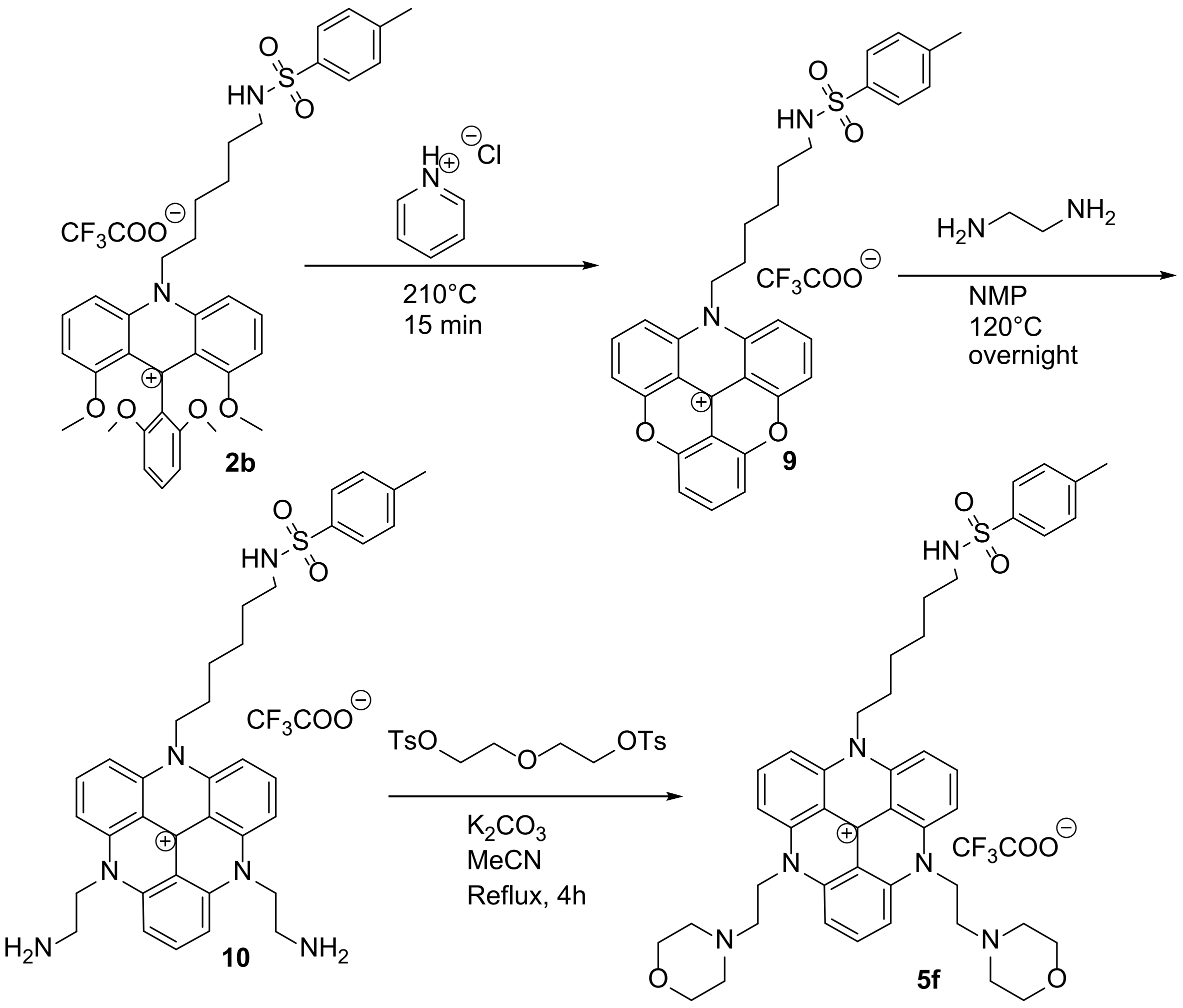

8,12,12-trimethyl-8,12-dihydrobenzo[1,8]isochromeno[3,4,5,6-klmn]acridin-3a2-ylium trifluoroacetate (compound **7a**).

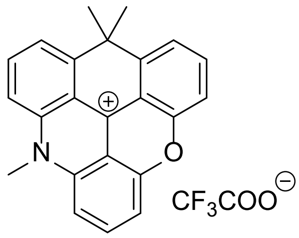

12,12-dimethyl-8-propyl-8,12-dihydrobenzo[1,8]isochromeno[3,4,5,6-klmn]acridin-3a2-ylium trifluoroacetate (compound **7b**).

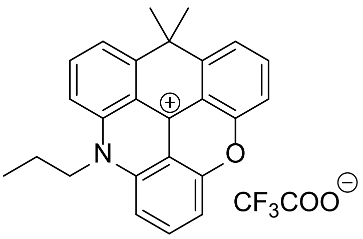

8-(3-(dimethylamino)propyl)-12,12-dimethyl-8,12-dihydrobenzo[1,8]isochromeno[3,4,5,6-klmn]acridin-3a2-ylium trifluoroacetate (compound **7c**).

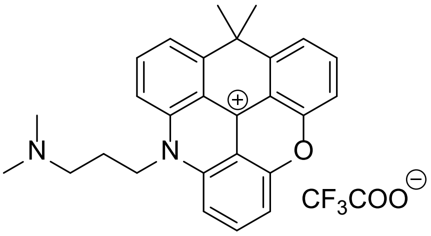

12,12-dimethyl-8-(3-(trimethylammonio)propyl)-8,12-dihydrobenzo[1,8]isochromeno[3,4,5,6-klmn]acridin-3a2-ylium trifluoroacetate (compound **7d**).

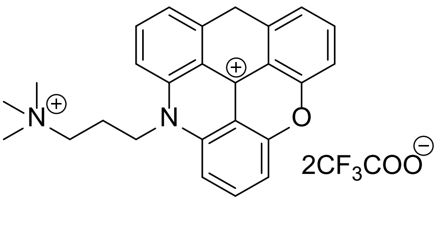

3-((3-(12,12-dimethylbenzo[1,8]isochromeno[3,4,5,6-klmn]acridin-3a2-ylium-8(12H)-yl)propyl)dimethylammonio)propane-1-sulfonate trifluoroacetate (compound **7e**).

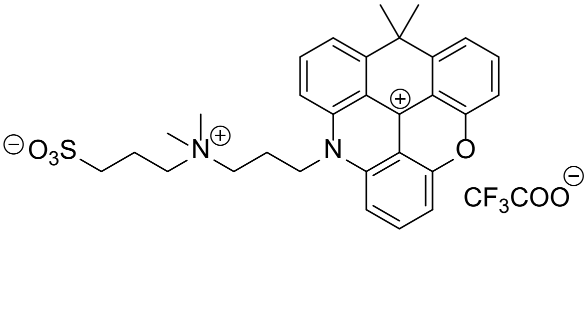

12,12-dimethyl-8-(2-morpholinoethyl)-8,12-dihydrobenzo[1,8]isochromeno[3,4,5,6-klmn]acridin-3a2-ylium trifluoroacetate (compound **7f**).

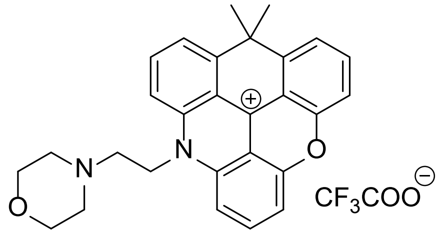

12,12-dimethyl-8-(6-aminohexyl)-8,12-dihydrobenzo[1,8]isochromeno[3,4,5,6-klmn]acridin-3a2-ylium trifluoroacetate (compound **7g**).

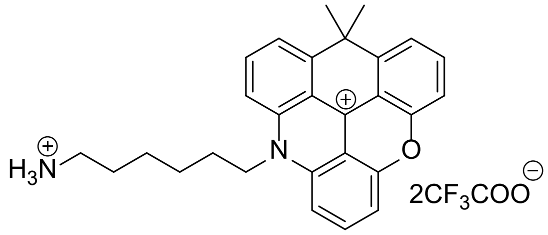

4-(6-((tert-butoxycarbonyl)amino)hexyl)-8,12,12-trimethyl-8,12-dihydro-4H-benzo[1,8]isoquinolino[3,4,5,6-klmn]acridin-3a2-ylium trifluoroacetate (compound **8a**).

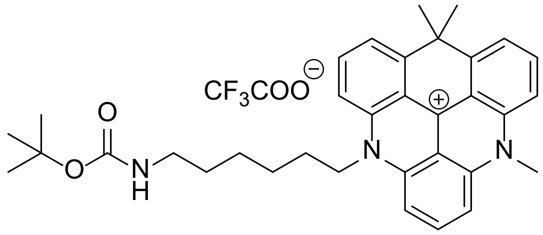

4-(6-((tert-butoxycarbonyl)amino)hexyl)-12,12-dimethyl-8-propyl-8,12-dihydro-4H-benzo[1,8]isoquinolino[3,4,5,6-klmn]acridin-3a2-ylium trifluoroacetate (compound **8b**).

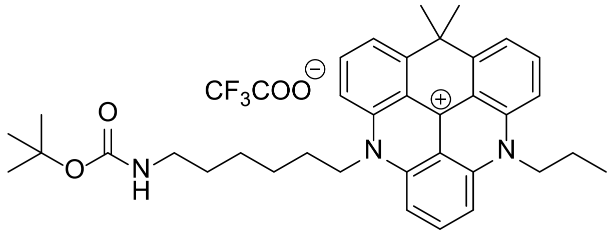

4-(6-((tert-butoxycarbonyl)amino)hexyl)-8-(3-(dimethylamino)propyl)-12,12-dimethyl-8,12-dihydro-4H-benzo[1,8]isoquinolino[3,4,5,6-klmn]acridin-3a2-ylium trifluoroacetate (compound **8c**).

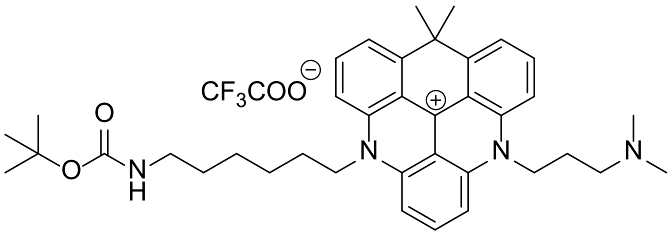

4-(6-((tert-butoxycarbonyl)amino)hexyl)-12,12-dimethyl-8-(3-(trimethylammonio)propyl)-8,12-dihydro-4H-benzo[1,8]isoquinolino[3,4,5,6-klmn]acridin-3a2-ylium trifluoroacetate (compound **8d**).

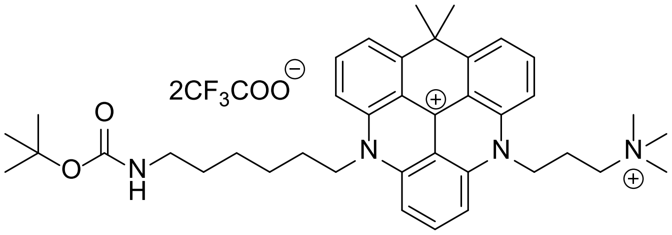

3-((3-(8-(6-((tert-butoxycarbonyl)amino)hexyl)-12,12-dimethyl-8,12-dihydro-4H-benzo[1,8]isoquinolino[3,4,5,6-klmn]acridin-3a2-ylium-4-yl)propyl)dimethylammonio)propane-1-sulfonate trifluoroacetate (compound **8e**).

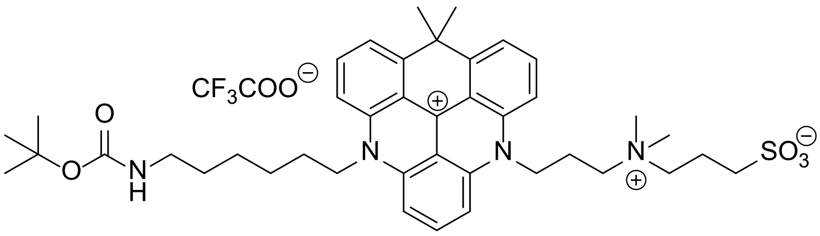

4-(6-((tert-butoxycarbonyl)amino)hexyl)-12,12-dimethyl-8-(2-morpholinoethyl)-8,12-dihydro-4H-benzo[1,8]isoquinolino[3,4,5,6-klmn]acridin-3a2-ylium trifluoroacetate (compound **8f**).

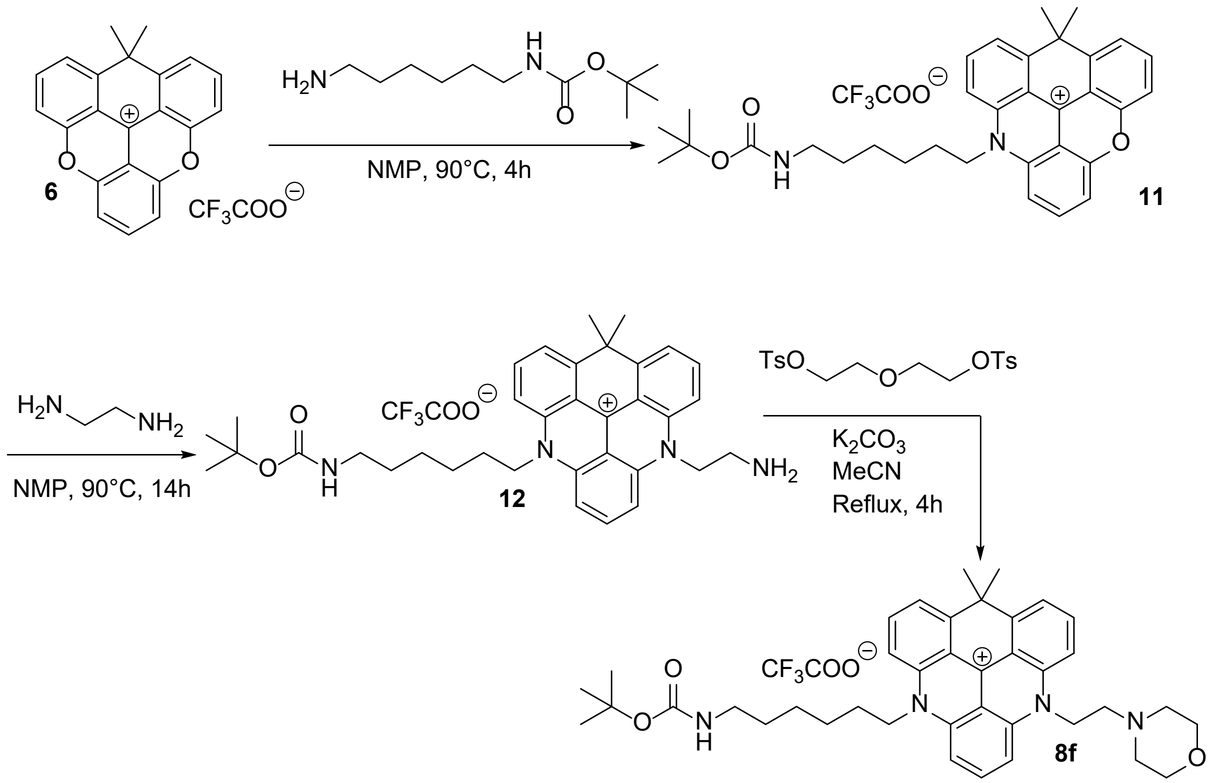

3-((3-(8-(6-((azido)hexyl)-12,12-dimethyl-8,12-dihydro-4H-benzo[1,8]isoquinolino[3,4,5,6-klmn]acridin-3a2-ylium-4-yl)propyl)dimethylammonio)propane-1-sulfonate trifluoroacetate (compound **8g**).

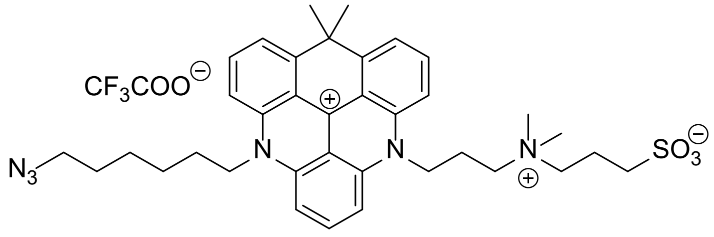

Peptide synthesis: Fmoc-L-Dab(NH_2)-OAll

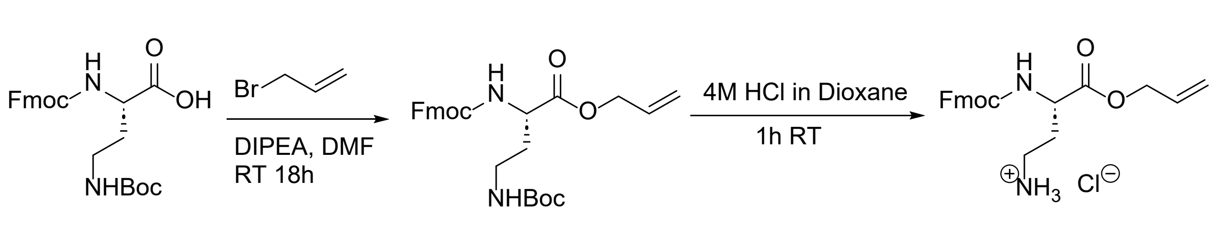

Supplementary Figures and Tables

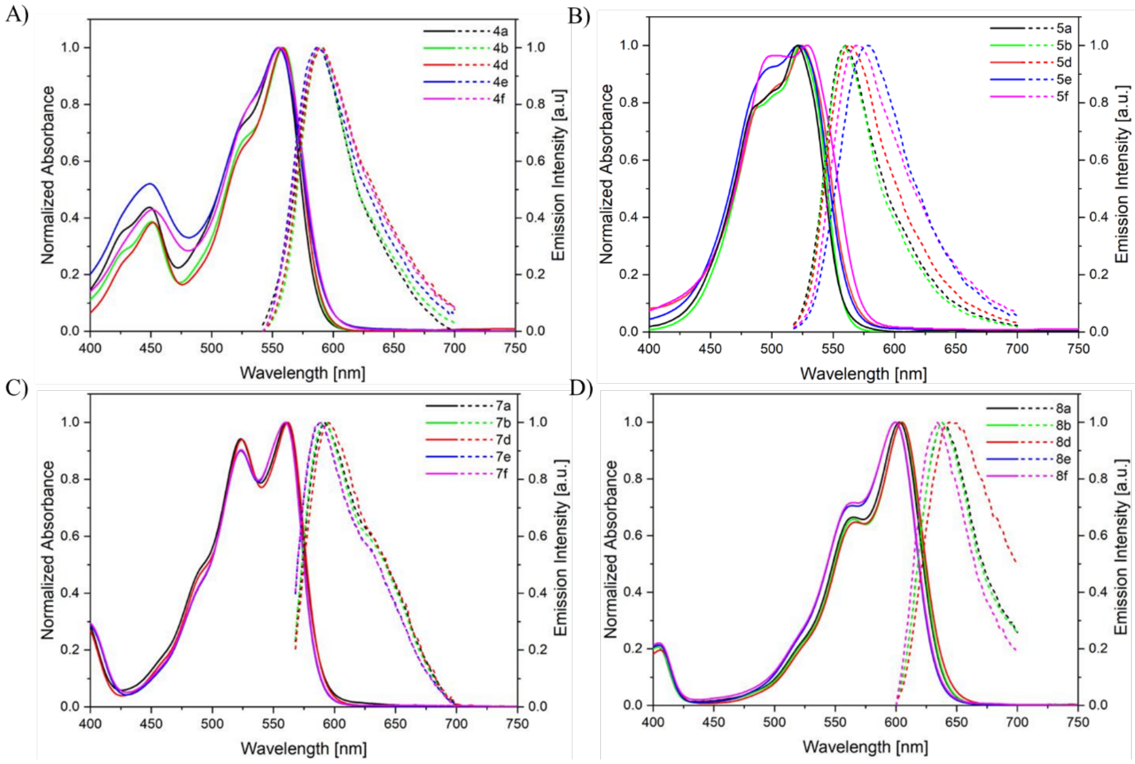

Supplementary Figure 1. Normalized absorbance and emission spectra of TA fluorophores. A) Absorbance (solid) and emission (dashed) spectra of compounds **4a-4f**: exc: 514 nm, emission: 544-700 nm. B) Absorbance (solid) and emission (dashed) spectra of compounds **5a-5f**: exc: 488 nm, emission: 518-700 nm. C) Absorbance (solid) and emission (dashed) spectra of compounds **7a-7f**: exc: 514 nm, emission: 544-700 nm. D) Absorbance and emission spectra of compounds **8a-8f**: exc: 570 nm, emission: 600-700 nm.

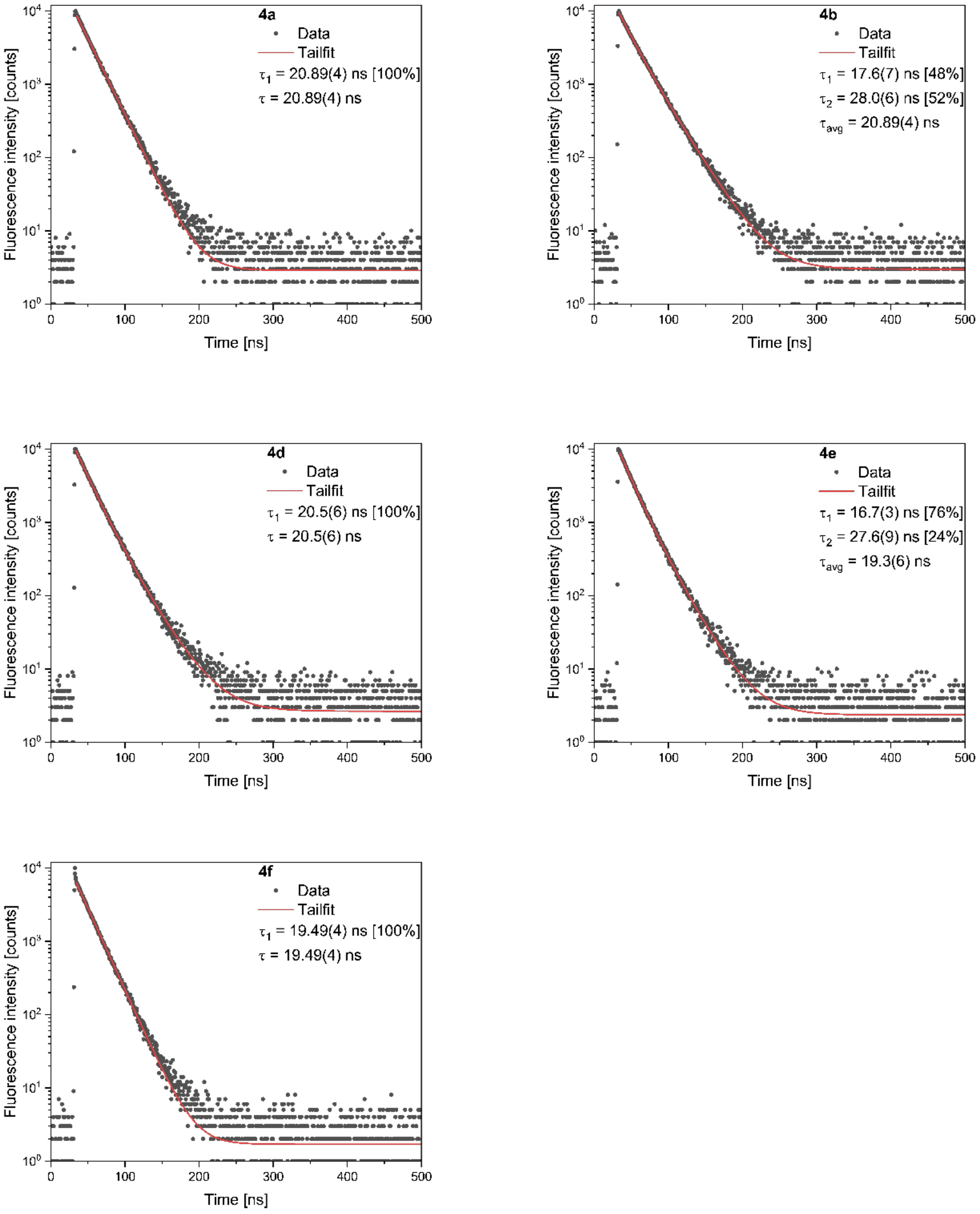

Supplementary Figure 2. Excited state fluorescence lifetime decays for the TA fluorophores 4a-4f. Excitation 450 nm with a repetition rate of 2.5 MHz, emission at individual emission maxima as listed in Table 1. A tail fit model was used to determine the lifetimes (τ).

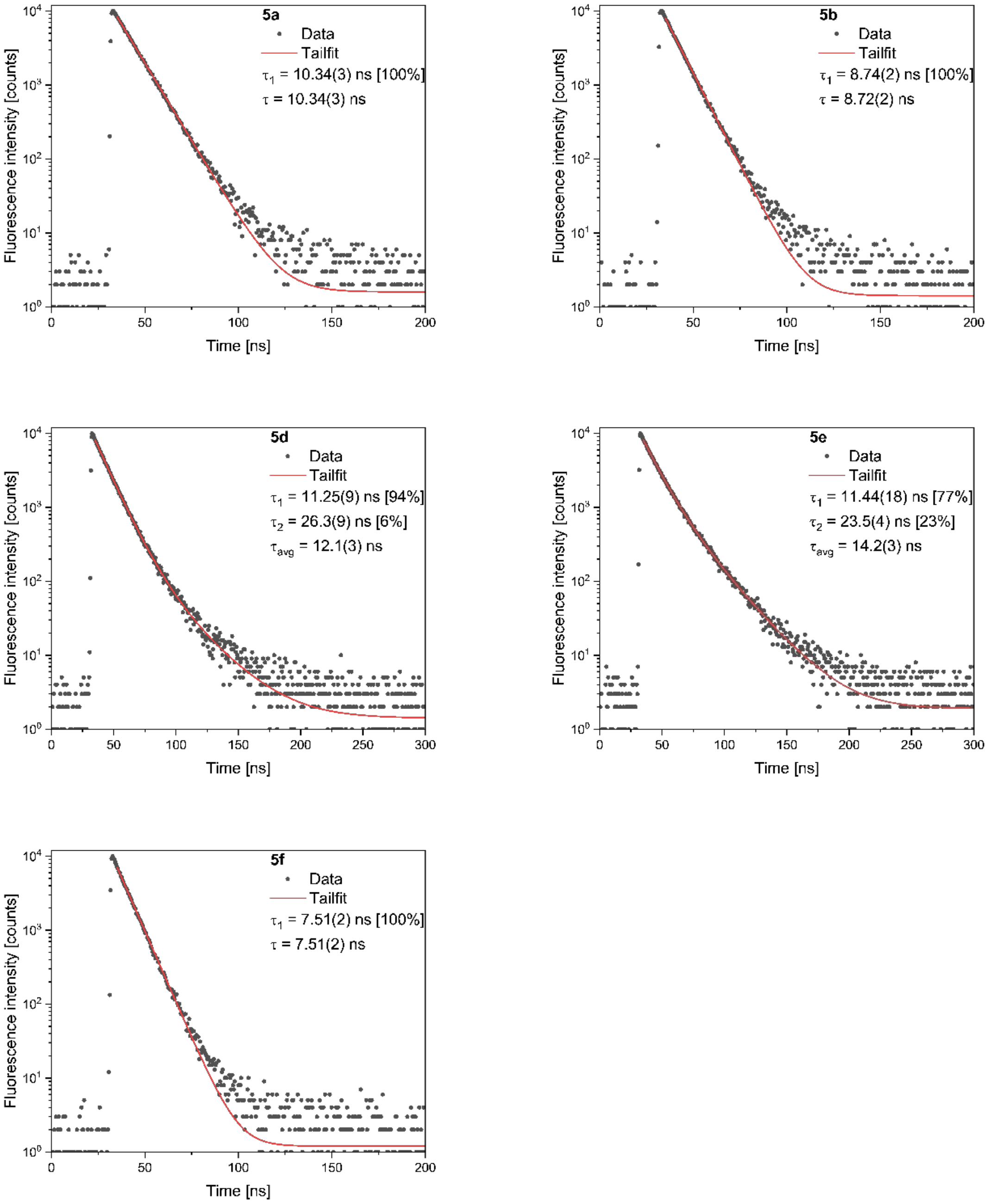

Supplementary Figure 3. Excited state fluorescence lifetime decays for the TA fluorophores 5a-5f. Excitation 450 nm with a repetition rate of 2.5 MHz, emission at individual emission maxima as listed in Table 1. A tail fit model was used to determine the lifetimes (τ).

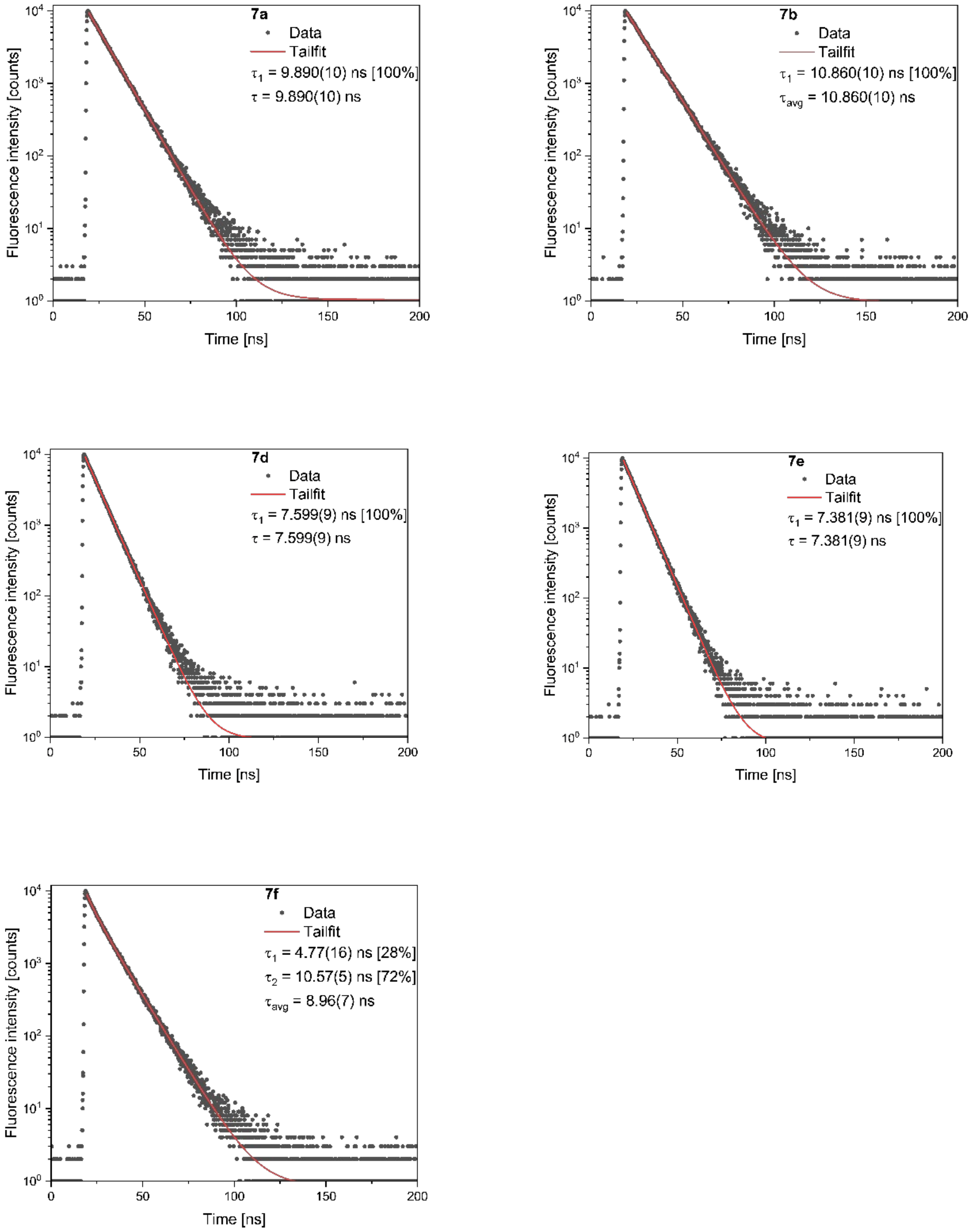

Supplementary Figure 4. Excited state fluorescence lifetime decays for the TA fluorophores 7a-7f. Excitation 450 nm with a repetition rate of 2.5 MHz, emission at individual emission maxima as listed in Table 1. A tail fit model was used to determine the lifetimes (τ).

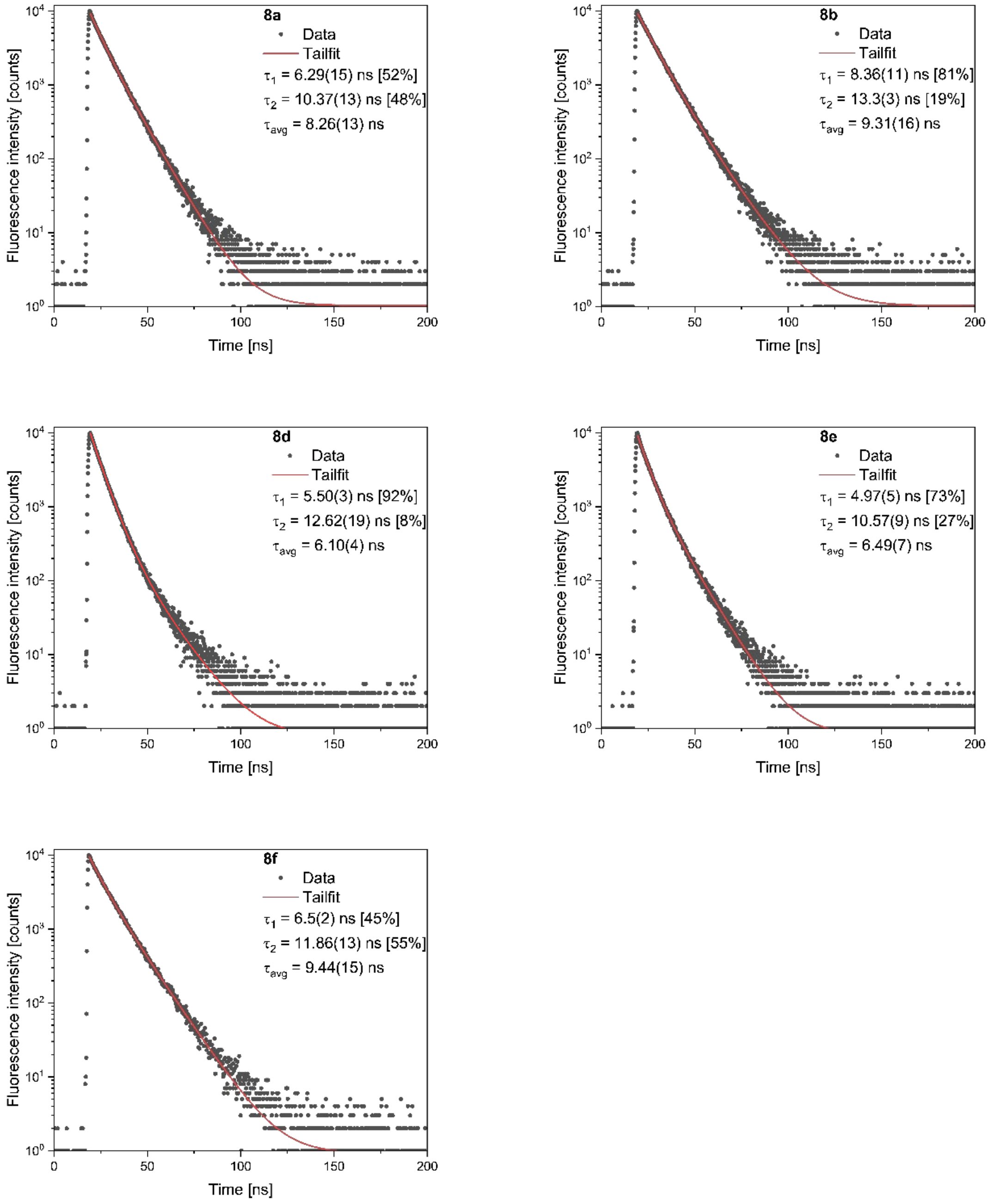

Supplementary Figure 5. Excited state fluorescence lifetime decays for the TA fluorophores 8a-8f. Excitation 450 nm with a repetition rate of 2.5 MHz, emission at individual emission maxima as listed in Table 1. A tail fit model was used to determine the lifetimes (τ).

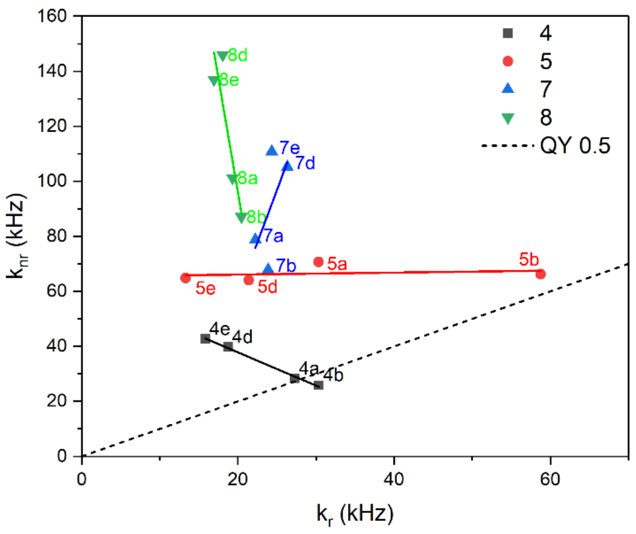

Supplementary Figure 6. Radiative decay rates (k_r) vs. non-radiative decay rates (k_{nr}) for the different families of TA fluorophores. Morpholine-containing TA fluorophores (compounds **4f**, **5f**, **7f**, **8f**) were omitted due to PeT quenching of the morpholine side chains. The dashed line indicates data for fluorescence quantum yields of 0.5.

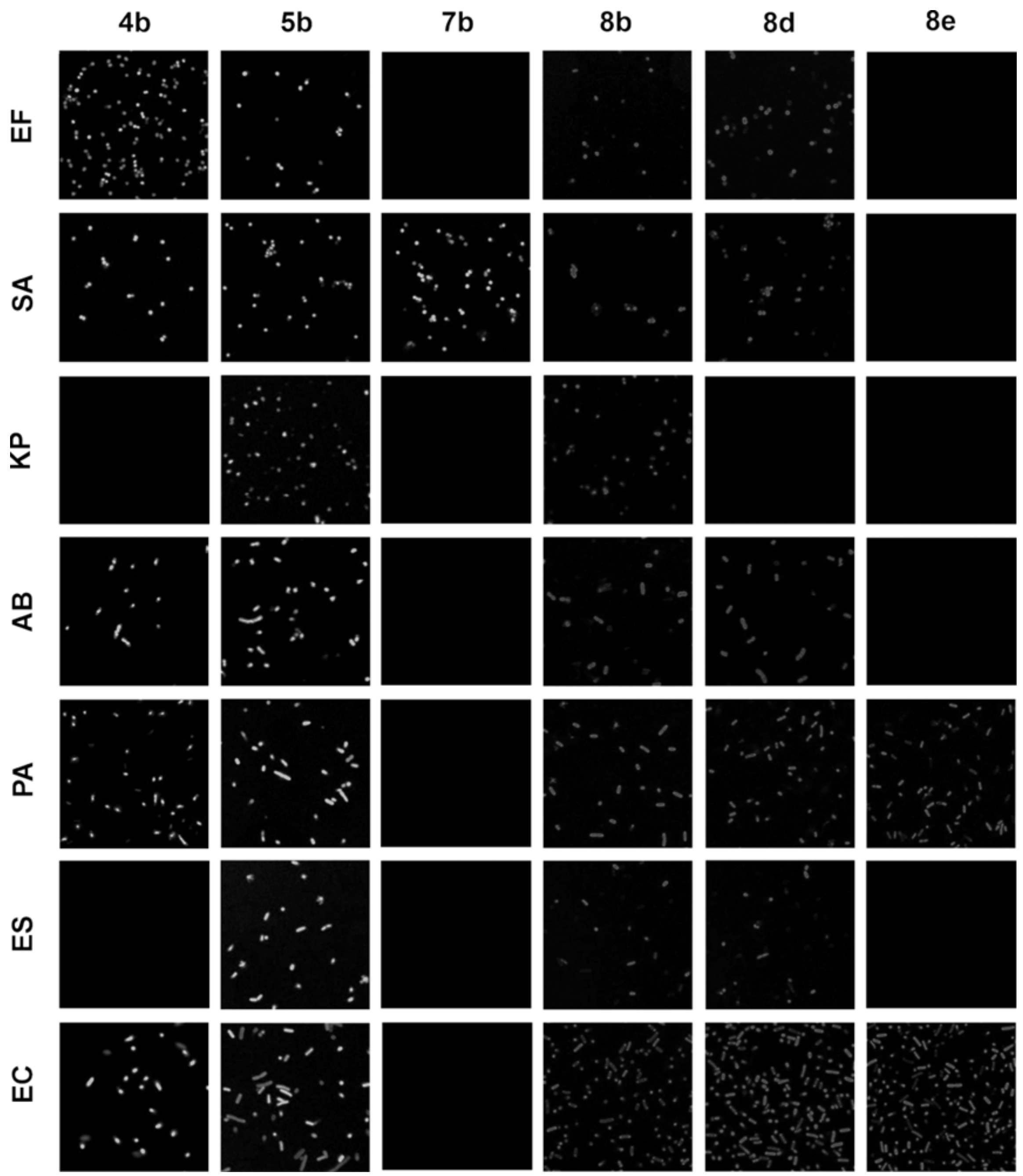

Supplementary Figure 7. Representative fluorescence intensity microscopy images of ESKAPEE bacterial species after incubation with TA fluorophores. Excitation/emission: **4b** (530/550-700 nm), **7b** (523/543-700 nm), **5b** (488/508-650 nm), **8b**, **8d** and **8e** (580/600-800 nm). Concentration of TA fluorophores: **4b**, **5b** and **7b** (5 μM); **8b**, **8d** and **8e** (10 μM). Scale bar: 5 μm. EF: *E. faecium*; SA: *S. aureus*; KP: *K. pneumoniae*; AB: *A. baumannii*; PA: *P. aeruginosa*; ES: *E. cloacae*; EC: *E. coli*.

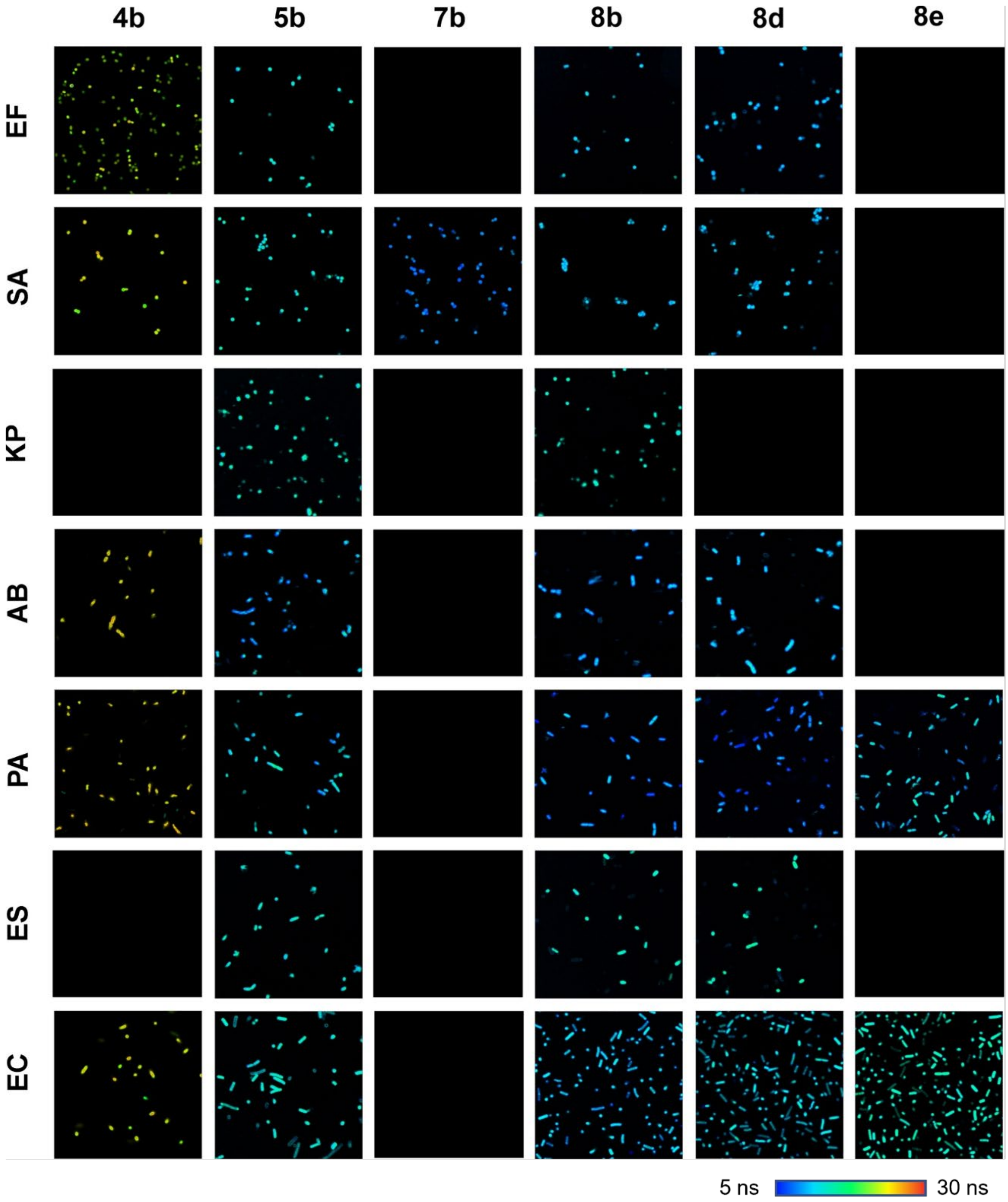

Supplementary Figure 8. Representative pseudocolored FLIM images of ESKAPEE bacterial species after incubation with TA fluorophores. Excitation/emission: **4b** (530/550-700 nm), **7b** (523/543-700 nm), **5b** (488/508-650 nm), **8b**, **8d** and **8e** (580/600-800 nm). Concentrations: **4b**, **5b** and **7b** (5 μM); **8b**, **8d** and **8e** (10 μM). Scale bar: 5 μm. EF: *E. faecium*; SA: *S. aureus*; KP: *K. pneumoniae*; AB: *A. baumannii*; PA: *P. aeruginosa*; ES: *E. cloacae*; EC: *E. coli*.

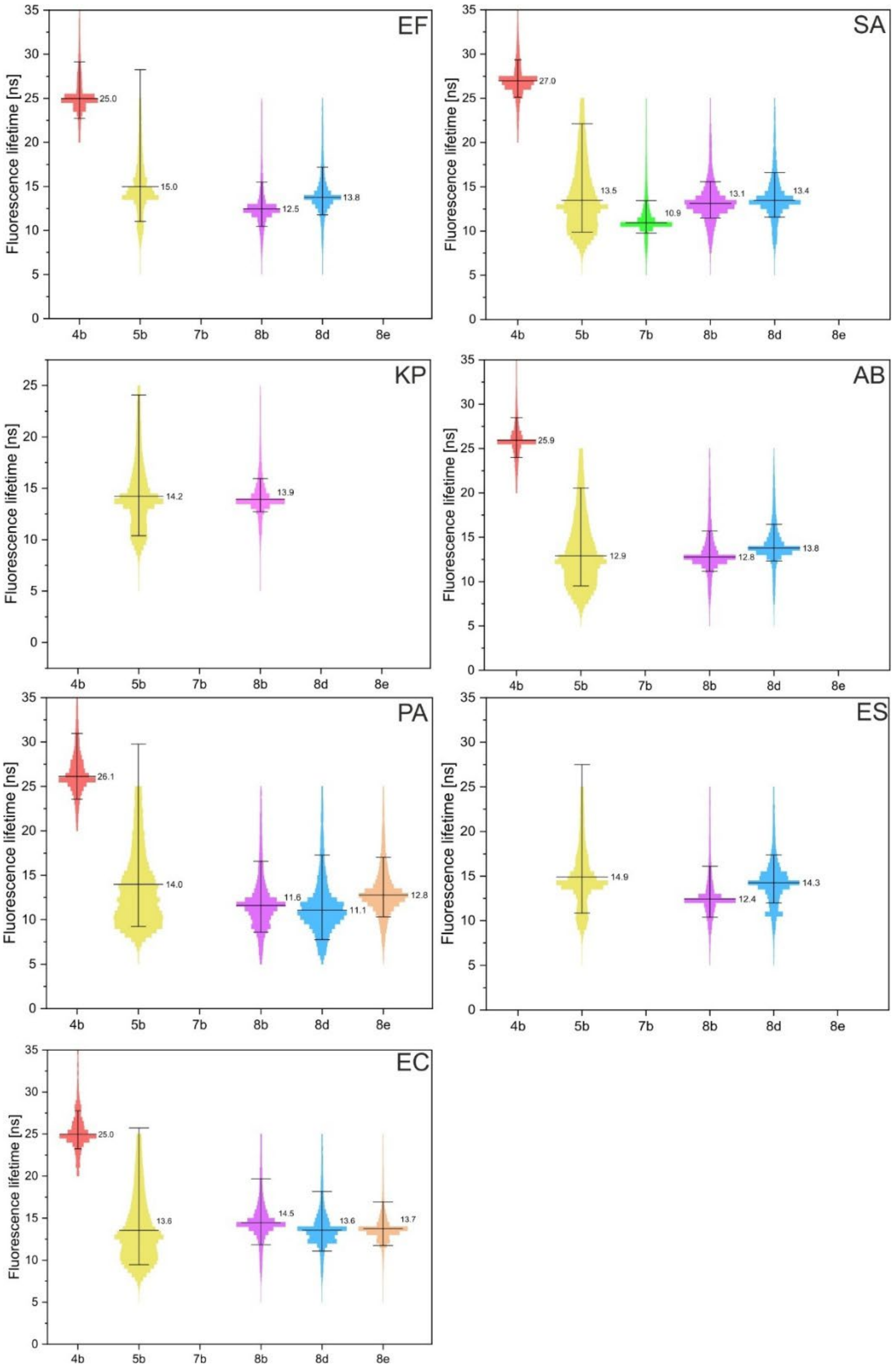
EF
SA
KP
AB
PA
ES
EC
Fluorescence lifetime [ns]
4b
5b
7b
8b
8d
8e
25.0
15.0
12.5
13.8
27.0
13.5
10.9
13.1
13.4
14.2
13.9
25.9
12.9
12.8
13.8
26.1
14.0
11.6
11.1
12.8
14.9
12.4
14.3
25.0
13.6
14.5
13.6
13.7

Supplementary Figure 9. Distribution of the fitted fluorescence lifetimes extracted from FLIM imaging for all ESKAPEE pathogens. Data points were binned into 0.2 ns bins prior to representation, with equal widths. Median values and 5-95% confidence intervals are presented from 10 independent measurements of at least 2 independent biological replicates. EF: *E. faecium*; SA: *S. aureus*; KP: *K. pneumoniae*; AB: *A. baumannii*; PA: *P. aeruginosa*; ES: *E. cloacae*; EC: *E. coli*.

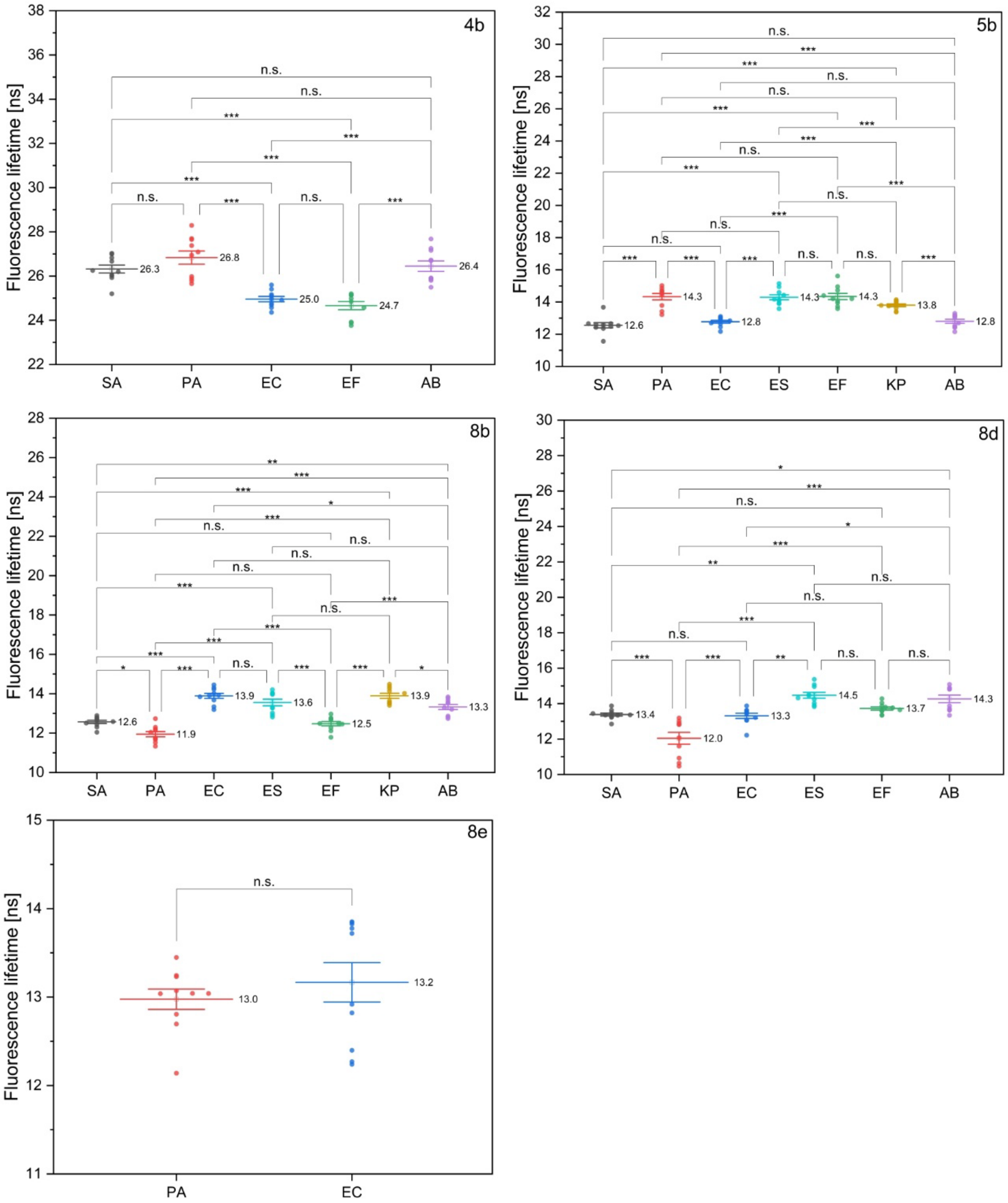

Supplementary Figure 10. Fluorescence lifetimes for TA fluorophores in ESKAPEE bacterial species. Values obtained from at least 10 measurements across independent biological replicates. EF: *E. faecium*; SA: *S. aureus*; KP: *K. pneumoniae*; AB: *A. baumannii*; PA: *P. aeruginosa*; ES: *E. cloacae*; EC: *E. coli*. Statistical analysis was performed by ANOVA (* for $p<0.05$, ** for $p<0.01$, *** for $p<0.001$, **** for $p<0.0001$).

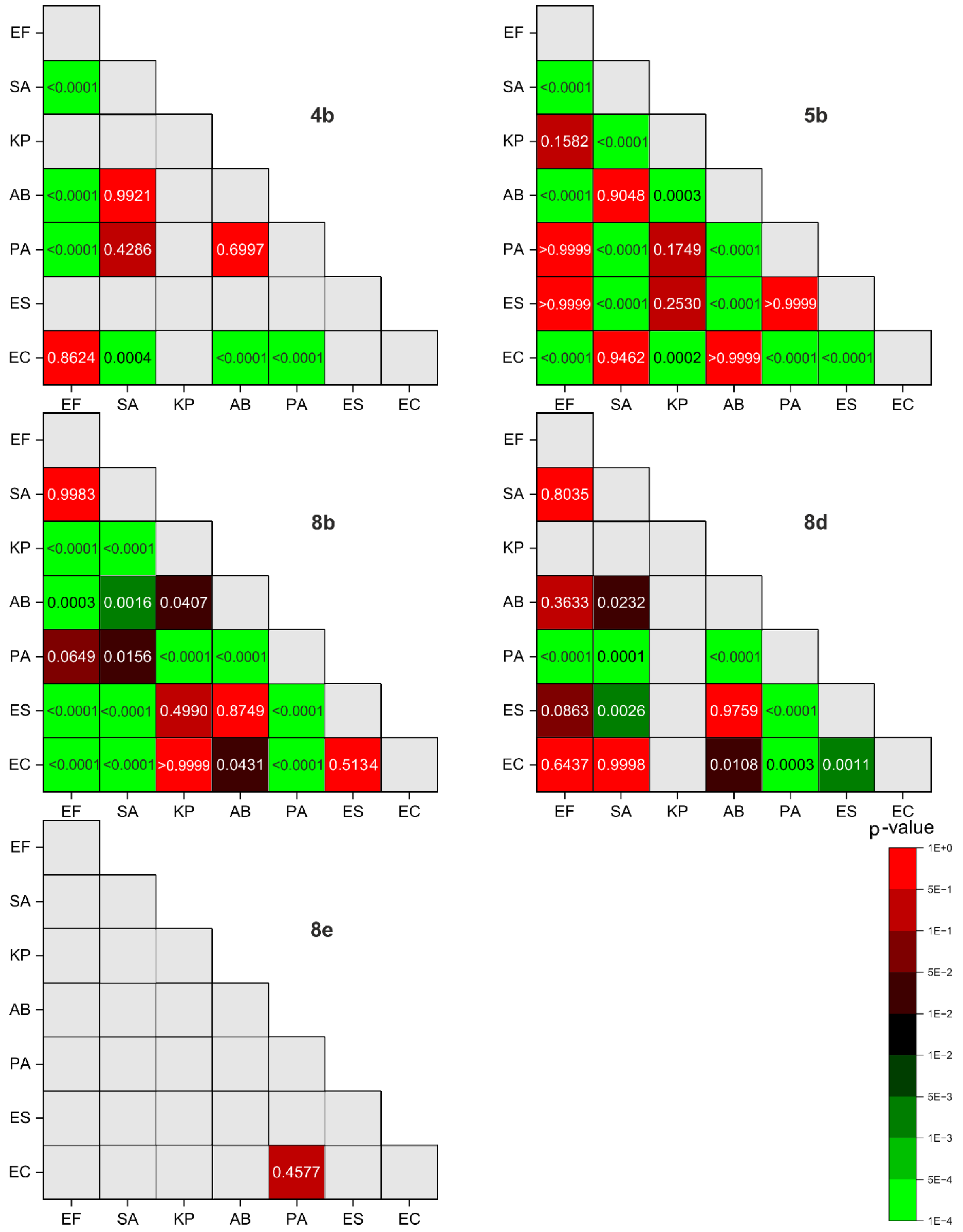

Supplementary Figure 11. Summarized statistical analysis for TA fluorophores in ESKAPEE bacterial species. Pseudocolored heatmaps including all p values from the ANOVA statistical analysis of fluorescence lifetimes presented in Supplementary Figure 10.

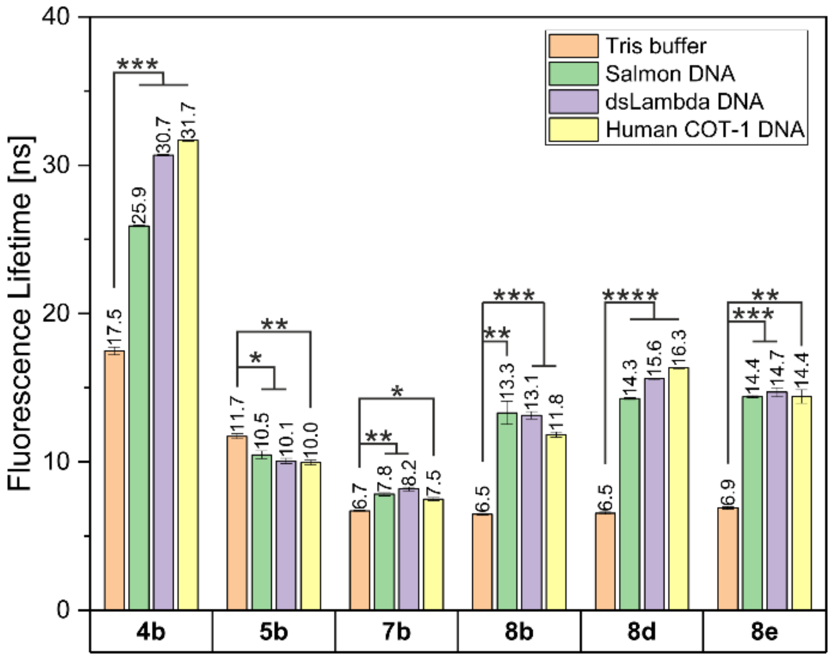

Supplementary Figure 12. TA fluorophores show increased fluorescence lifetimes after binding to DNA. All TA fluorophores (5 μM) were incubated in Tris buffer (10 mM, pH 7.0) without (orange) or with 100 μg mL^{-1} of different available DNA sources (green: salmon DNA; purple: dsLambda DNA; yellow: human COT-1 DNA). Values are presented as means ± SEM (n=3). Statistical analysis between free and DNA-bound fluorophores was performed by ANOVA (* for $p<0.05$, ** for $p<0.01$, *** for $p<0.001$, **** for $p<0.0001$).

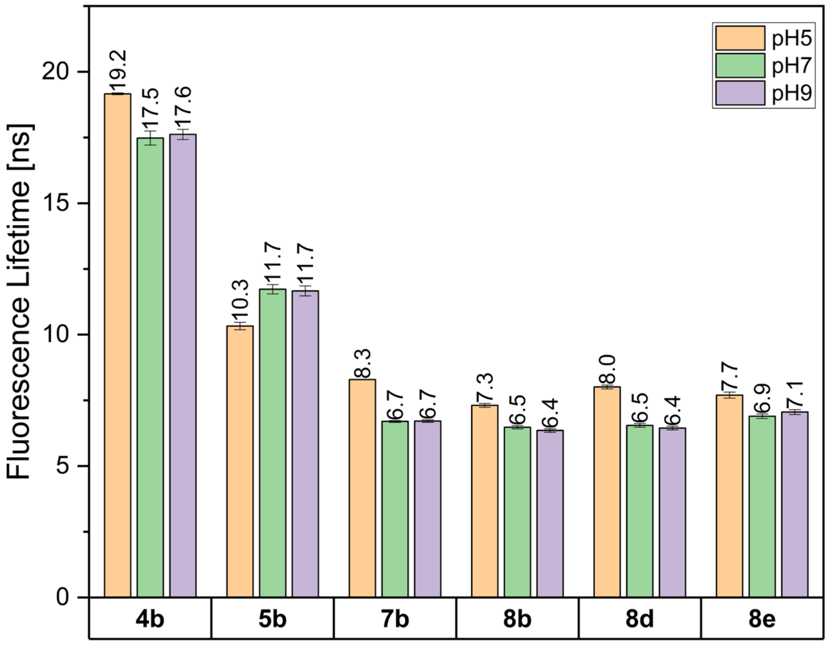

Supplementary Figure 13. Fluorescence lifetimes of TA fluorophores in different pH environments. All TA fluorophores (5 µM) were dissolved in acetate (10 mM, pH 5.0, orange), Tris (10 mM, pH 7.0, green) or carbonate (10 mM, pH 9.0, purple) buffer. Values are presented as means ± SEM (n=3).

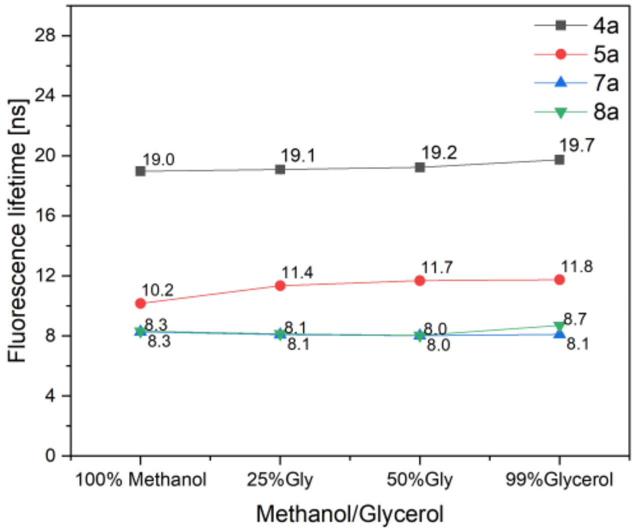

Supplementary Figure 14. Fluorescence lifetimes of TA fluorophores in environments of different viscosity. All compounds (5 µM) were dissolved in mixtures of methanol and glycerol and lifetimes were recorded at r.t.

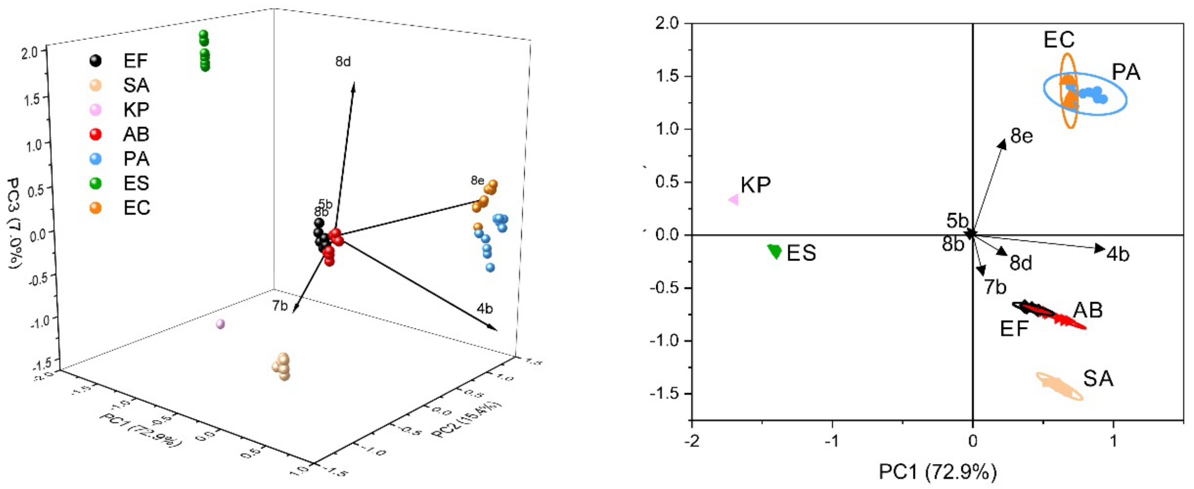

Supplementary Figure 15. PCA analysis of TA fluorophores after incubation with ESKAPEE bacterial species. Left) 3D canonical score and loading plots of the three-component PCA analysis. Right) 2D projection along the PC1-PC2 axes, showing all ESKAPEE species. The median values of 10 fields of view across multiple repeats were used to generate the reduced space. For the 2D plot, 95% confidence intervals were calculated and plotted as ellipses. EF: *E. faecium*; SA: *S. aureus*; KP: *K. pneumoniae*; AB: *A. baumannii*; PA: *P. aeruginosa*; ES: *E. cloacae*; EC: *E. coli*.

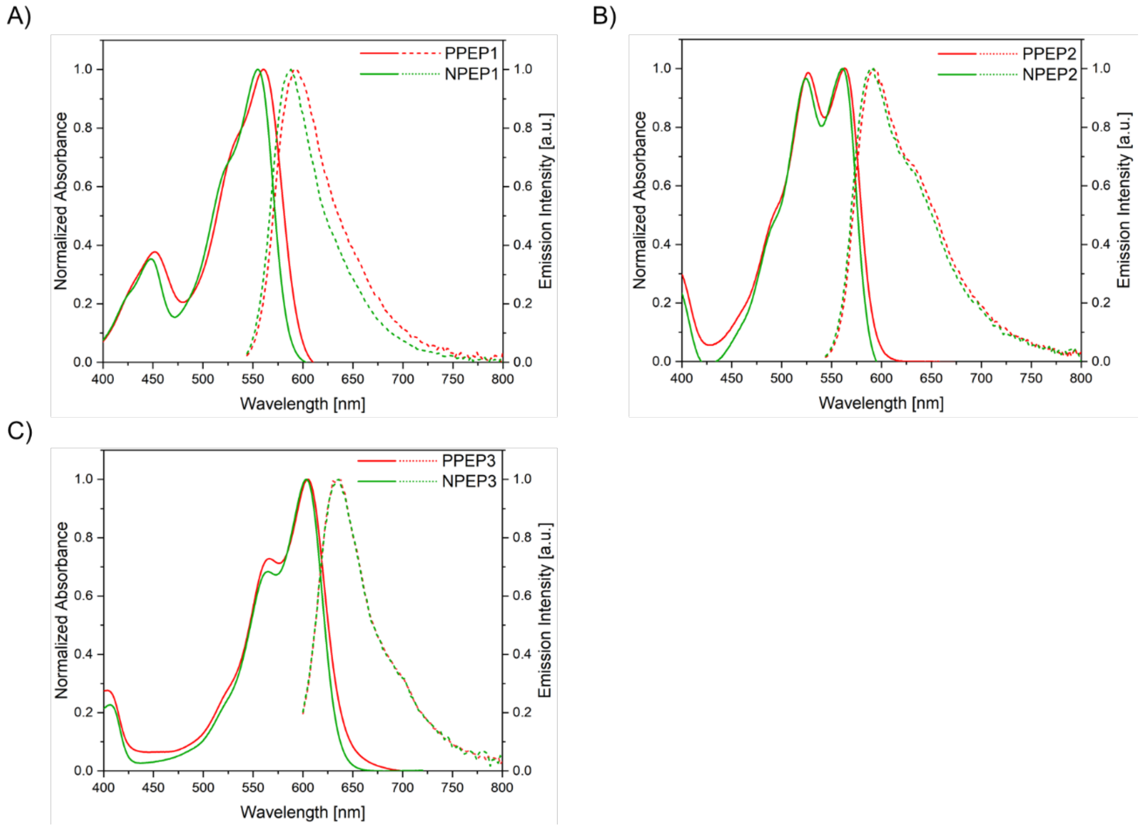

Supplementary Figure 16. Normalized absorbance and emission spectra of TA-peptides. A) Absorbance (solid) and emission (dashed) spectra of peptides **PPEP1** and **NPEP1**: exc: 514 nm, emission: 544-700 nm. B) Absorbance (solid) and emission (dashed) spectra of peptides **PPEP2** and **NPEP2:** exc: 514 nm, emission: 544-700 nm. C) Absorbance (solid) and emission (dashed) spectra of peptides **PPEP3** and **NPEP3:** exc: 570 nm, emission: 600-700 nm.

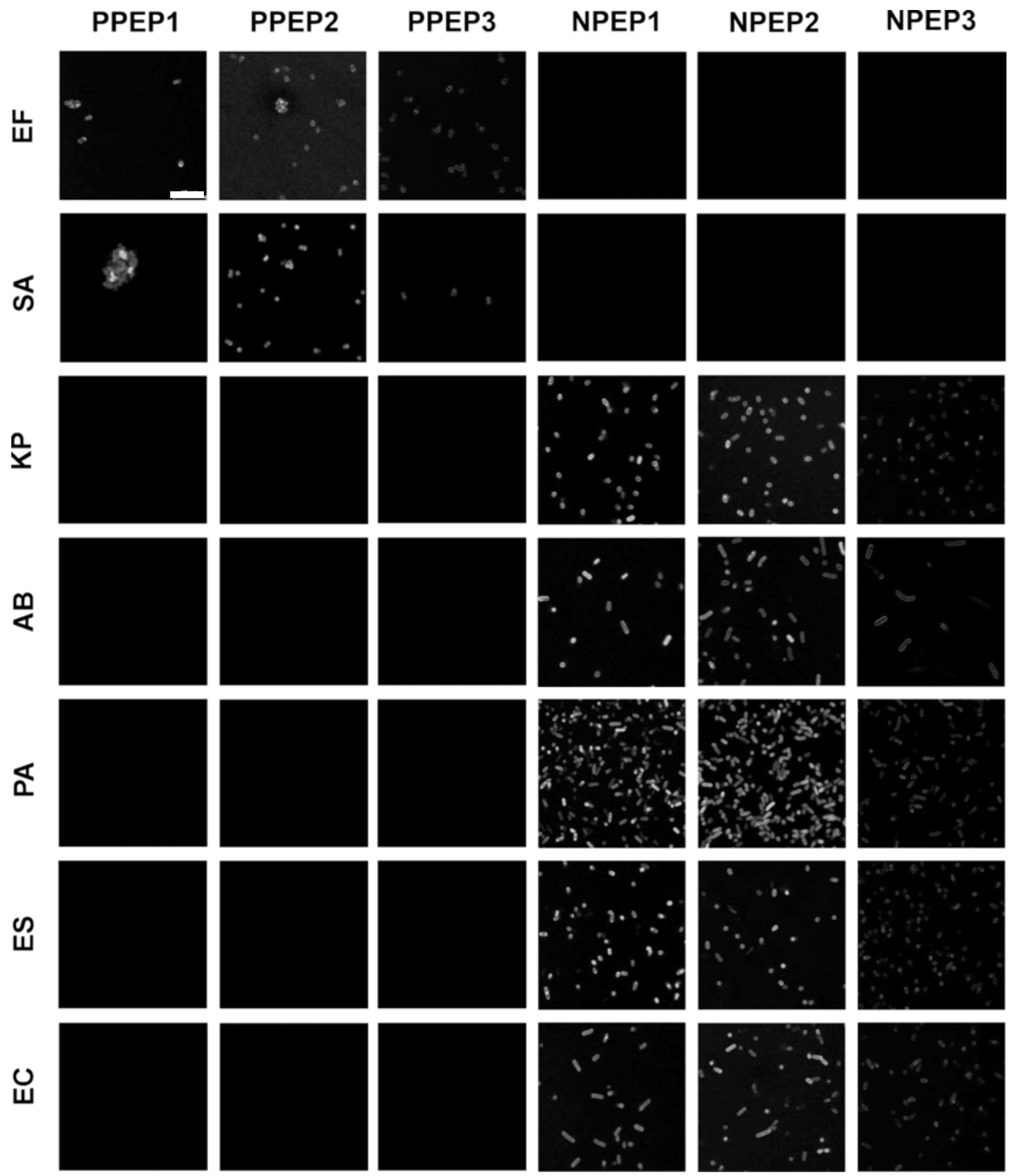

Supplementary Figure 17. Representative fluorescence intensity microscopy images of ESKAPEE bacterial species after incubation with TA-peptides. Excitation/emission: **PPEP1** and **NPEP1** (530/550-700 nm), **PPEP2** and **NPEP2** (523/543-700 nm), **PPEP3** and **NPEP3** (580/600-800 nm). Concentration for all TA-peptides: 2.5 µM. Scale bar: 5 µm. EF: *E. faecium*; SA: *S. aureus*; KP: *K. pneumoniae*; AB: *A. baumannii*; PA: *P. aeruginosa*; ES: *E. cloacae*; EC: *E. coli*.

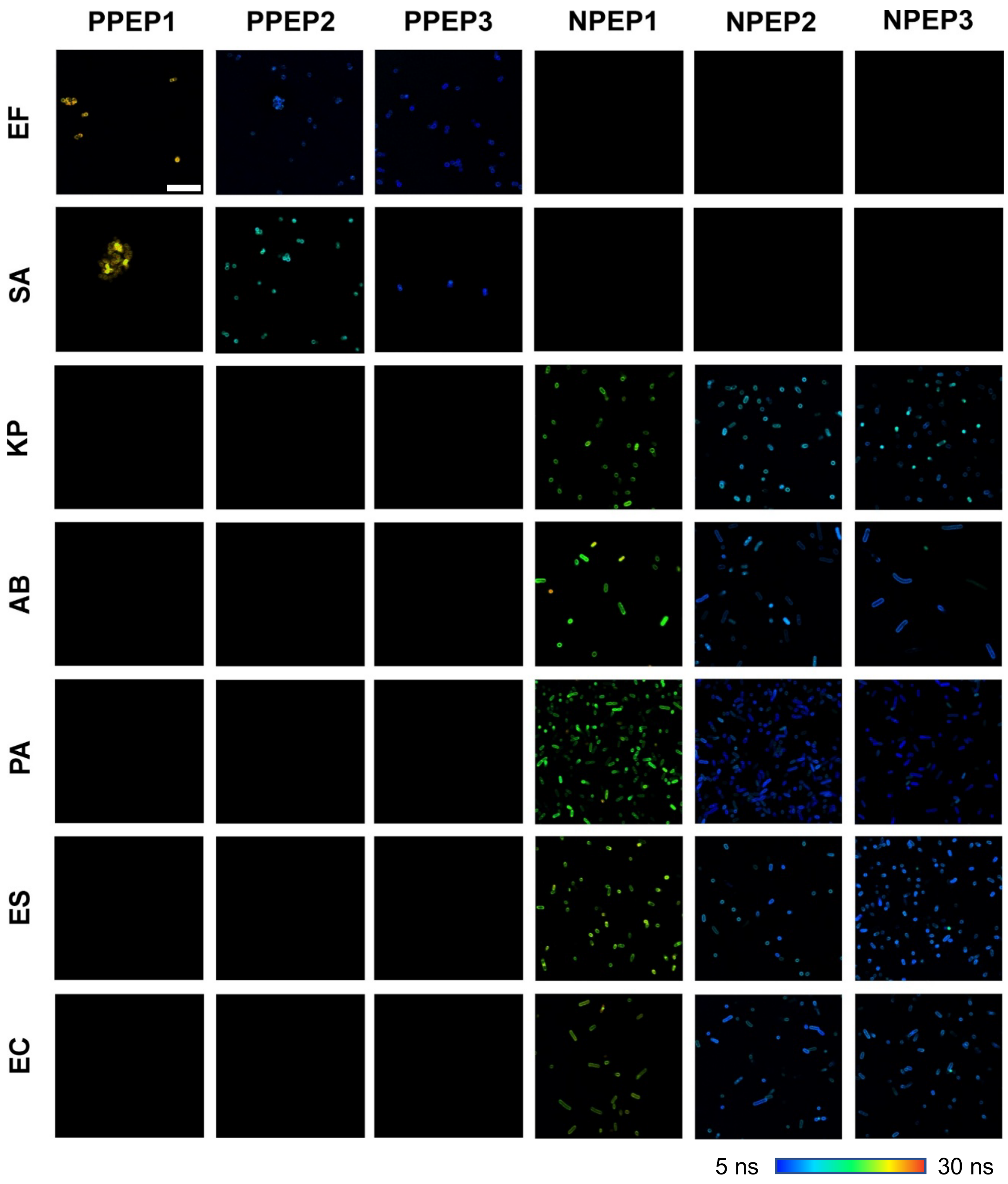

Supplementary Figure 18. Representative FLIM images of ESKAPEE bacterial species after incubation with TA-peptides. Excitation/emission: **PPEP1** and **NPEP1** (530/550-700 nm), **PPEP2** and **NPEP2** (523/543-700 nm), **PPEP3** and **NPEP3** (580/600-800 nm). Concentration for all TA-peptides: 2.5 μM. Scale bar: 5 μm. EF: *E. faecium*; SA: *S. aureus*; KP: *K. pneumoniae*; AB: *A. baumannii*; PA: *P. aeruginosa*; ES: *E. cloacae*; EC: *E. coli*.

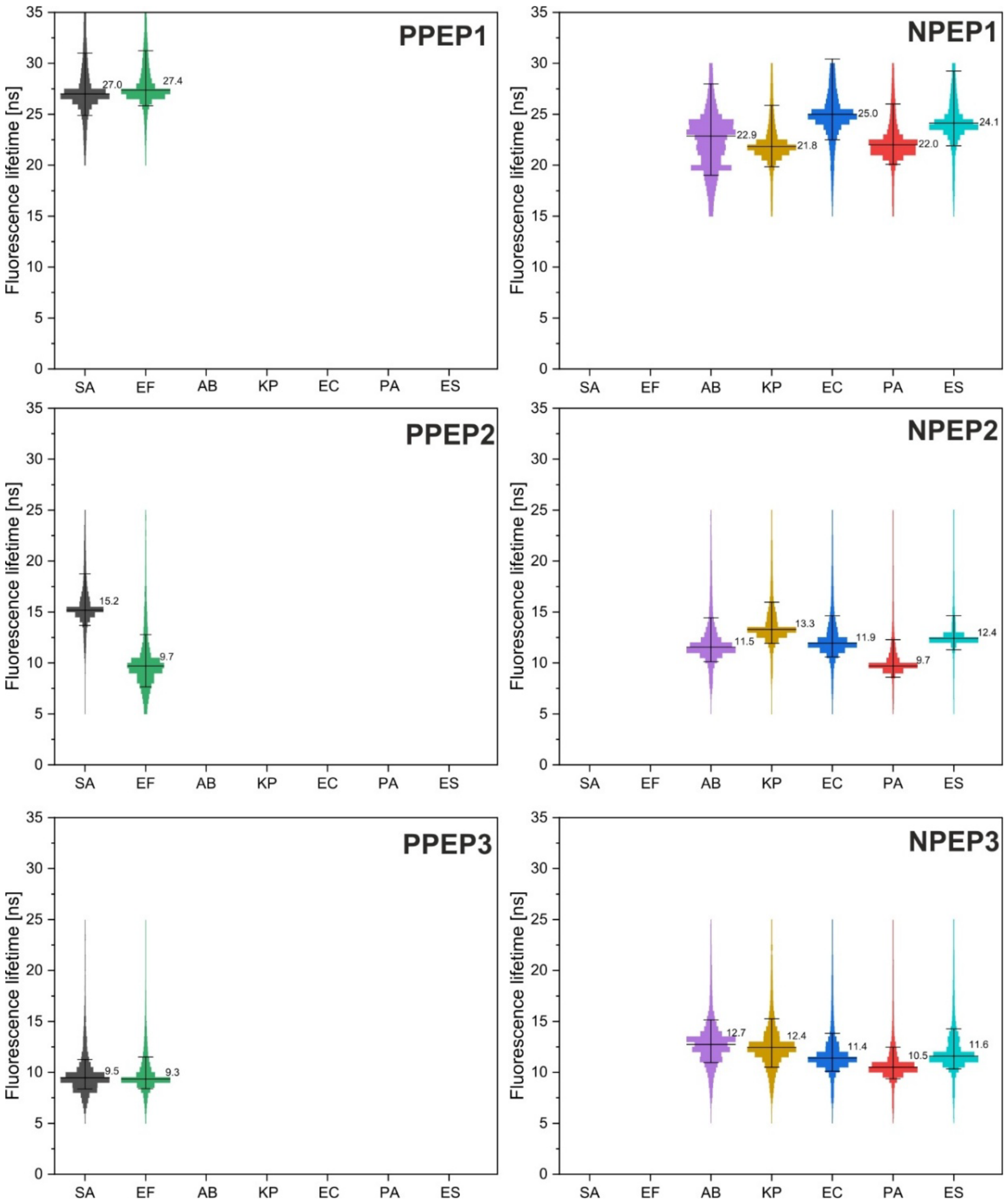

Supplementary Figure 19. Fluorescence lifetimes of TA-peptides in ESKAPEE bacterial species. Data points were binned into 0.2 ns bins prior to representation, with equal widths. Median values and 5-95% confidence intervals are presented from 10 independent measurements of at least 2 independent biological replicates.EF: *E. faecium*; SA: *S. aureus*; KP: *K. pneumoniae*; AB: *A. baumannii*; PA: *P. aeruginosa*; ES: *E. cloacae*; EC: *E. coli*.

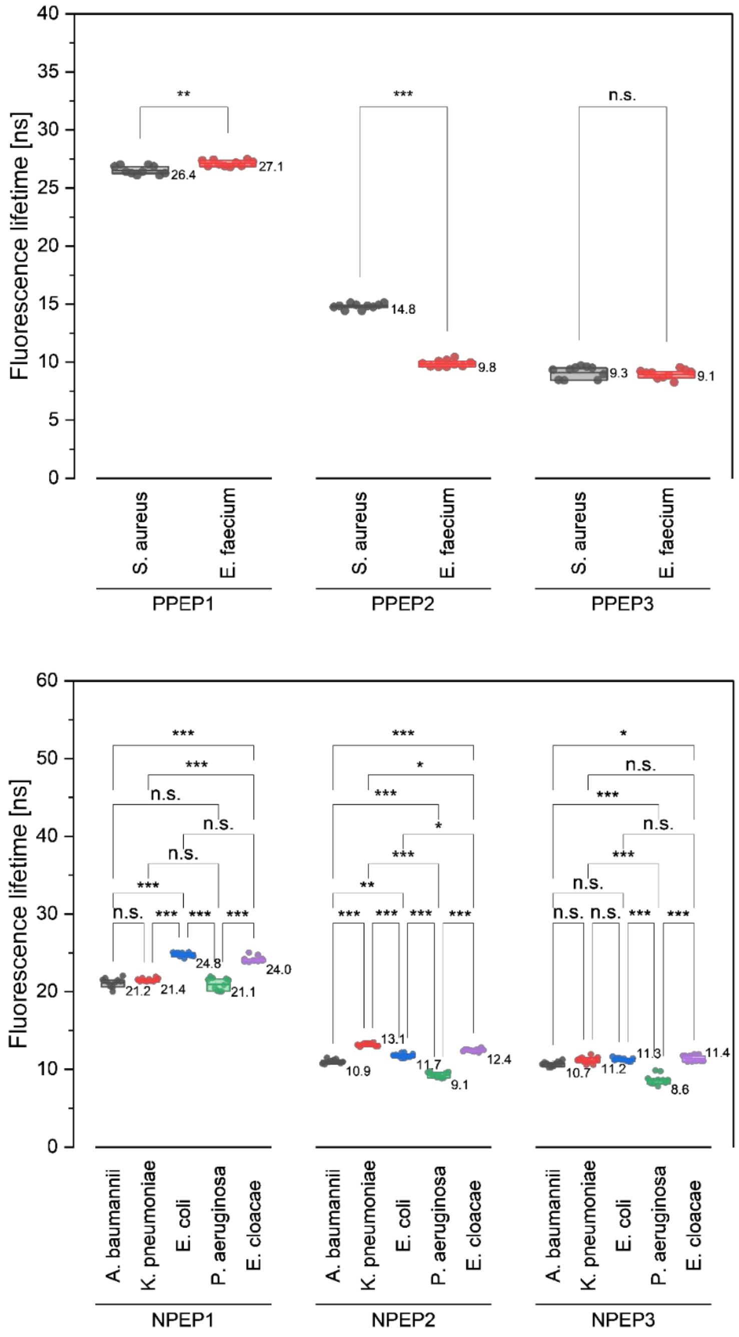

Supplementary Figure 20. Comparative analysis of fluorescence lifetimes for TA-peptides in different gram-positive and gram-negative bacteria. Top panel) **PPEP1**, **PPEP2** and **PPEP3** in gram-positive bacteria. Bottom panel) **NPEP1**, **NPEP2** and **NPEP3** in gram-negative bacteria. Values obtained from at least 10 measurements across independent biological replicates. Statistical analysis was performed by ANOVA (n.s. for p>0.05, * for p<0.05, ** for p<0.01, *** for p<0.001).

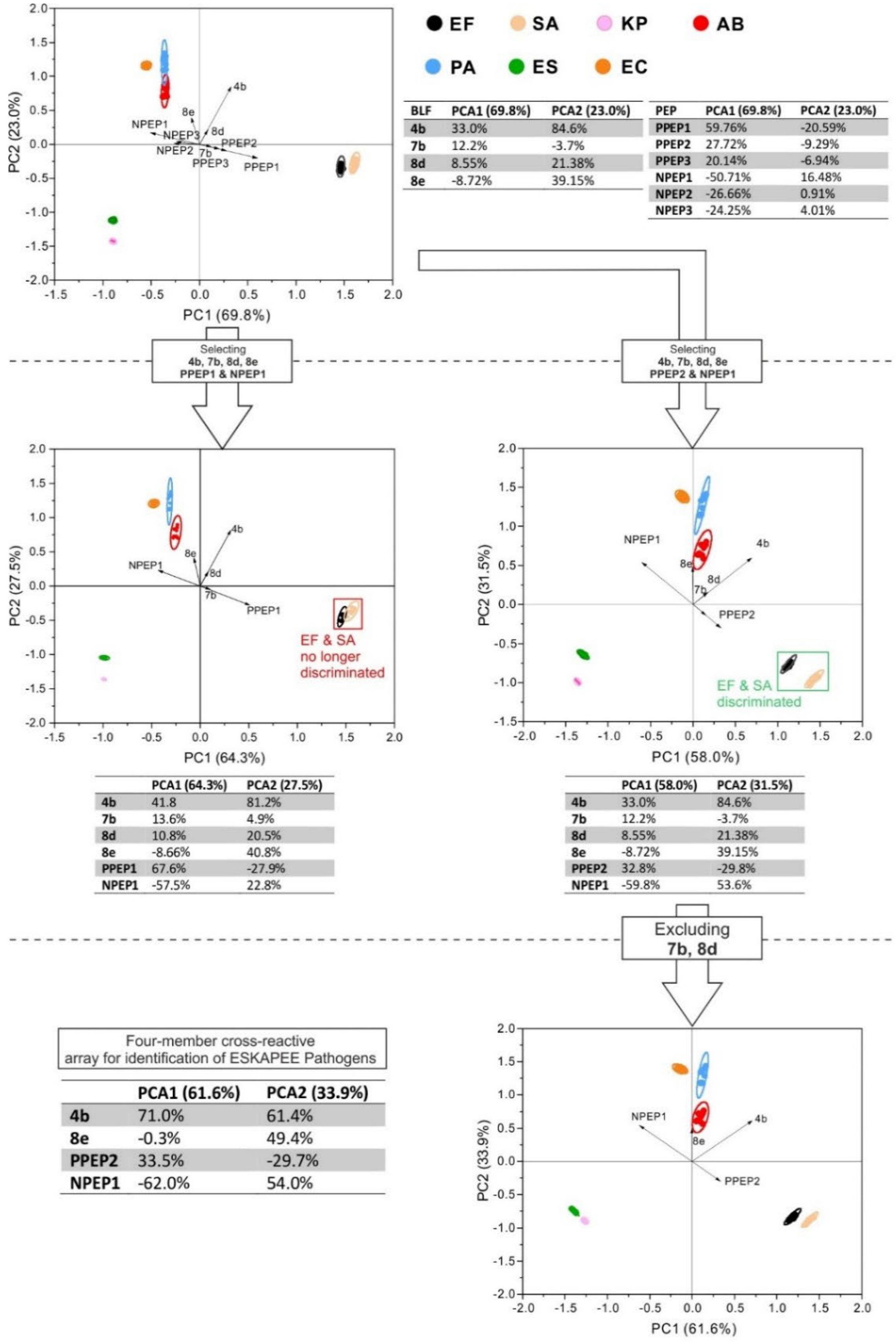

BLF	PCA1 (69.8%)	PCA2 (23.0%)
4b	33.0%	84.6%
7b	12.2%	-3.7%
8d	8.55%	21.38%
8e	-8.72%	39.15%

PEP	PCA1 (69.8%)	PCA2 (23.0%)
PPEP1	59.76%	-20.59%
PPEP2	27.72%	-9.29%
PPEP3	20.14%	-6.94%
NPEP1	-50.71%	16.48%
NPEP2	-26.66%	0.91%
NPEP3	-24.25%	4.01%

	PCA1 (64.3%)	PCA2 (27.5%)
4b	41.8	81.2%
7b	13.6%	4.9%
8d	10.8%	20.5%
8e	-8.66%	40.8%
PPEP1	67.6%	-27.9%
NPEP1	-57.5%	22.8%

	PCA1 (58.0%)	PCA2 (31.5%)
4b	33.0%	84.6%
7b	12.2%	-3.7%
8d	8.55%	21.38%
8e	-8.72%	39.15%
PPEP2	32.8%	-29.8%
NPEP1	-59.8%	53.6%

	PCA1 (61.6%)	PCA2 (33.9%)
4b	71.0%	61.4%
8e	-0.3%	49.4%
PPEP2	33.5%	-29.7%
NPEP1	-62.0%	54.0%

Supplementary Figure 21. Optimization of cross-reactive arrays combining TA fluorophores and TA-peptides. PCA analysis of several generations of arrays for the

identification of all ESKAPEE bacterial species. 2D projections along the PC1-PC2 axes show median values of 10 fields of view across multiple repeats. For the 2D plots, 95% confidence intervals were calculated and plotted as ellipses. EF: *E. faecium*; SA: *S. aureus*; KP: *K. pneumoniae*; AB: *A. baumannii*; PA: *P. aeruginosa*; ES: *E. cloacae*; EC: *E. coli*.

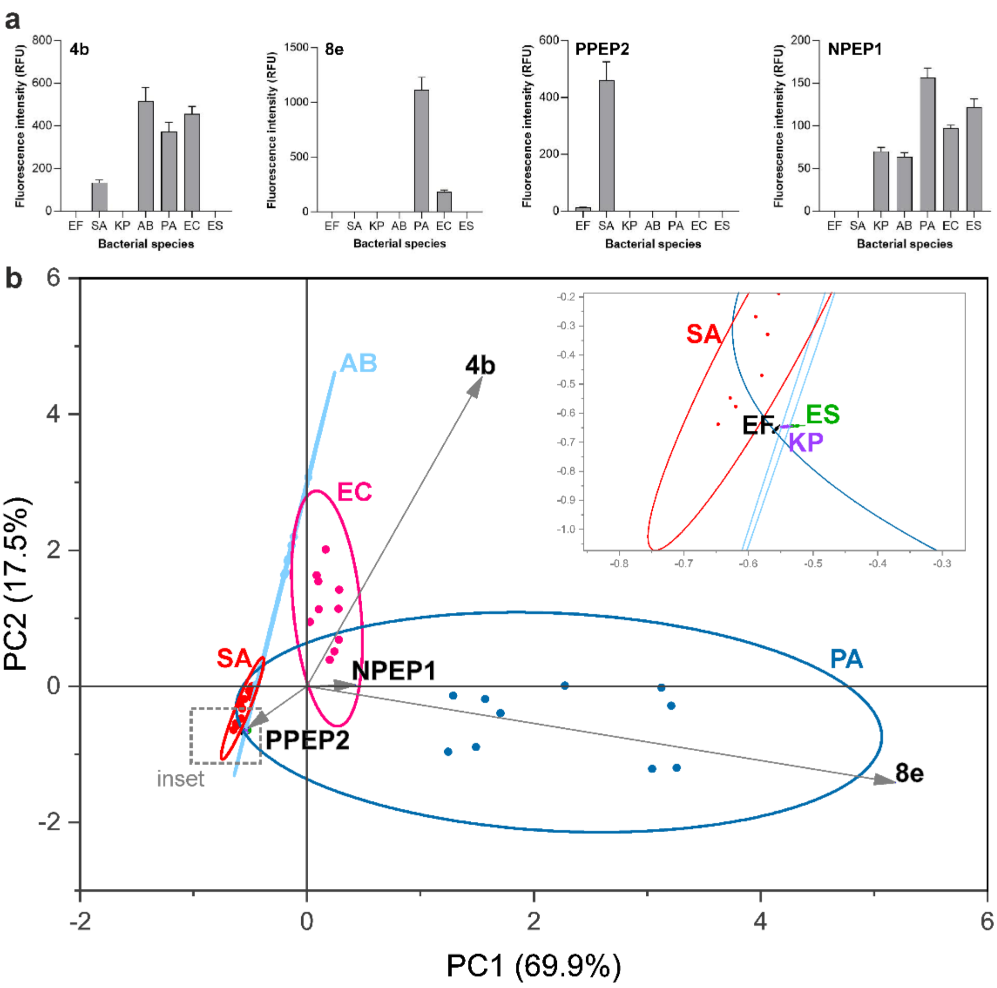

Supplementary Figure 22. Intensity-based analysis of ESKAPEE bacterial species using the optimal 4-member TA array. a) Fluorescence intensity data for TA fluorophores and TA-peptides in all ESKAPEE bacterial species. Values presented as means ± SEM from at least 10 measurements across independent biological replicates. b) PCA analysis (2 components) using fluorescence intensity data (shown in panel a) for failed discrimination of the ESKAPEE pathogens. Datapoints are individual experimental values from 10 measurements across independent biological replicates. 95% confidence intervals were calculated and plotted as ellipses. EF: *E. faecium*; SA: *S. aureus*; KP: *K. pneumoniae*; AB: *A. baumannii*; PA: *P. aeruginosa*; ES: *E. cloacae*; EC: *E. coli*.

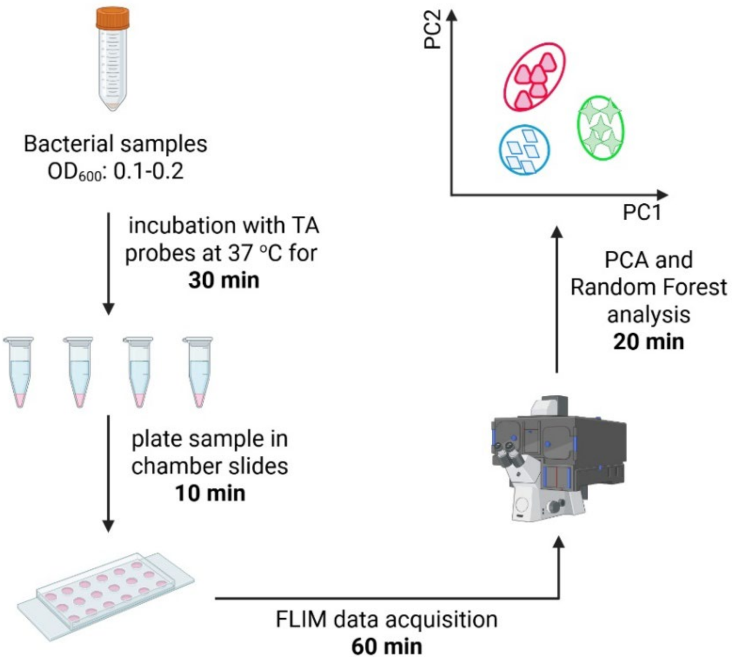

Supplementary Figure 23. Workflow for testing the TA-based cross-reactive lifetime array in bacterial samples. The workflow for the lifetime-based assignation of ESKAPEE bacterial species includes incubation with the TA-based cross-reactive array, lifetime acquisition and data analysis. PC: principal component.

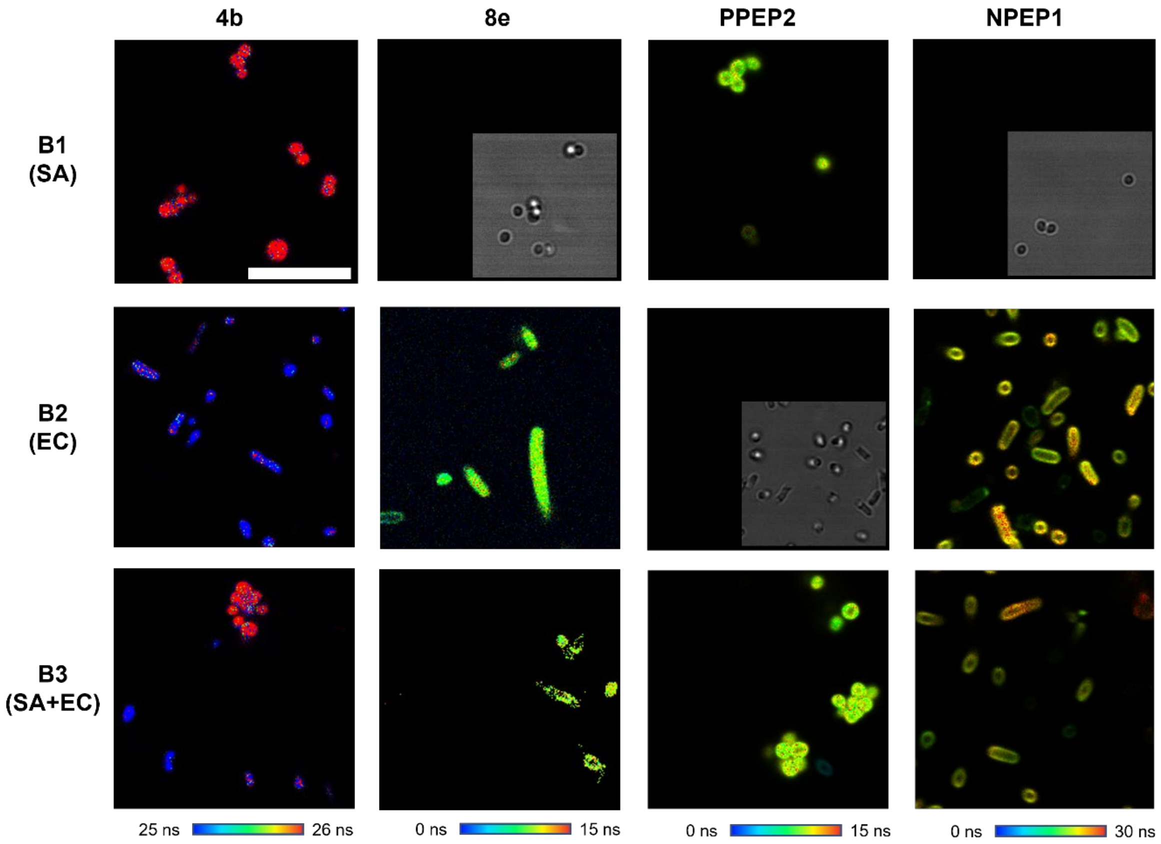

Supplementary Figure 24. Representative FLIM images of ESKAPEE bacterial species from spiked human blood samples after incubation with the optimal TA cross-reactive array. Excitation/emission: **4b** (530/550-700 nm), **8e** (580/600-800 nm), **PPEP2** (523/543-700 nm), **NPEP1** (530/550-700 nm). Brightfield microscopy images included to show bacteria when no fluorescence labeling was detected. Concentrations: **4b** (5 μM), **8e** (10 μM), **PPEP2** (2.5 μM), **NPEP1** (2.5 μM). Scale bar: 10 μm. SA: *S. aureus*; EC: *E. coli*.

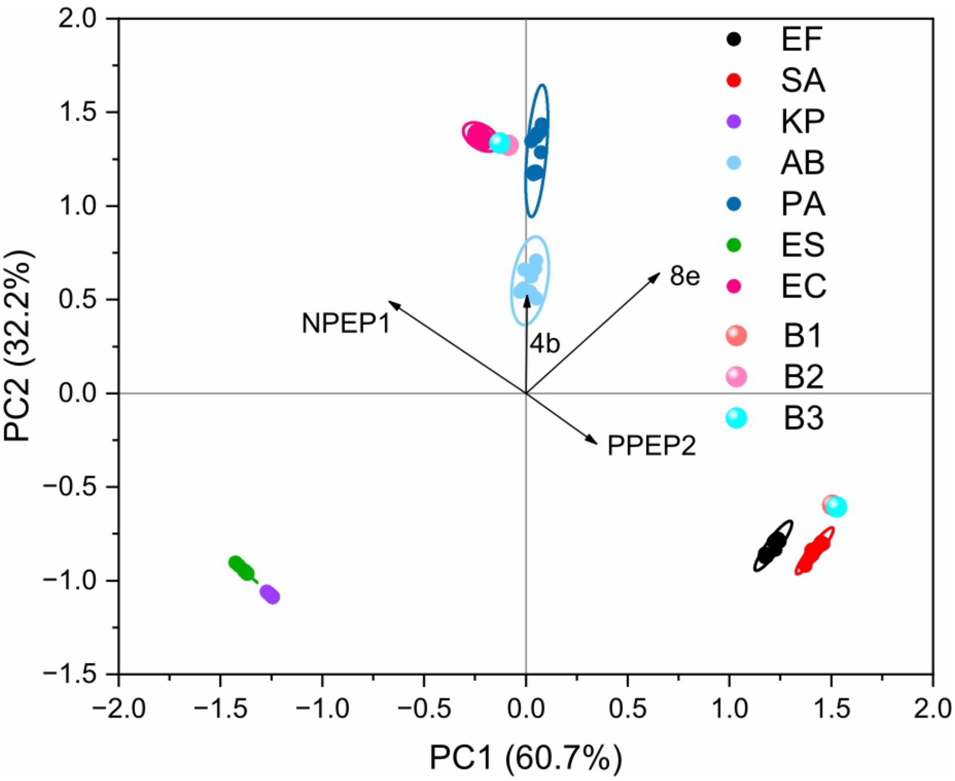

Supplementary Figure 25. PCA plots of the ESKAPEE bacterial panel (dots and solid confidence ellipses) and 3 spiked blood biosamples (B1: blood with SA, B2: blood with EC, B3: blood with SA and EC) using the optimal TA cross-reactive array (**4b**, **8e**, **PPEP1** and **NPEP2**). Training set: results of 10 independent images are plotted from multiple repeats with 95% confidence intervals. Blood biosamples: average results of 3 independent images are plotted from single experiment, no confidence interval reported. EF: *E. faecium*; SA: *S. aureus*; KP: *K. pneumoniae*; AB: *A. baumannii*; PA: *P. aeruginosa*; ES: *E. cloacae*; EC: *E. coli*.

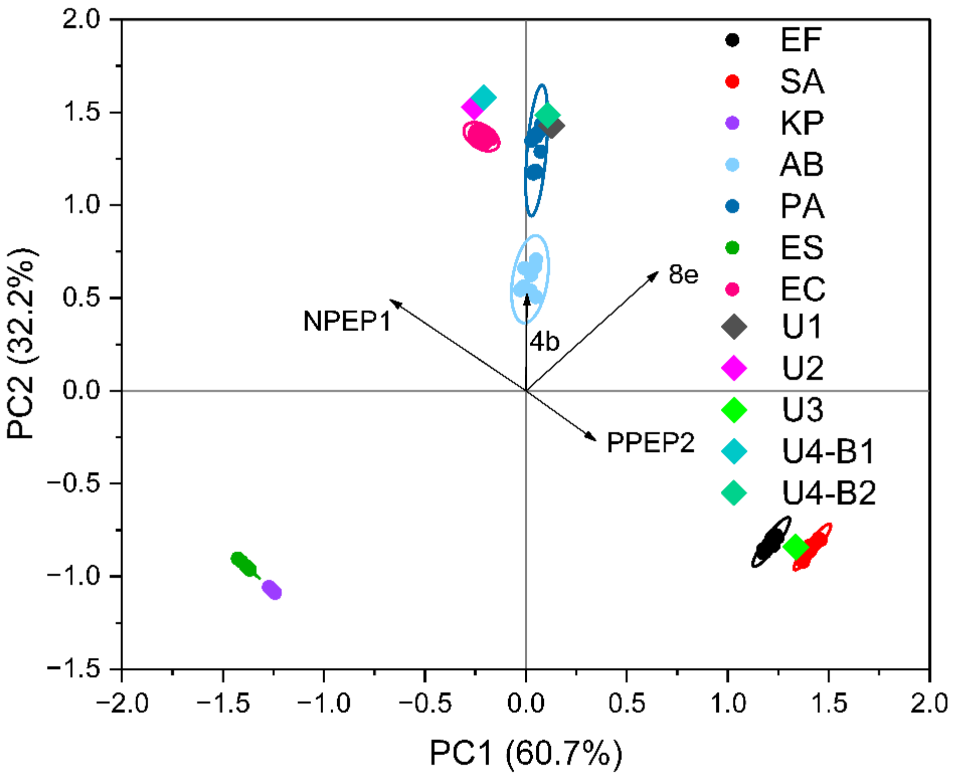

Supplementary Figure 26. PCA plots of the ESKAPEE bacterial panel (dots and solid confidence ellipses) and spiked urine samples (diamonds) using the optimised TA cross-reactive array (**4b**, **8e**, **PPEP1** and **NPEP2**). Training set: results of 10 independent images are plotted from multiple repeats with 95% confidence intervals. Urine biosamples: average results of 3 independent images are plotted from single experiment, no confidence interval reported. EF: *E. faecium*; SA: *S. aureus*; KP: *K. pneumoniae*; AB: *A. baumannii*; PA: *P. aeruginosa*; ES: *E. cloacae*; EC: *E. coli*. U1: urine sample with *P. aeruginosa;* U2: urine sample with *E. coli*; U3: urine sample with *S. aureus*; U4: urine sample with *P. aeruginosa* and *E. coli.*

Supplementary Table 1. Photophysical properties for TA-peptides. Values determined in water. Φ_{PL}: photoluminescence quantum yields; τ: fluorescence lifetimes.

TA-peptides	λ_{abs} (nm)	λ_{em} (nm)	τ (ns)	Φ_{PL}
**PPEP1**	561	593	22.5	0.39
**NPEP1**	555	588	17.5	0.28
**PPEP2**	564	594	8.6	0.26
**NPEP2**	562	590	7.0	0.25
**PPEP3**	606	636	9.2	0.14
**NPEP3**	603	636	7.4	0.15

Supplementary Table 2. ESKAPEE bacterial species and growth conditions in the training set. BHI: brain heart infusion; LB: lysogeny broth.

Bacterial species	Gram	Strain	Growth conditions
Enterococcus faecium	positive	2024474	BHI, 37˚C
Staphylococcus aureus	positive	USA300	LB, 37˚C
Klebsiella pneumoniae	negative	2029268	LB, 37˚C
Acinetobacter baumannii	negative	1952863	BHI, 37˚C
Pseudomonas aeruginosa	negative	PAO1	LB, 37˚C
Enterobacter cloacae	negative	ATCC 13047	BHI, 37˚C
Escherichia coli	negative	ATCC 25922	LB, 37˚C

Supplementary Table 3. Details of the 13 unknown ESKAPEE bacterial samples and species assignation by Random Forest analysis. *: Information blinded at the time of testing.

Unknown sample	Bacterial species*	Strain	Species assignation after Random Forest
**1**	*E. faecium*	2024474<br>(in training set)	*E. faecium*
**2**	*E. faecium*	1992081<br>**(new)**	*E. faecium*
**3**	*S. aureus*	USA300<br>(in training set)	*S. aureus*
**4**	*S. aureus*	2190153<br>**(new)**	*S. aureus*
**5**	*K. pneumoniae*	2029268<br>(in training set)	*K. pneumoniae*
**6**	*K. pneumoniae*	1963402<br>**(new)**	*K. pneumoniae*
**7**	*A. baumannii*	1952863<br>(in training set)	*A. baumannii*
**8**	*A. baumannii*	1983153<br>**(new)**	*A. baumannii*
**9**	*P. aeruginosa*	PAO1<br>(in training set)	*P. aeruginosa*
**10**	*P. aeruginosa*	2019458<br>**(new)**	*P. aeruginosa*
**11**	*E. cloacae*	ATCC 13047<br>(in training set)	*E. cloacae*
**12**	*E. coli*	ATCC 25922<br>(in training set)	*E. coli*
**13**	*E. coli*	2231734<br>**(new)**	*E. coli*

Supplementary Table 4. Total intensity (counts) obtained in the microscopy images presented in Figures 2, 4 and Supplementary Figures 8, 18.

Total intensity (counts)	SA	EF	AB	KP	EC	PA	ES
**4b**	2,621,509	8,497,476	6,640,859	0	2,471,939	4,388,395	0
**5b**	4,090,796	4,825,959	32,949,498	11,759,360	29,052,221	7,141,736	6,670,593
**7b**	15,885,295	0	0	0	0	0	0
**8b**	725,172	586,817	3,533,496	4,450,626	3,791,077	8,517,262	586,942
**8d**	316,138	1,757,920	9,279,742	0	6,169,716	195,413	1,269,771
**8e**	0	0	0	0	6,237,659	719,903	0
**NPEP1**	0	0	1.173E+09	99,070,000	3.2E+08	4.16E+08	1.5E+08
**NPEP2**	0	0	67,170,000	48,010,000	42,800,000	2.98E+08	57,510,000
**NPEP3**	0	0	48,860,000	48,520,000	22,680,000	12,251,116	1.52E+08
**PPEP1**	237,690,000	1.251E+09	0	0	0	0	0
**PPEP2**	221,150,000	13,391,627	0	0	0	0	0
**PPEP3**	65,820,000	595,254,531	0	0	0	0	0

Supplementary Table 5. Average intensity per voxel (counts) obtained in the microscopy images presented in Figures 2, 4 and Supplementary Figures 8, 18. [N/A: not available].

Average intensity per voxel (counts)	SA	EF	AB	KP	EC	PA	ES
**4b**	81	130	462	N/A	2,102	238	N/A
**5b**	116	346	631	351	232	225	257
**7b**	102	N/A	N/A	N/A	N/A	N/A	N/A
**8b**	47	48	194	177	57	269	45
**8d**	39	82	295	N/A	67	23	66
**8e**	N/A	N/A	N/A	N/A	48	33	N/A
**NPEP1**	N/A	N/A	33,606	659	8,946	2,352	2,067
**NPEP2**	N/A	N/A	1,088	946	878	1,467	1,137
**NPEP3**	N/A	N/A	778	1,239	425	118	2,384
**PPEP1**	10,878	11,133	N/A	N/A	N/A	N/A	N/A
**PPEP2**	7975	1248	N/A	N/A	N/A	N/A	N/A
**PPEP3**	6,596	13,367	N/A	N/A	N/A	N/A	N/A

Supplementary Note 1.

Optimization of the synthesis of morpholine-bearing compounds

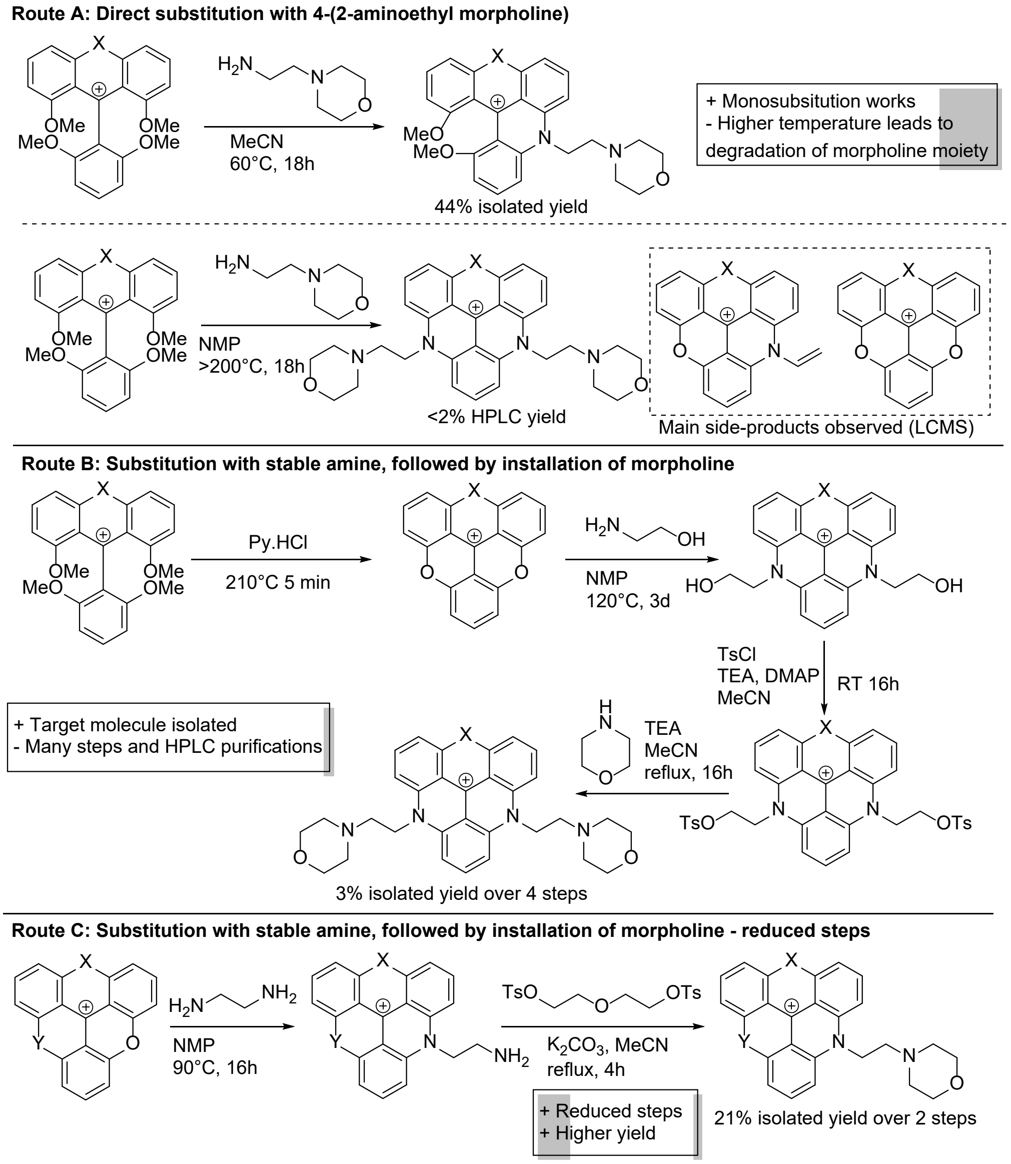

The introduction of the morpholine side chain was optimized. The first strategy (Route A) performed direct aromatic substitution using commercially available 4-(2-aminoethyl)morpholine. This route was successful for compounds **3f**, which led to the TA fluorophores **4f** and **7f**. However, the higher temperatures (>200°C) required for the synthesis of disubstituted compound **5f** led to decomposition of the morpholine moiety. The two main side-products observed were the oxo-ring-closed and allyl-substituted moieties, and the desired compound was observed in traces.

To circumvent this issue, Route B was designed. Route B employed ethanolamine, which is more stable at high temperatures, followed by ditosylation and substitution with morpholine. This route rendered the TA fluorophore **5f**, albeit in very low yields (i.e., under 5%). Therefore, we designed Route C whereby we used 1,2-diaminoethane followed by morpholine formation using commercially available diethyleneglycol ditosylate, thus affording the TA fluorophore **8f** in 2 steps and low yet acceptable yields (i.e., around 20%).

Supplementary Note 2.

Detailed lifetime data analysis

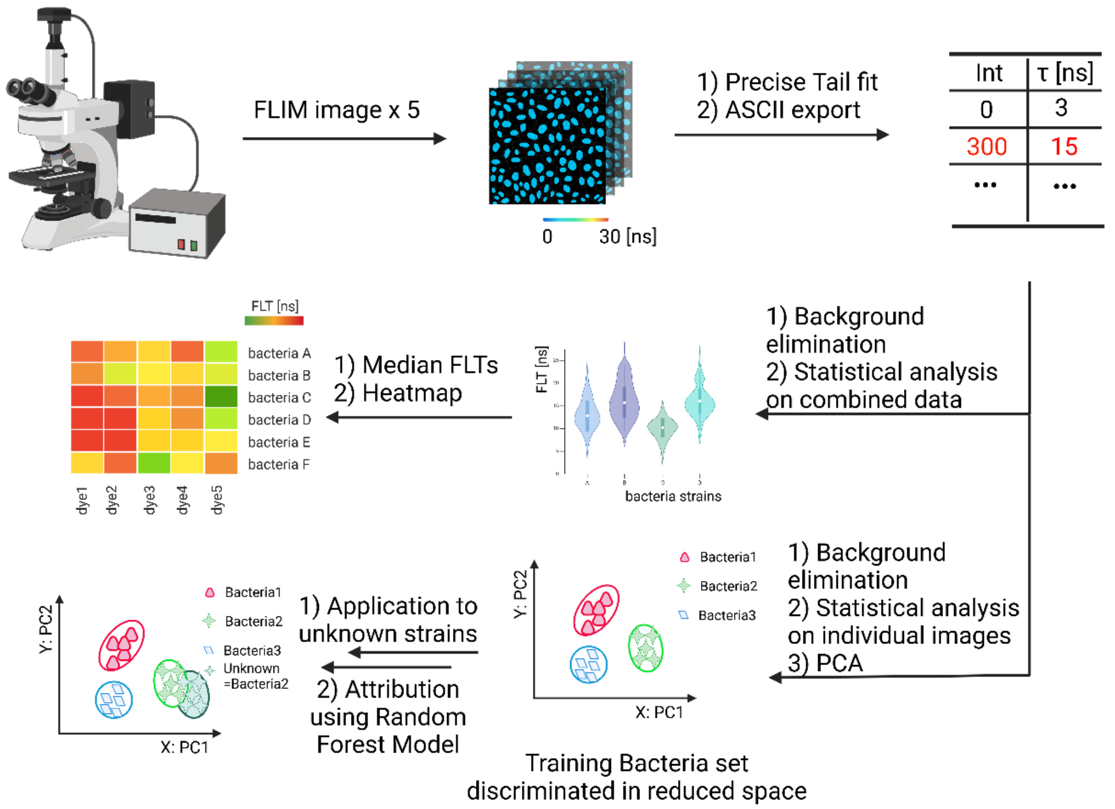

FLIM images were fitted using a tail-fit model, which was applied pixel by pixel. Data from intensity and FLIM images were exported in ASCII format as a 512×512 matrix and processed using Python and Origin Pro 2024. For each image, a Python script was used to restructure data into paired columns (intensity, fluorescence lifetime), and the background values (intensity = 0) were excluded. For distribution analysis, the fluorescence lifetime data (intensity > 0) of multiple repeats (minimum 10 field of views) of the same bacteria-TA pairs were combined. Median values as well as 95% confidence intervals were determined using Origin.

Principal Component Analysis (PCA) was performed using the median fluorescence lifetimes of individual images as described above. A covariance matrix was used, and the data normalized prior to analysis. The 95% confidence intervals were

calculated on 2D PCA plots using Origin. The assignation of bacterial species of the 13 unknown samples was performed by Random Forest and employed the 7 ESKAPEE bacterial species as the training set.

The Python script for Random Forest assignation is available as: Yanzi Zhou, Charles Lochenie, Sheelagh Duncan, Jennifer Marshall, Matthieu Vermeren, David H. Dockrell, Bethany Mills, Marc Vendrell, Lifetime chemical sensor arrays of organic fluorophores for bacterial fingerprinting, Zenodo, 10.5281/zenodo.18937893, 2026.

NMR Spectra

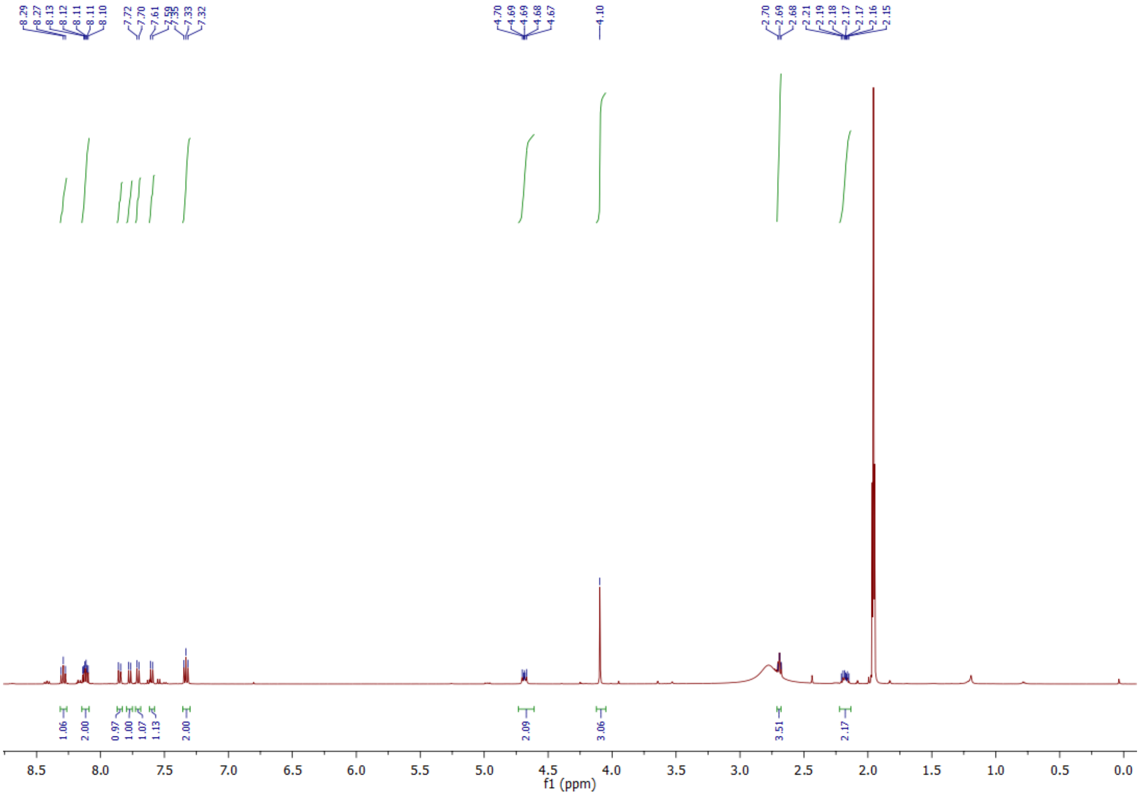

Supplementary Figure 27. ^{1}H NMR spectrum of **4a** in CD_3CN (500 MHz).

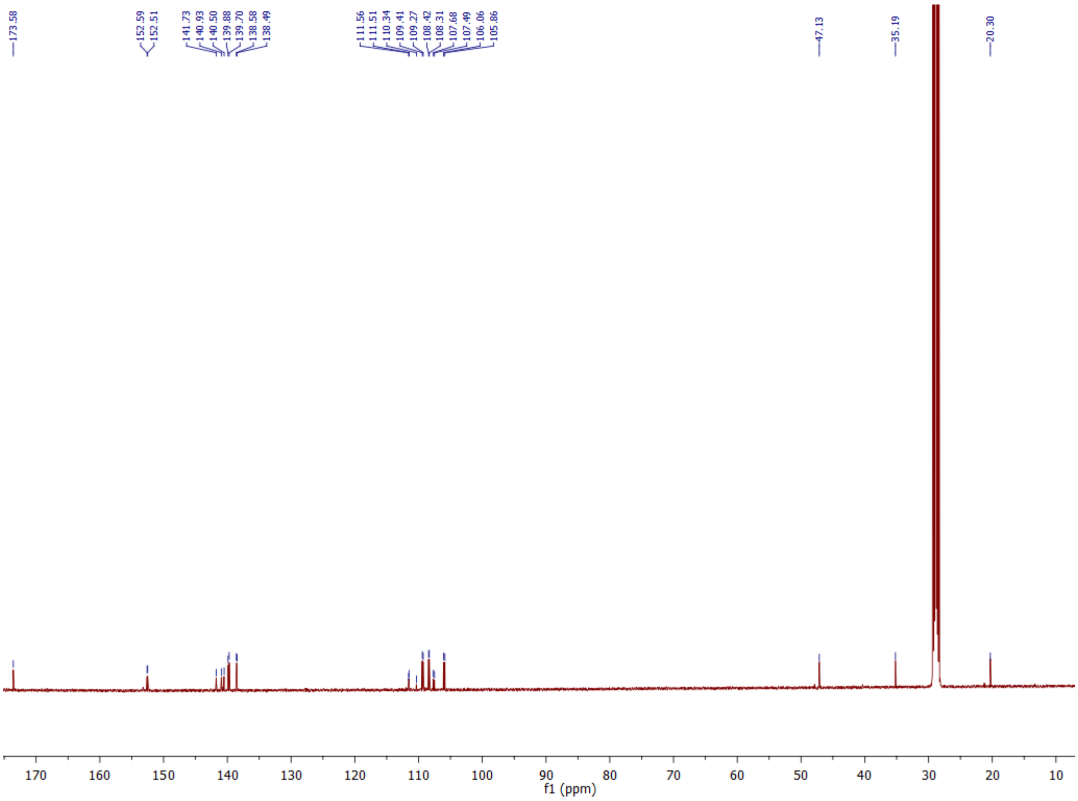

Supplementary Figure 28. ^{13}C NMR spectrum of **4a** in CD_3CN (126 MHz).

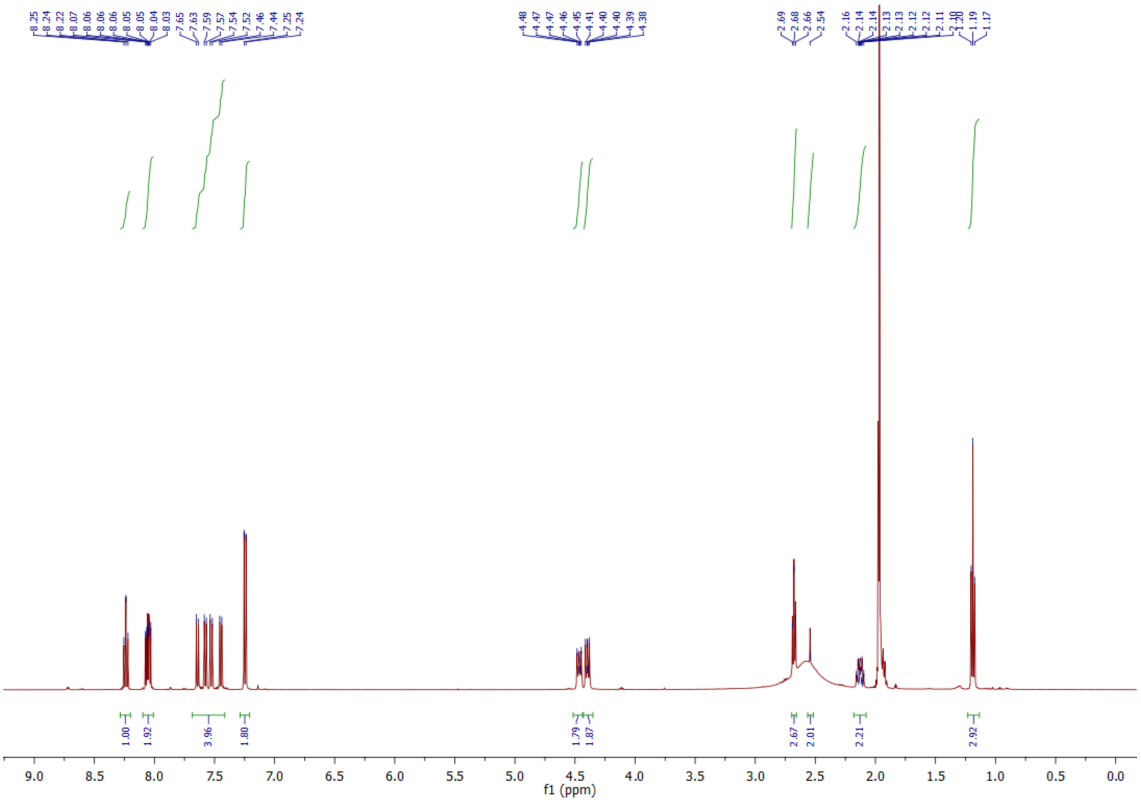

Supplementary Figure 29. ^{1}H NMR spectrum of **4b** in CD_3CN (500 MHz).

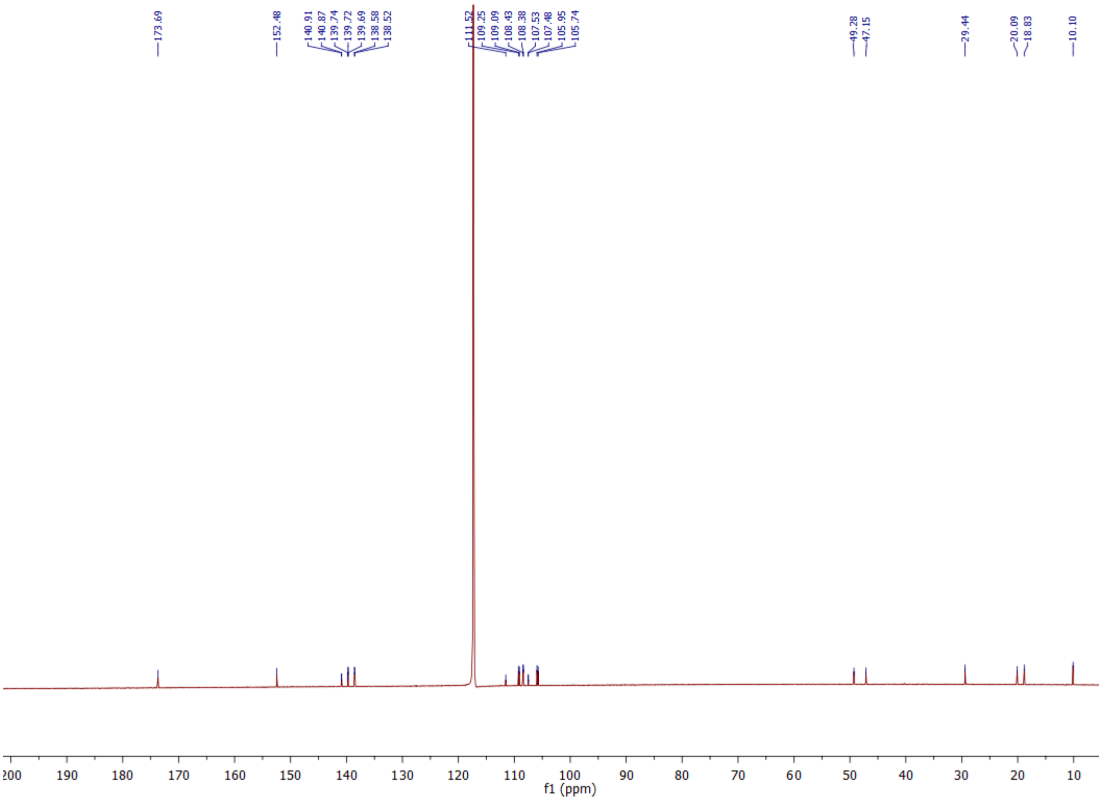

Supplementary Figure 30.^{13}C NMR spectrum of **4b** in CD_3CN (126 MHz).

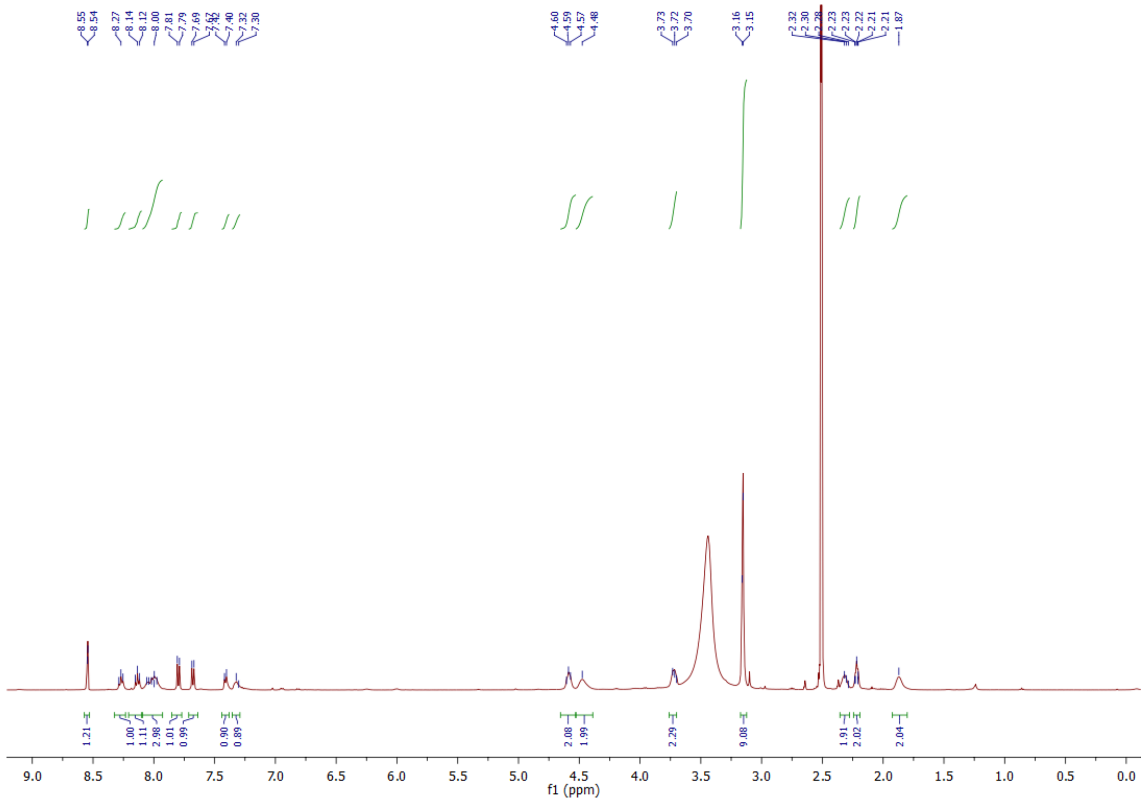

Supplementary Figure 31. ^{1}H NMR spectrum of **4d** in DMSO-d_6 (500 MHz).

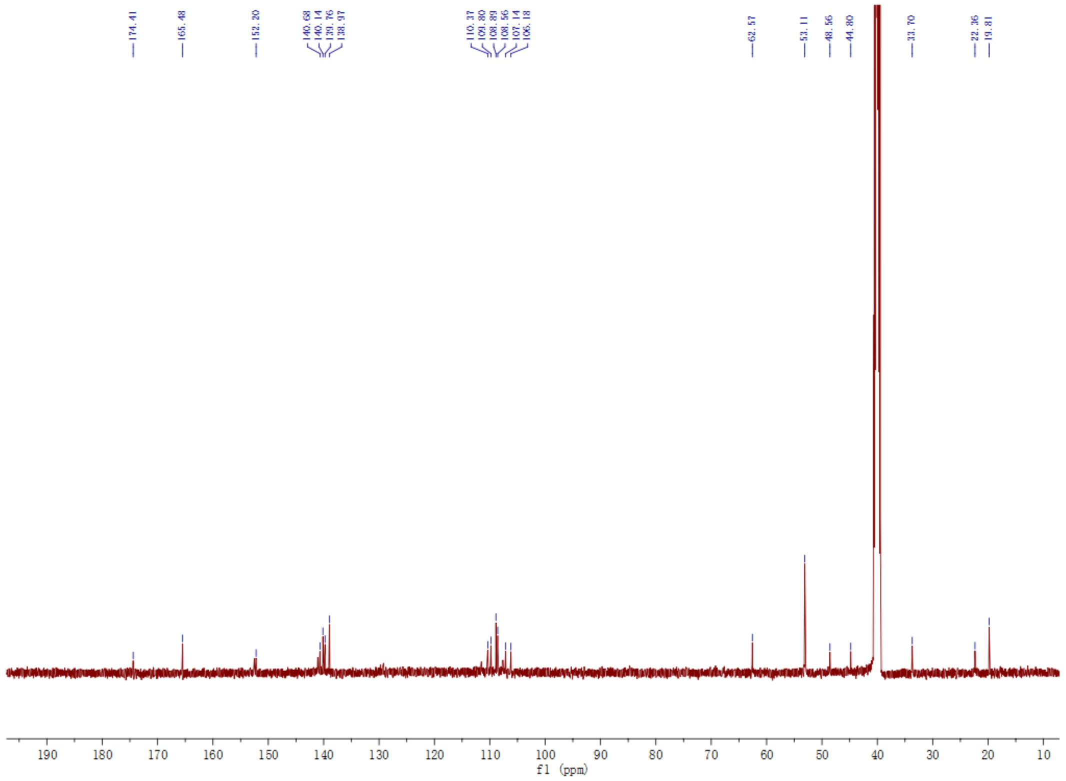

Supplementary Figure 32. ^{13}C NMR spectrum of **4d** in DMSO-d_6 (126 MHz).

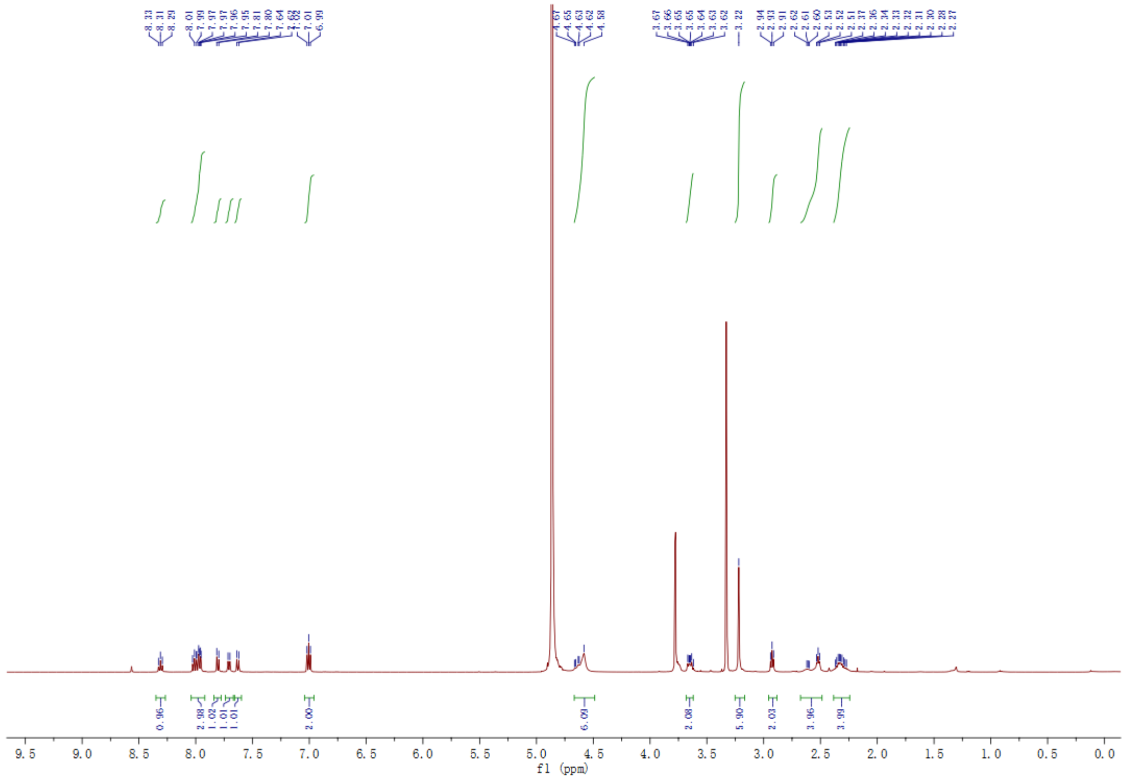

Supplementary Figure 33.^{1}H NMR spectrum of **4e** in CD_3OD (500 MHz).

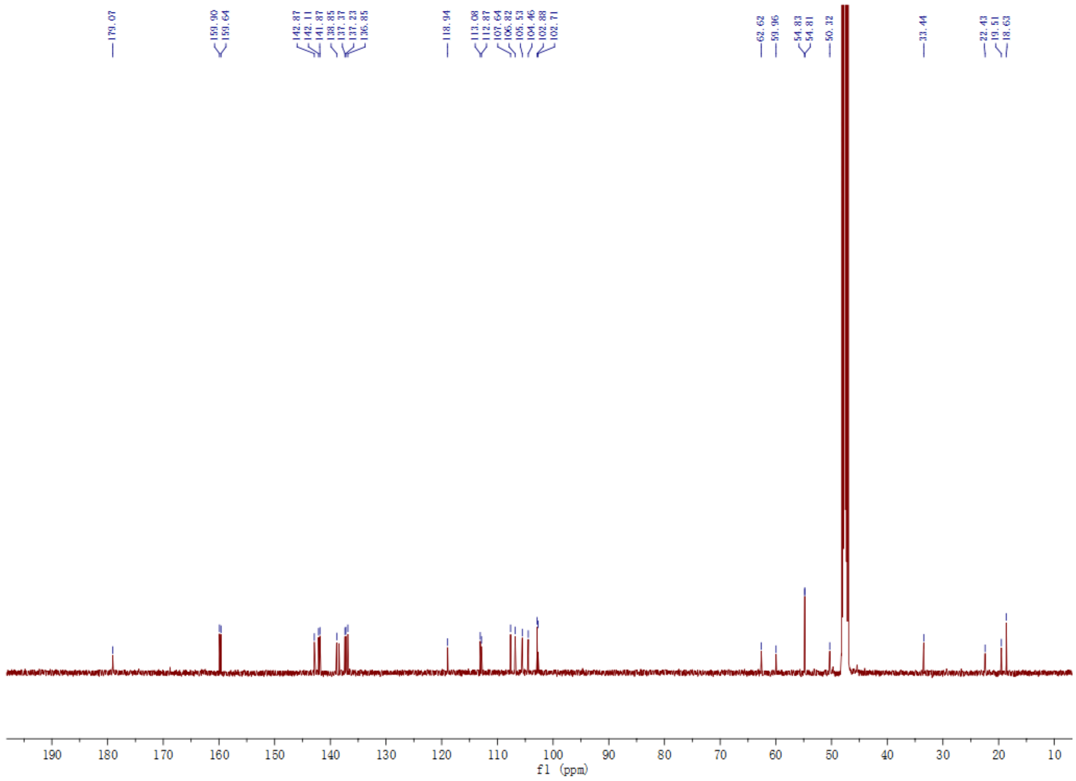

Supplementary Figure 34.^{13}C NMR spectrum of **4e** in CD_3OD (126 MHz).

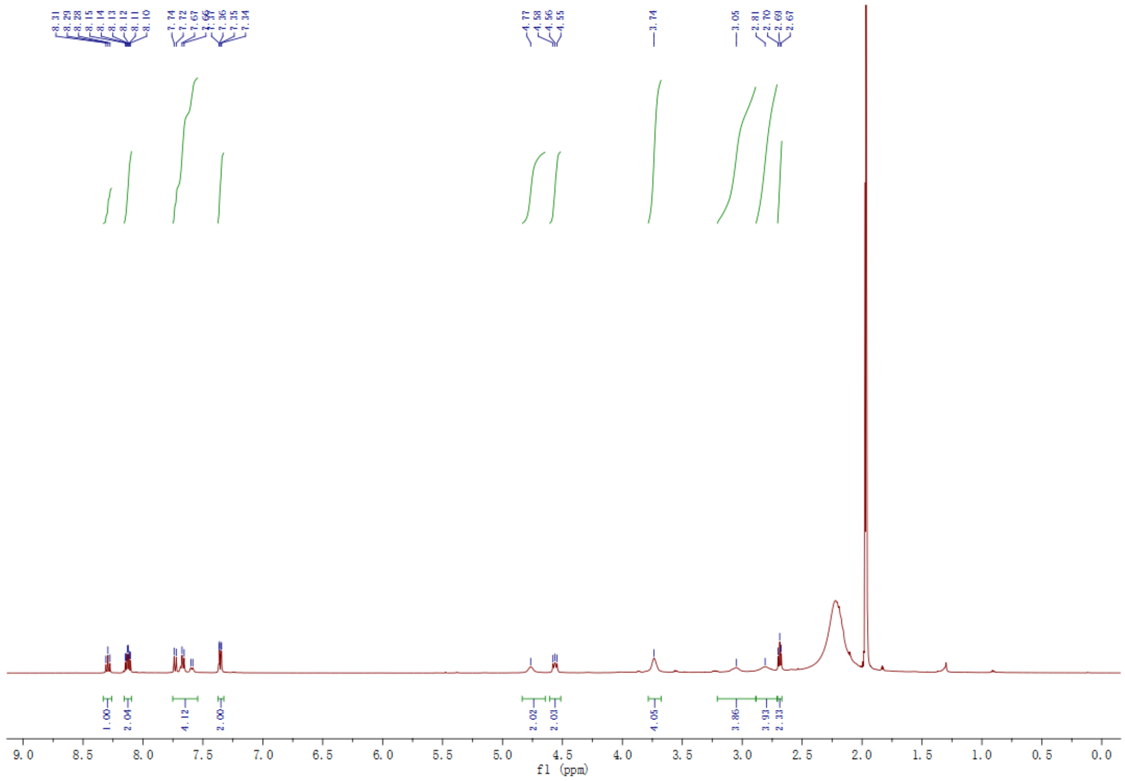

Supplementary Figure 35. ^{1}H NMR spectrum of **4f** in CD_3CN (500 MHz).

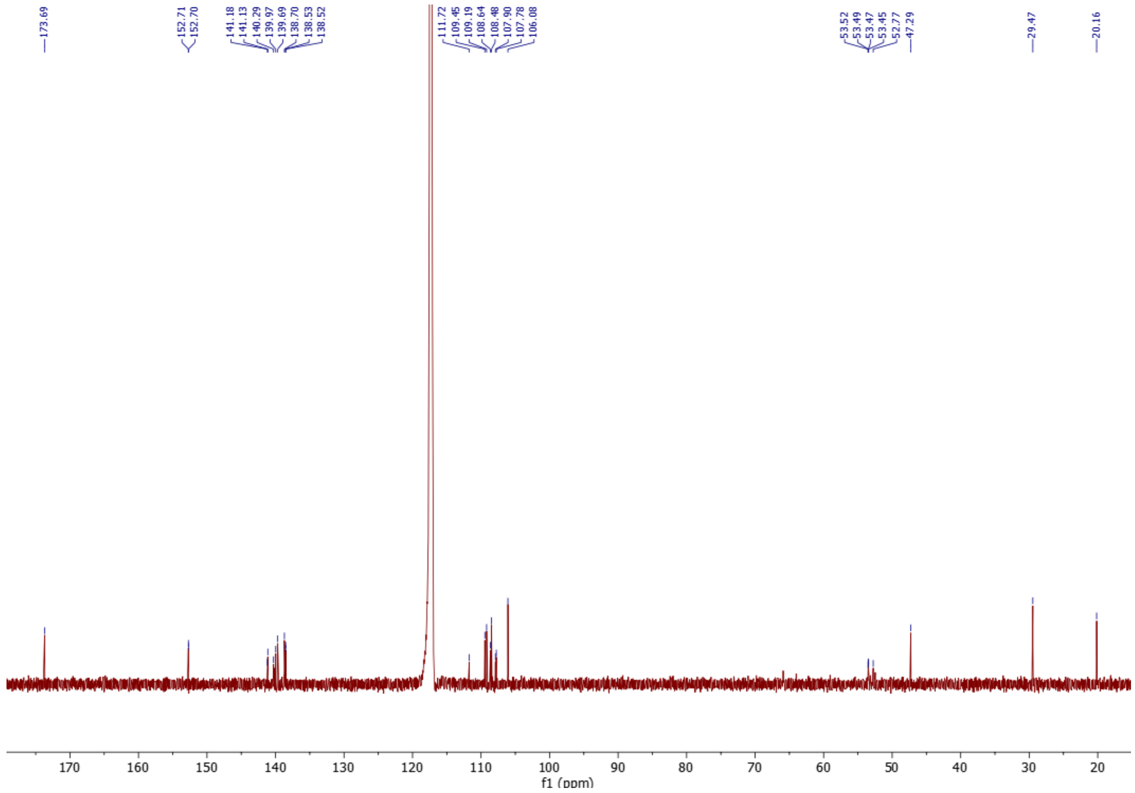

Supplementary Figure 36. ^{13}C NMR spectrum of **4f** in CD_3CN (126 MHz).

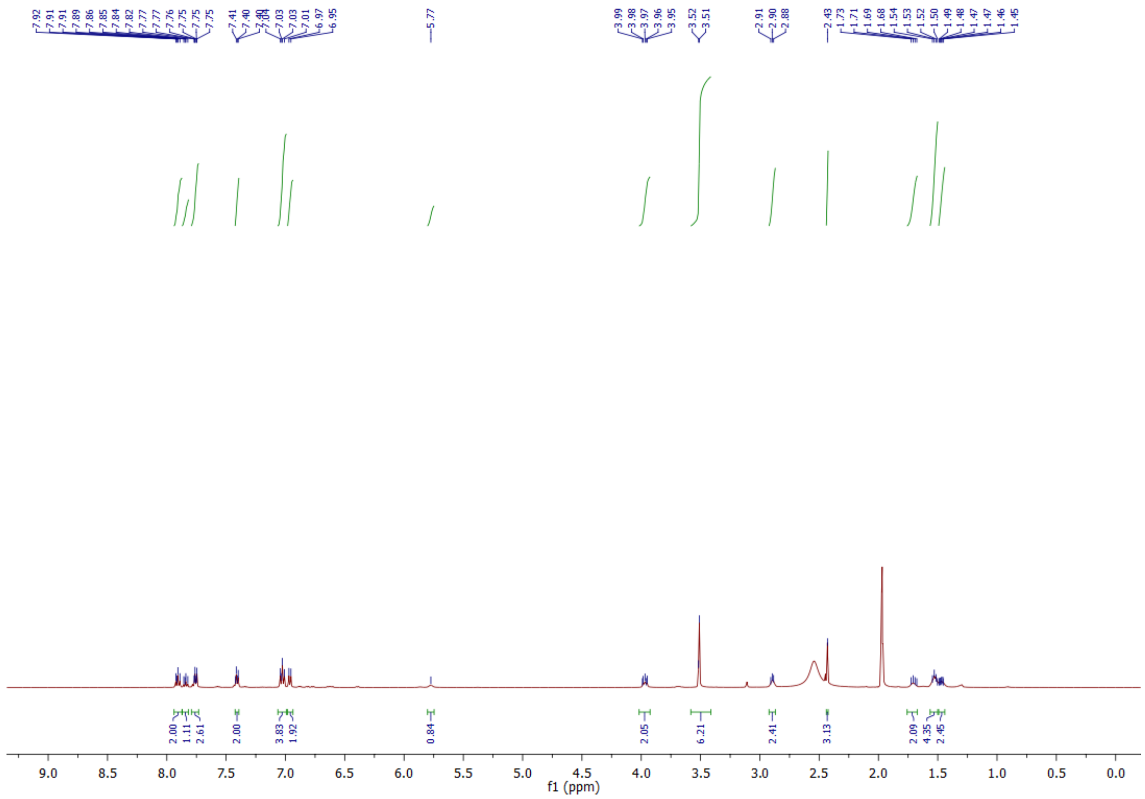

Supplementary Figure 37. ^{1}H NMR spectrum of **5a** in CD_3CN (500 MHz).

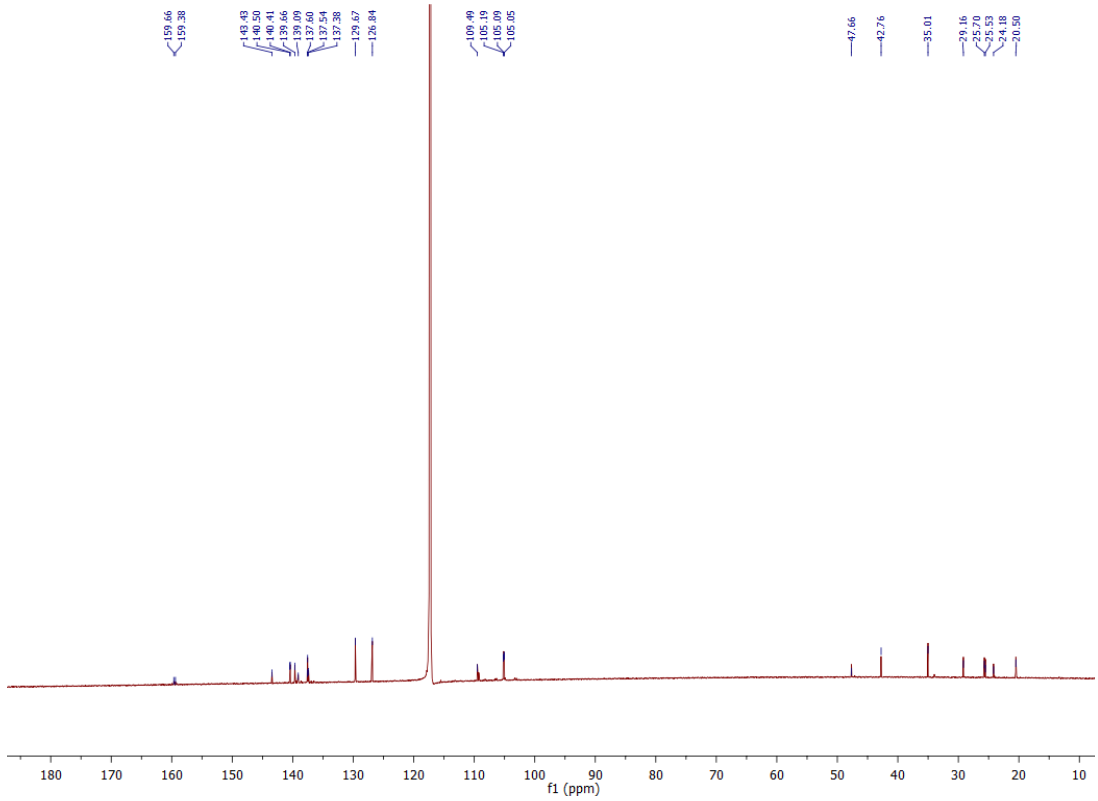

Supplementary Figure 38. ^{13}C NMR spectrum of **5a** in CD_3CN (126 MHz).

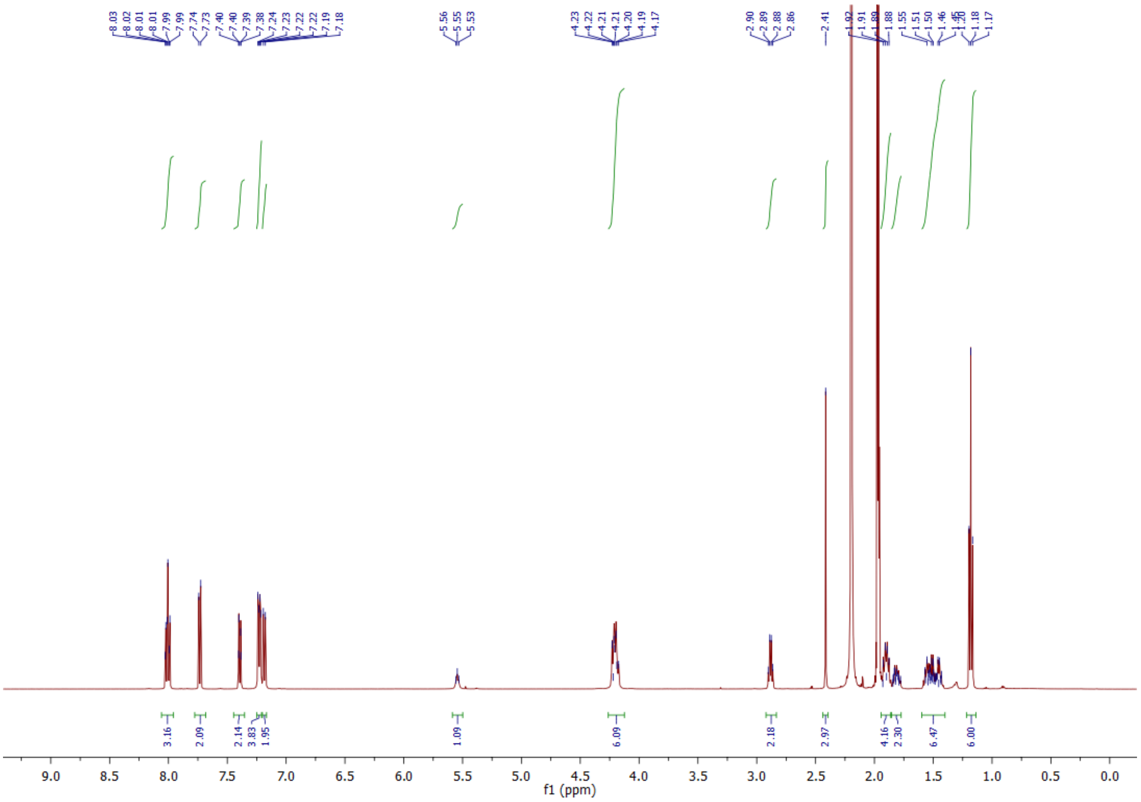

Supplementary Figure 39. ^{1}H NMR spectrum of **5b** in CD_3CN (500 MHz).

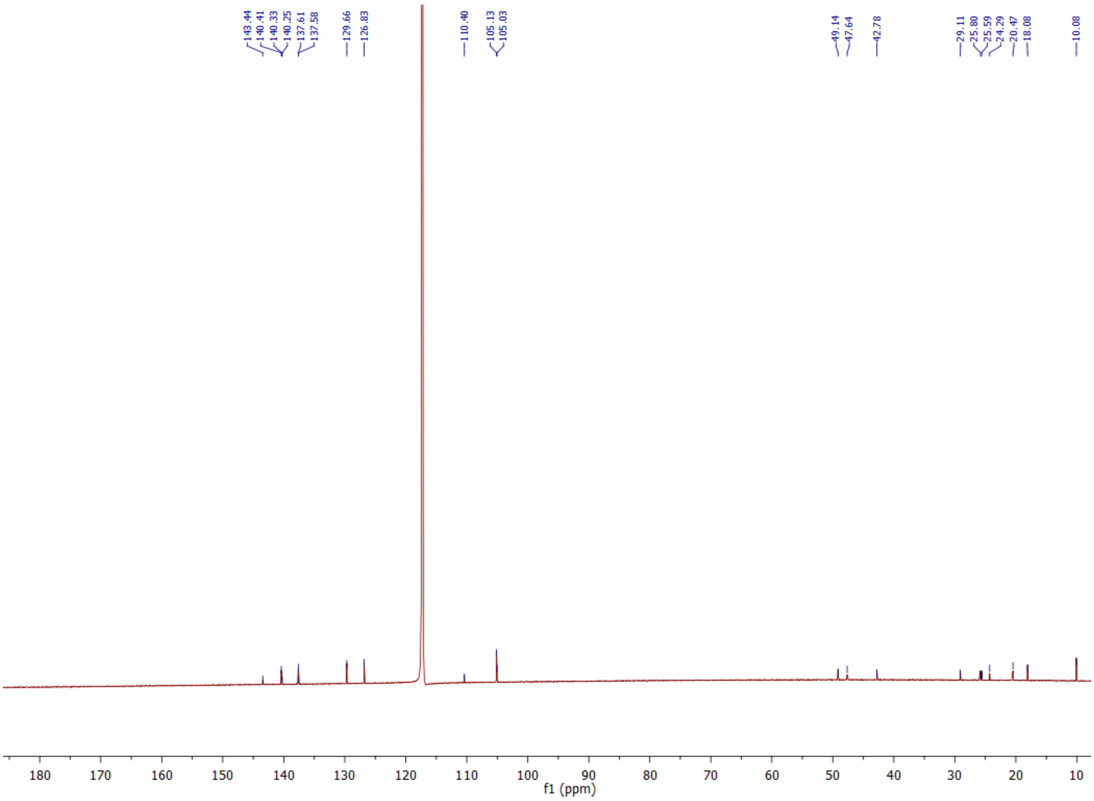

Supplementary Figure 40. ^{13}C NMR spectrum of **5b** in CD_3CN (126 MHz).

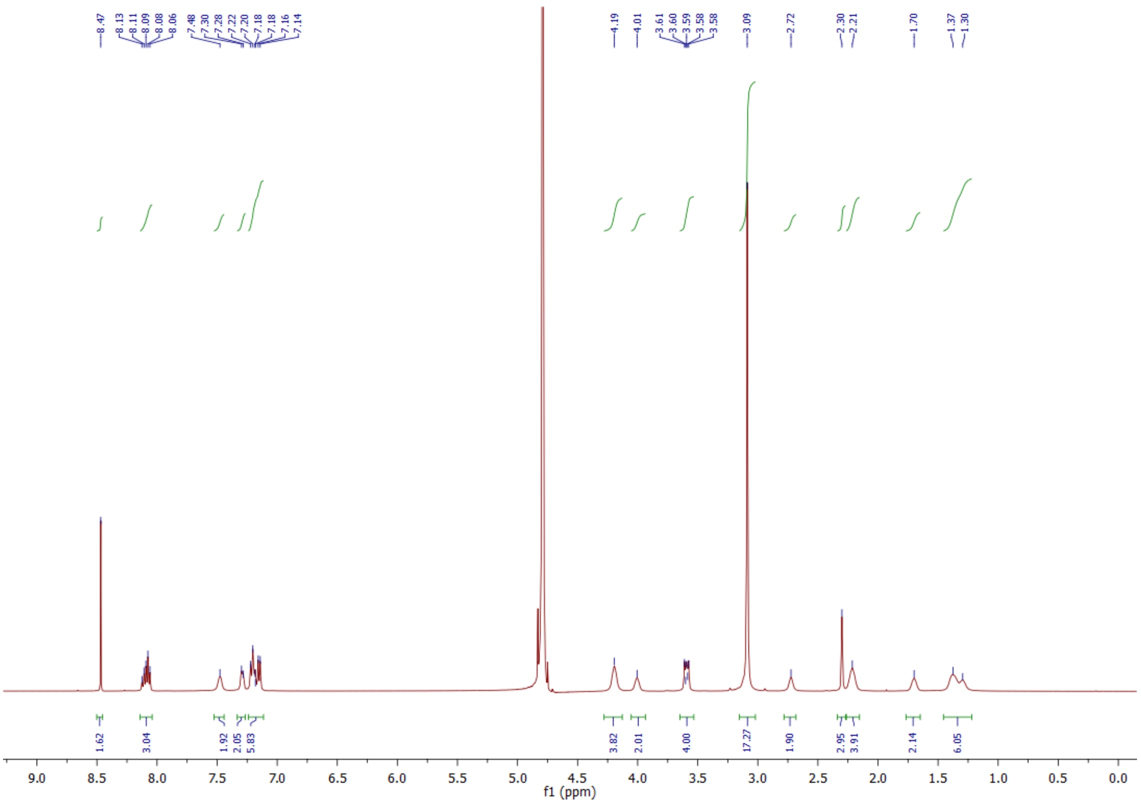

Supplementary Figure 41. ^{1}H NMR spectrum of **5d** in D_2O (500 MHz).

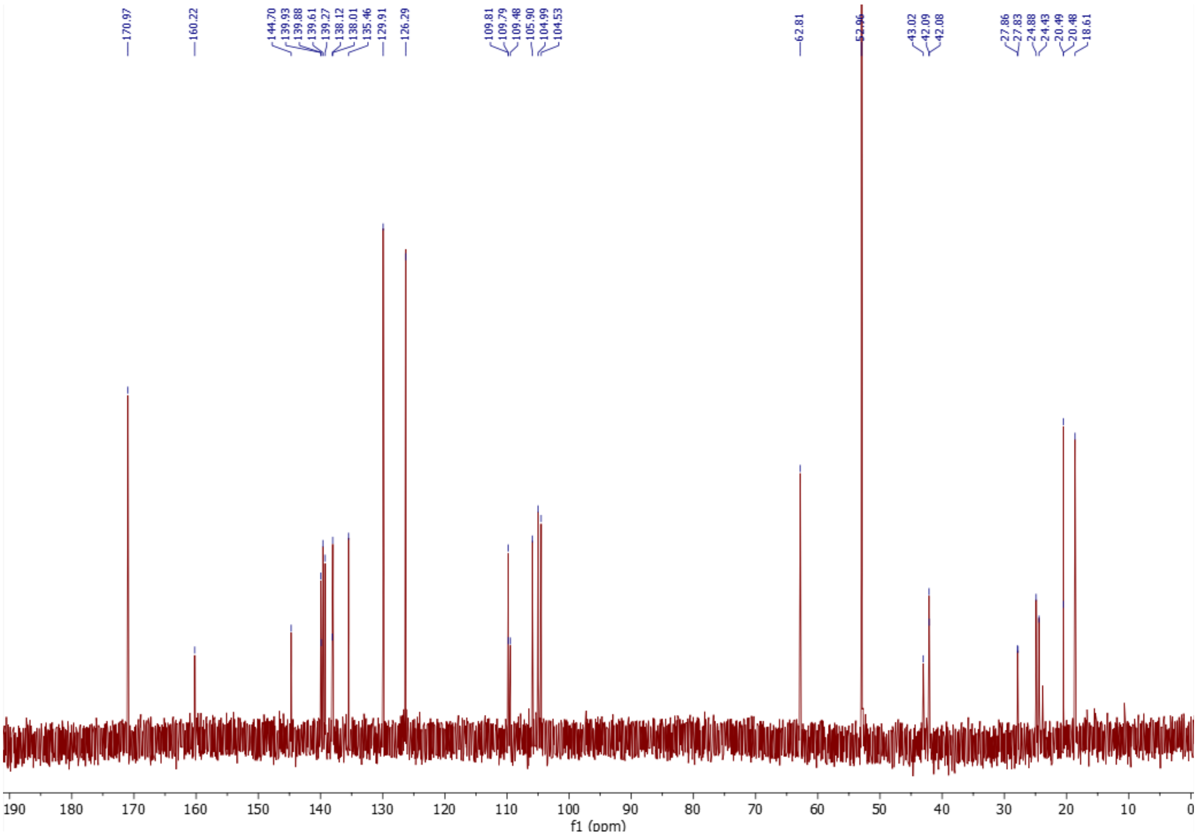

Supplementary Figure 42. ^{13}C NMR spectrum of **5d** in D_2O (126 MHz).

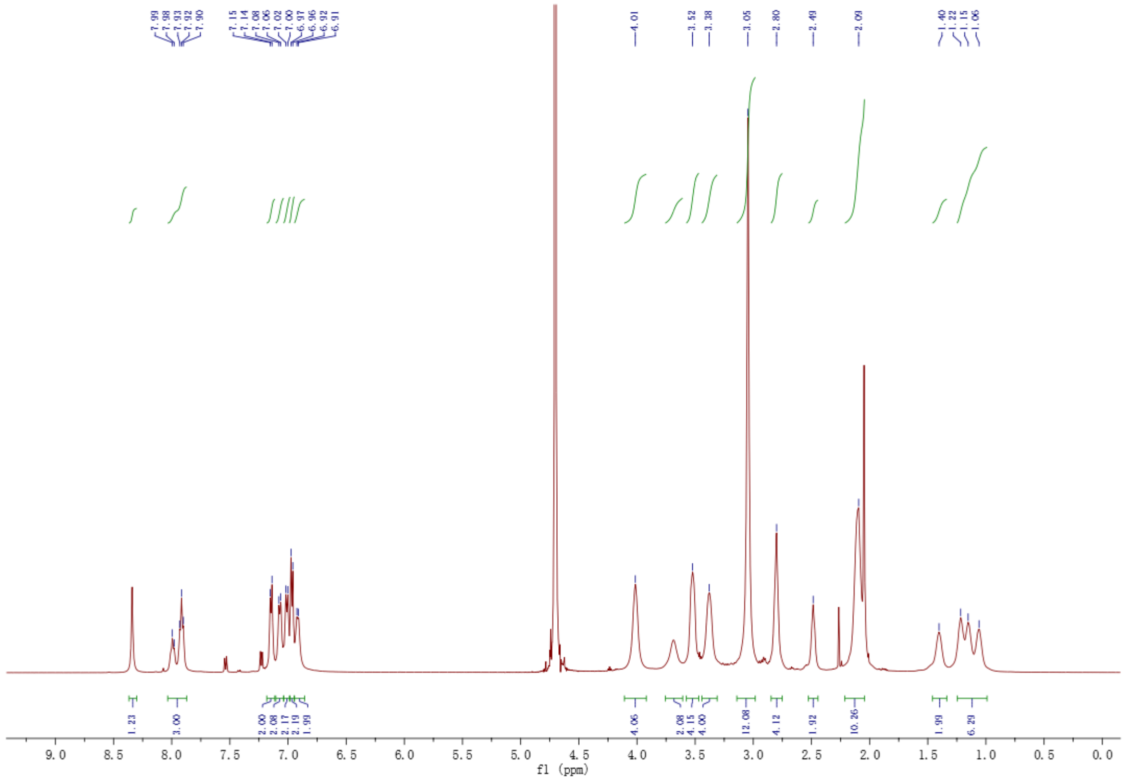

Supplementary Figure 43. ^{1}H NMR spectrum of **5e** in D_2O (500 MHz).

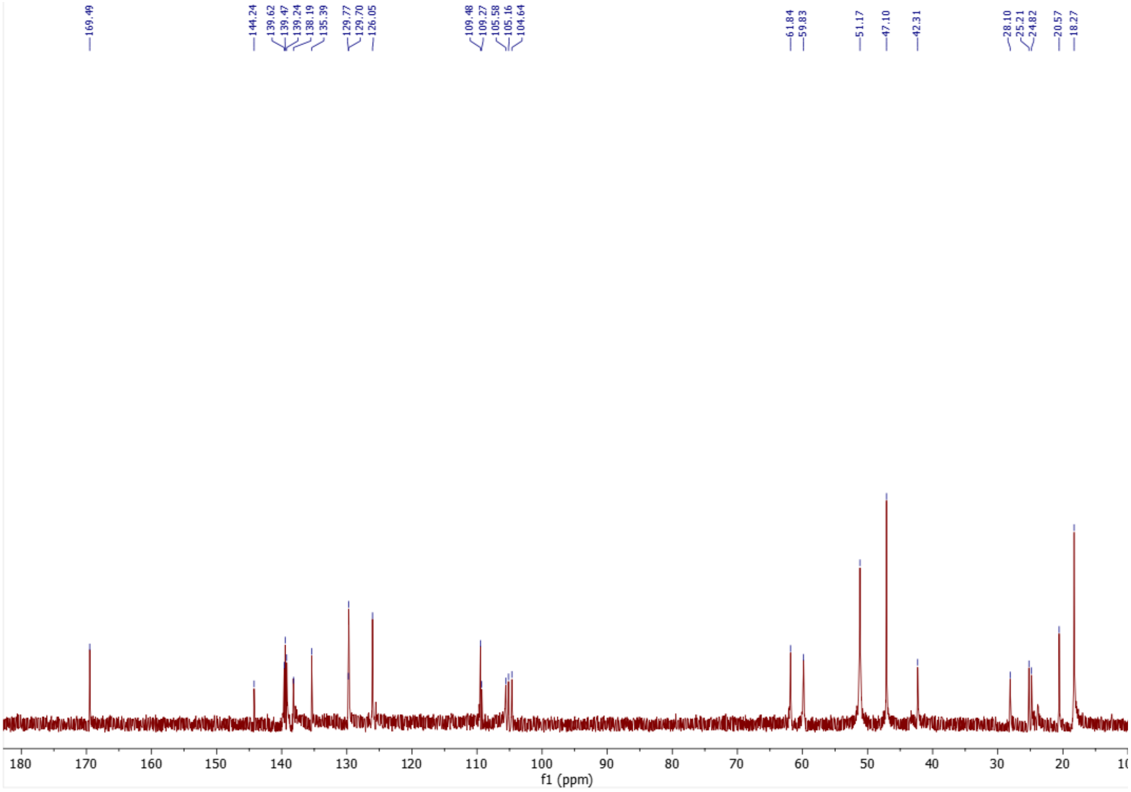

Supplementary Figure 44. ^{13}C NMR spectrum of **5e** in in D_2O (126 MHz).

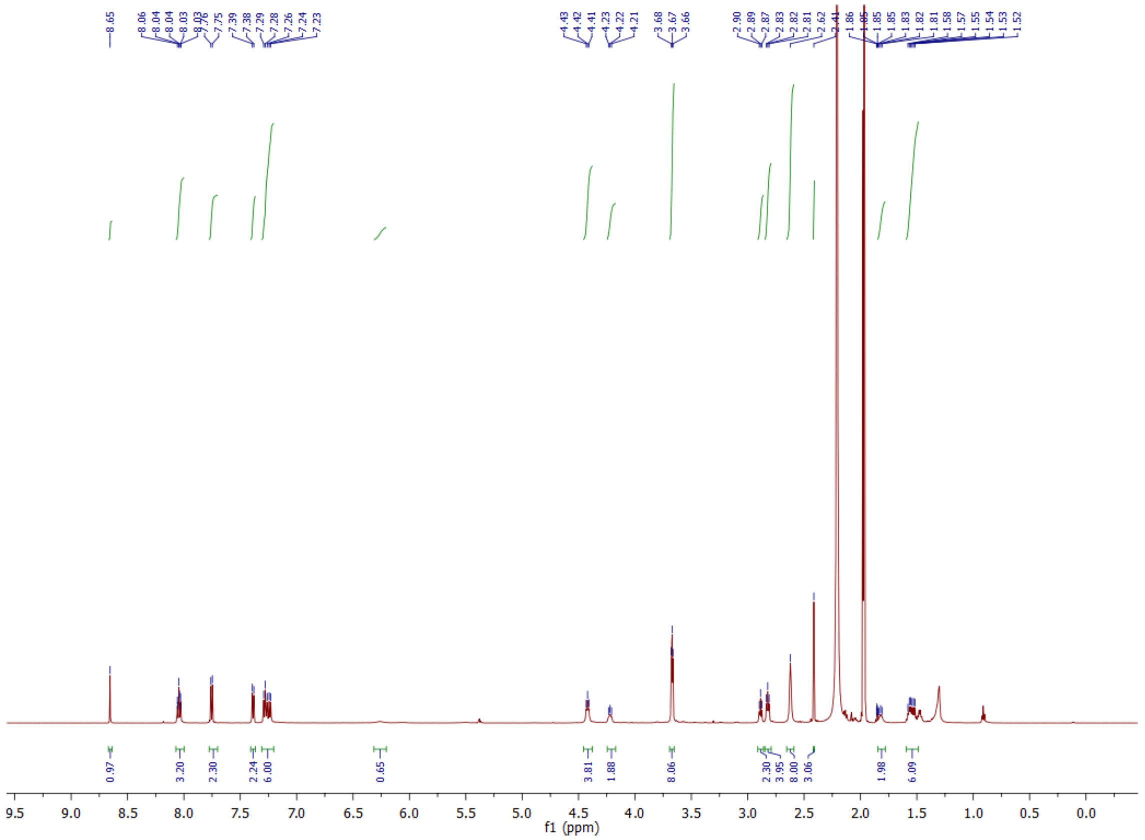

Supplementary Figure 45. ^{1}H NMR spectrum of **5f** in CD_3CN (500 MHz).

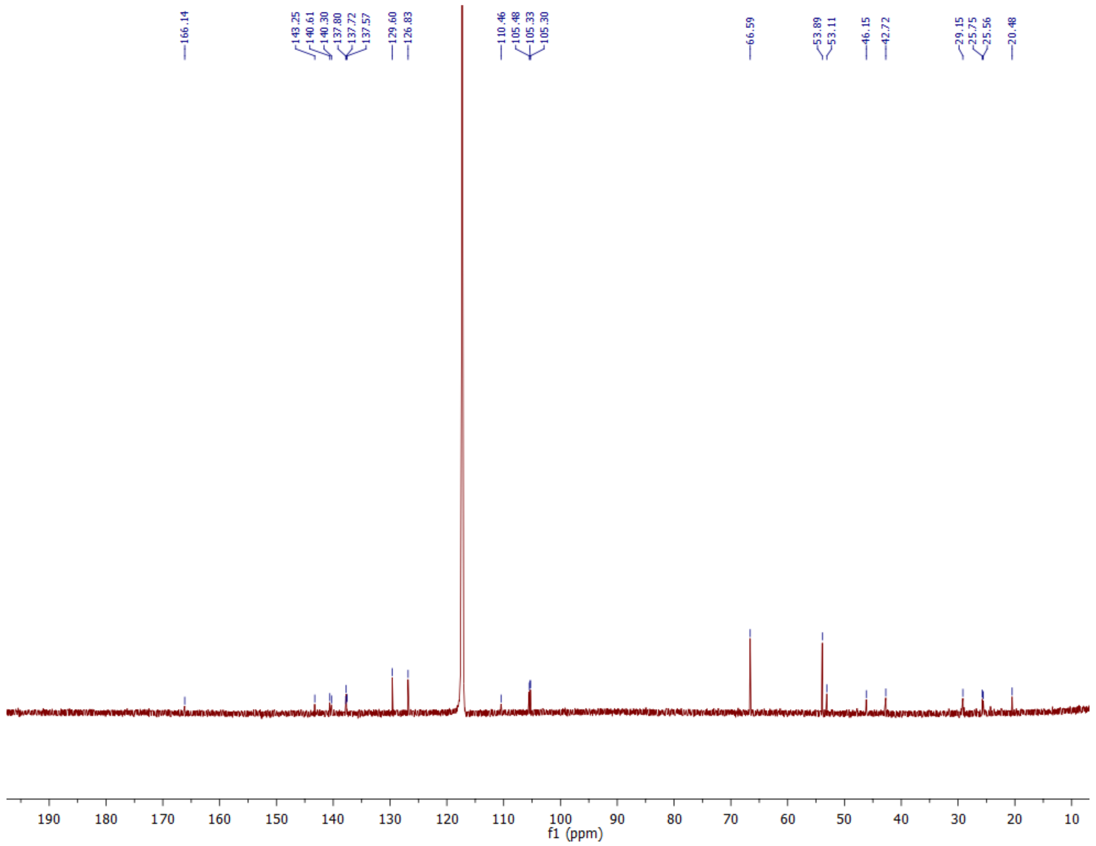

Supplementary Figure 46. ^{13}C NMR spectrum of **5f** in CD_3CN (126 MHz).

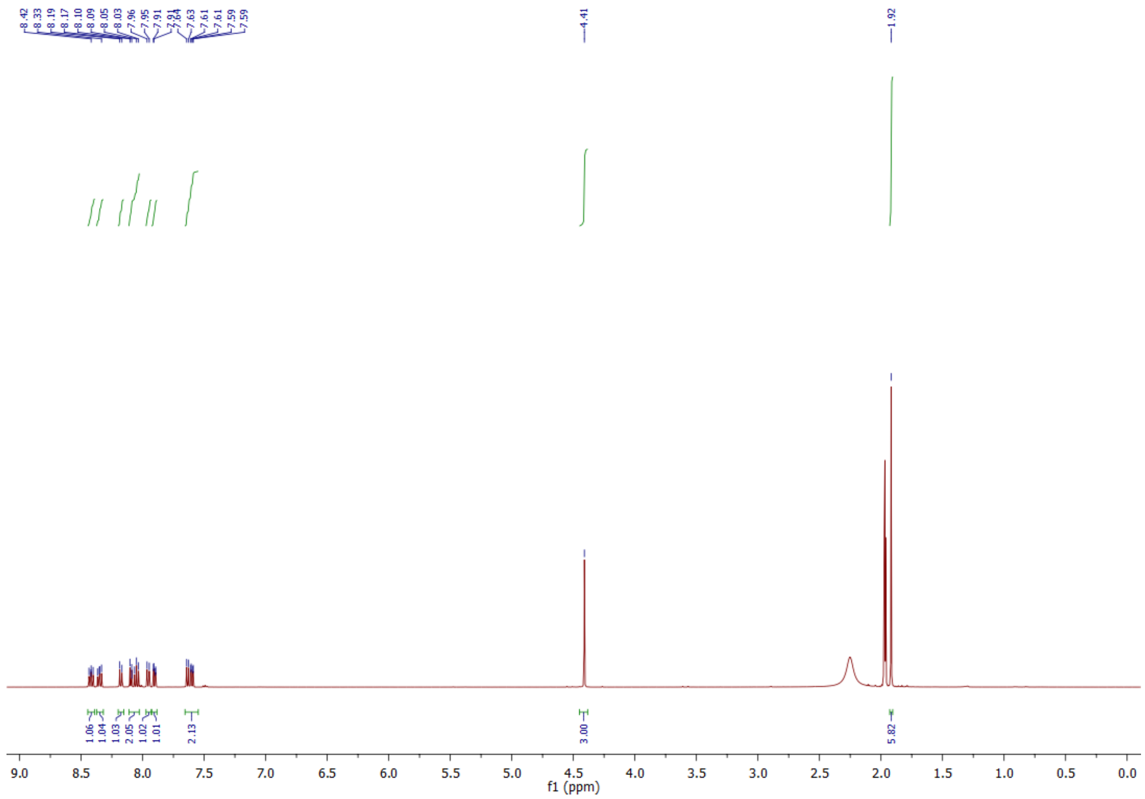

Supplementary Figure 47. ^{1}H NMR spectrum of **7a** in CD_3CN (500 MHz).

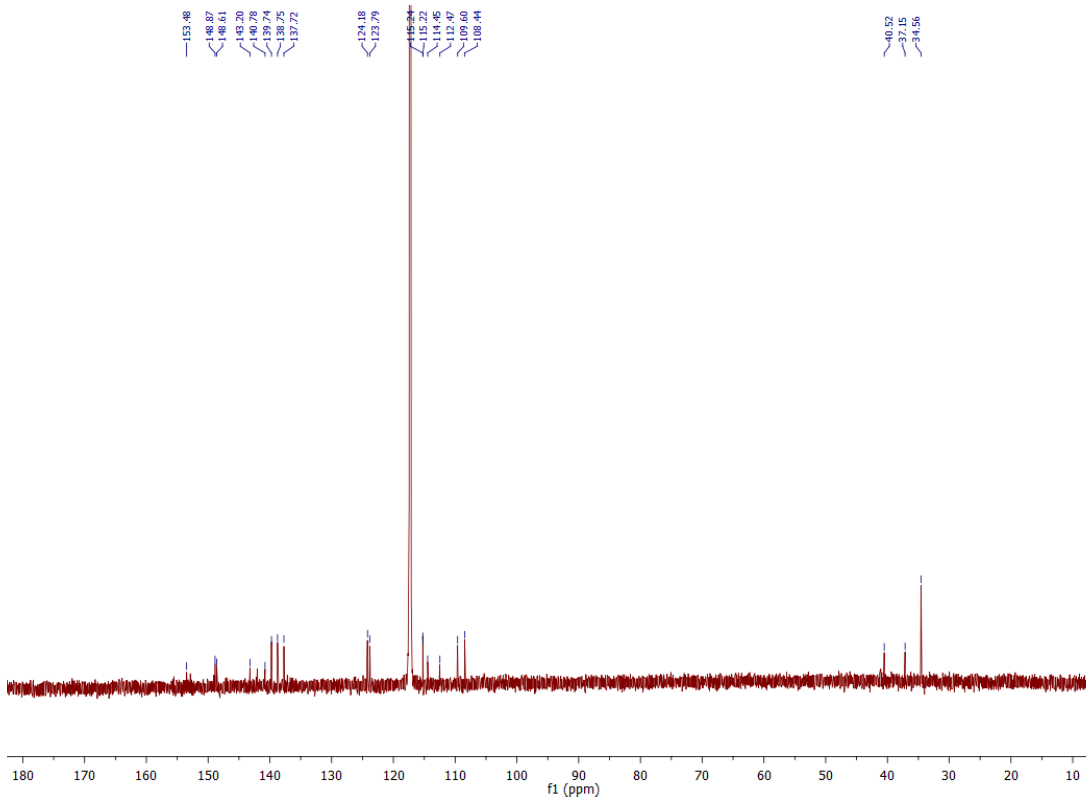

Supplementary Figure 48. ^{13}C NMR spectrum of **7a** in CD_3CN (126 MHz).

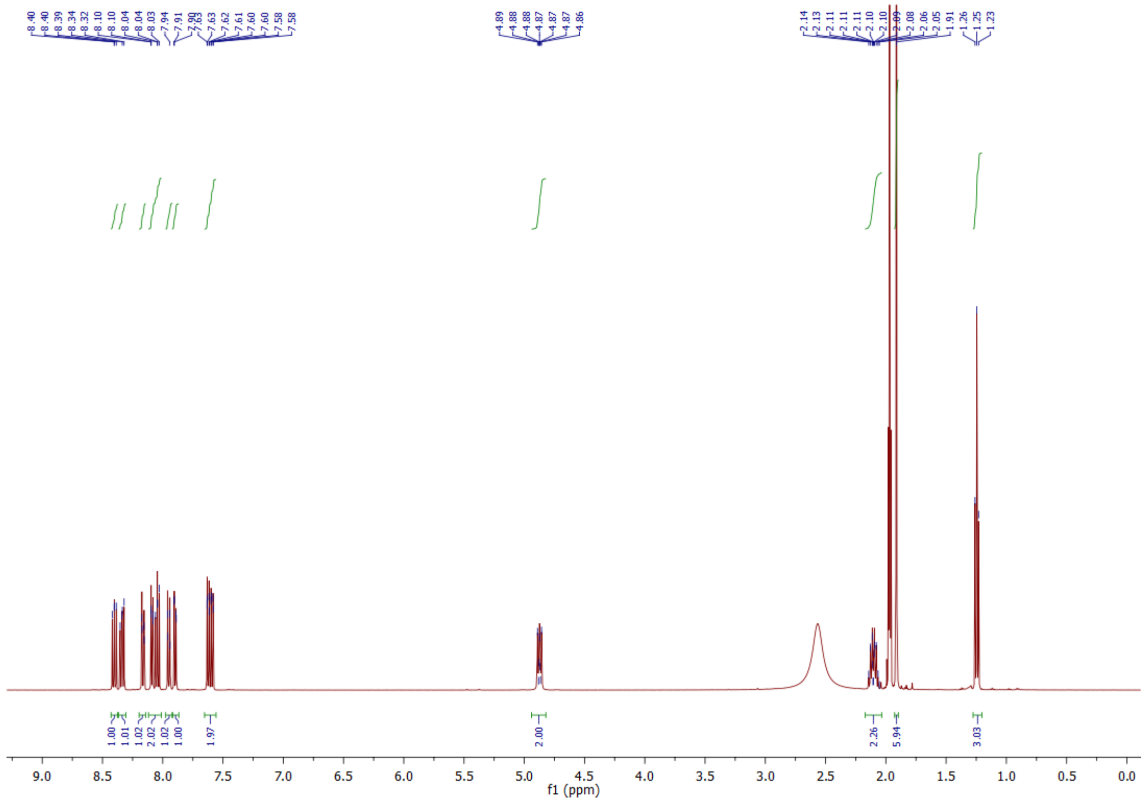

Supplementary Figure 49. ^{1}H NMR spectrum of **7b** in CD_3CN (500 MHz).

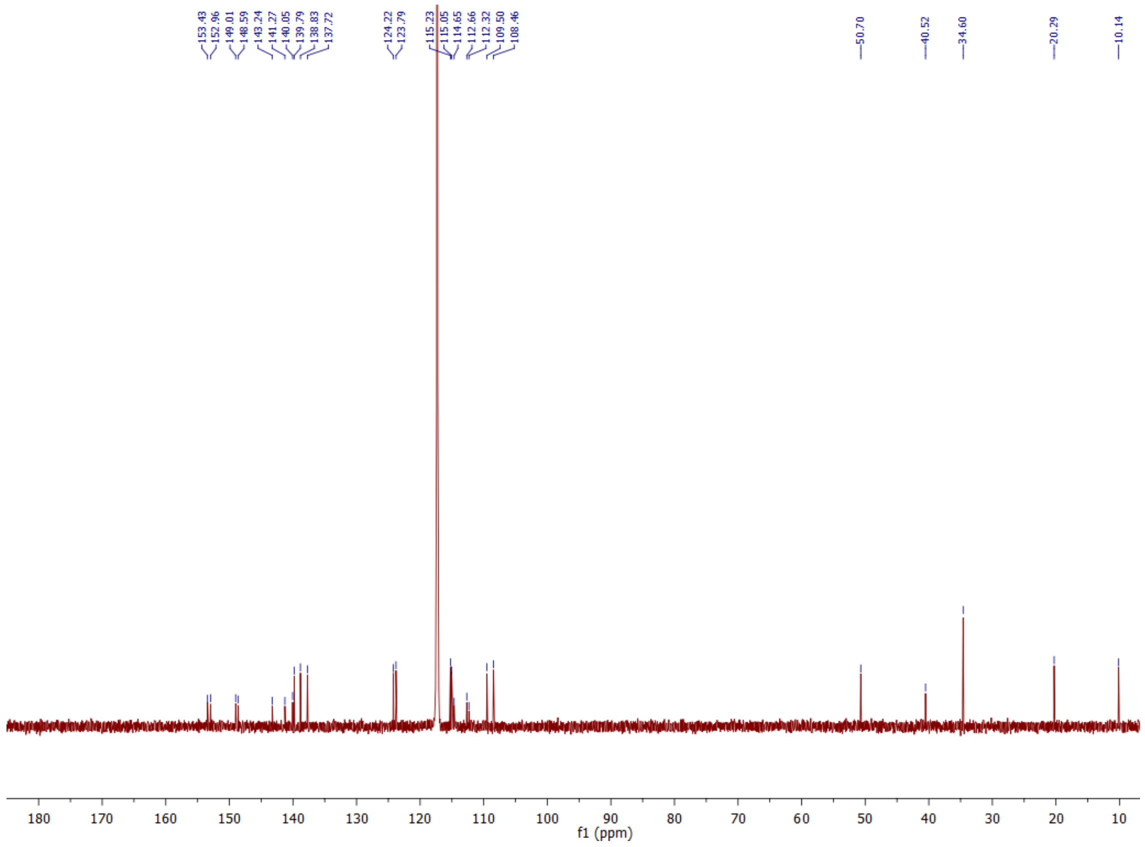

Supplementary Figure 50. ^{13}C NMR spectrum of **7b** in CD_3CN (126 MHz).

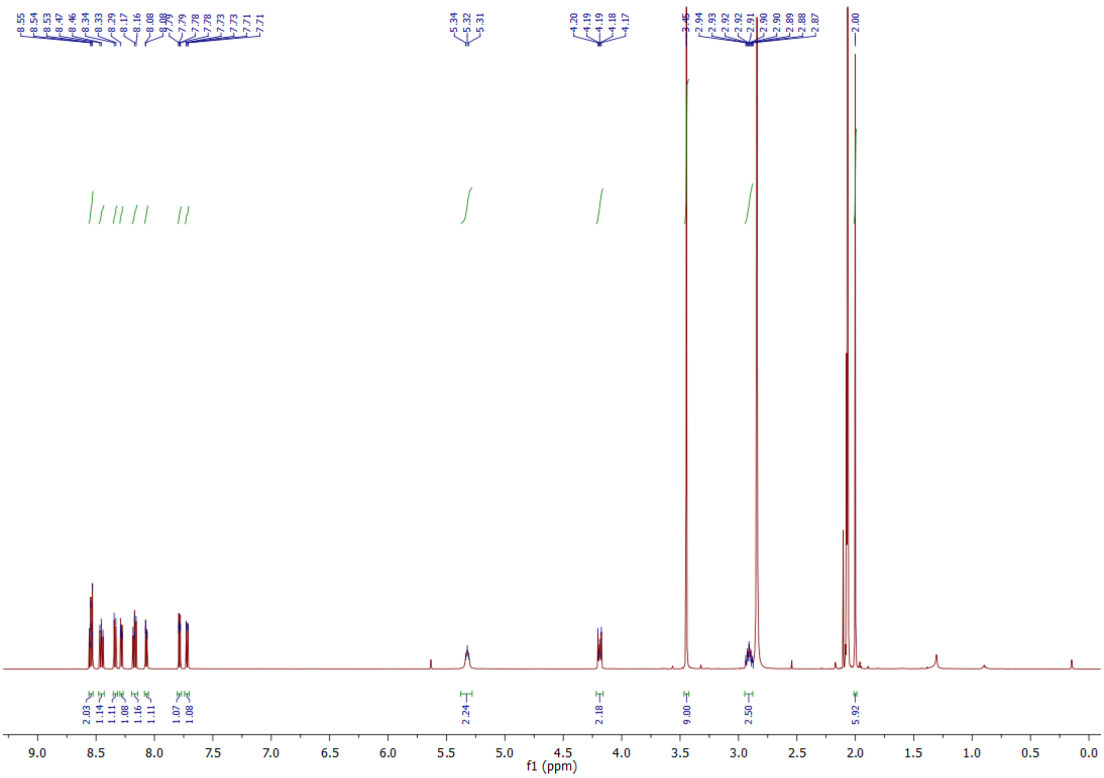

Supplementary Figure 51. ^{1}H NMR spectrum of **7d** in $(CD_3)_2CO$ (600 MHz).

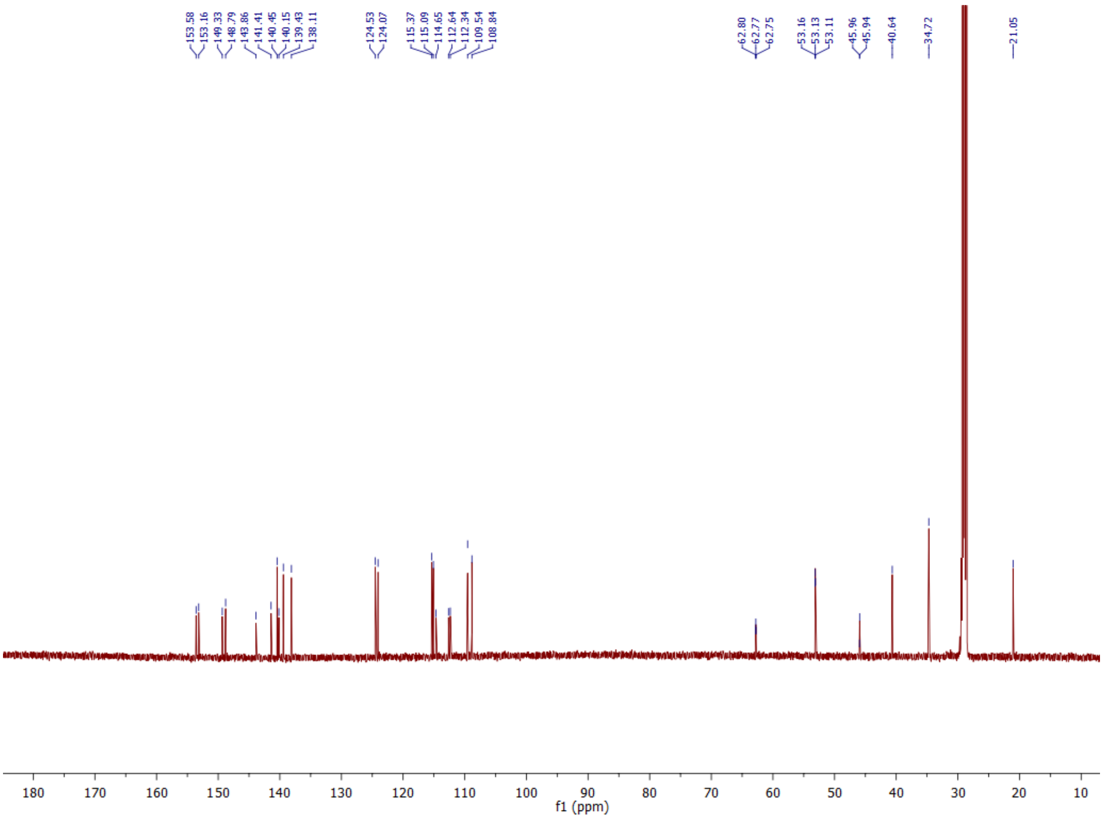

Supplementary Figure 52. ^{13}C NMR spectrum of **7d** in $(CD_3)_2CO$ (151 MHz).

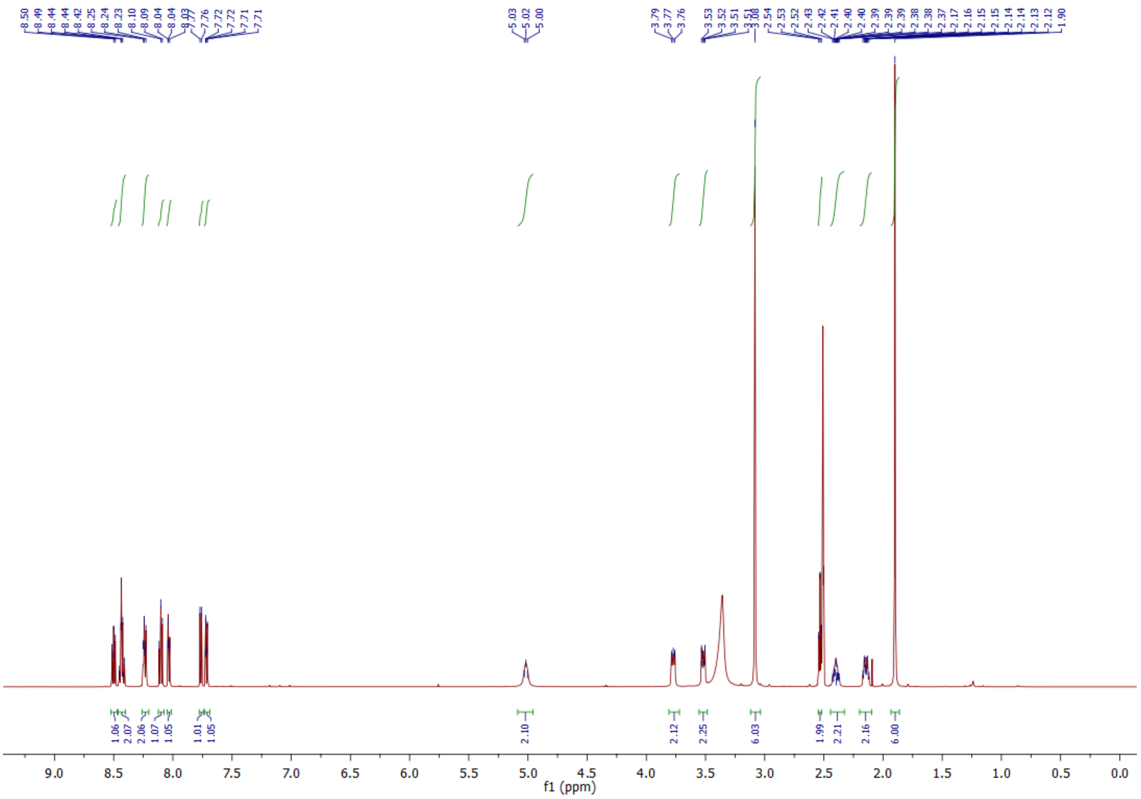

Supplementary Figure 53. ^{1}H NMR spectrum of **7e** in DMSO-d_6 (600 MHz).

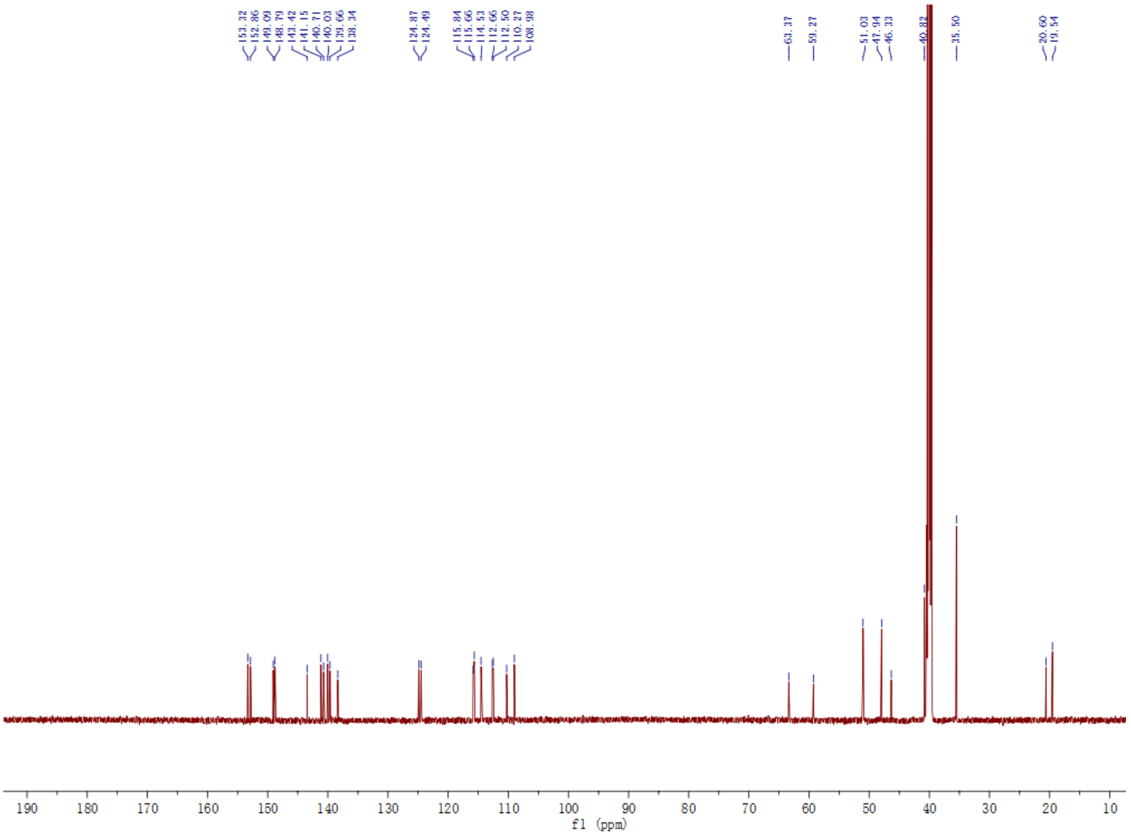

Supplementary Figure 54. ^{13}C NMR spectrum of **7e** in DMSO-d_6 (151 MHz).

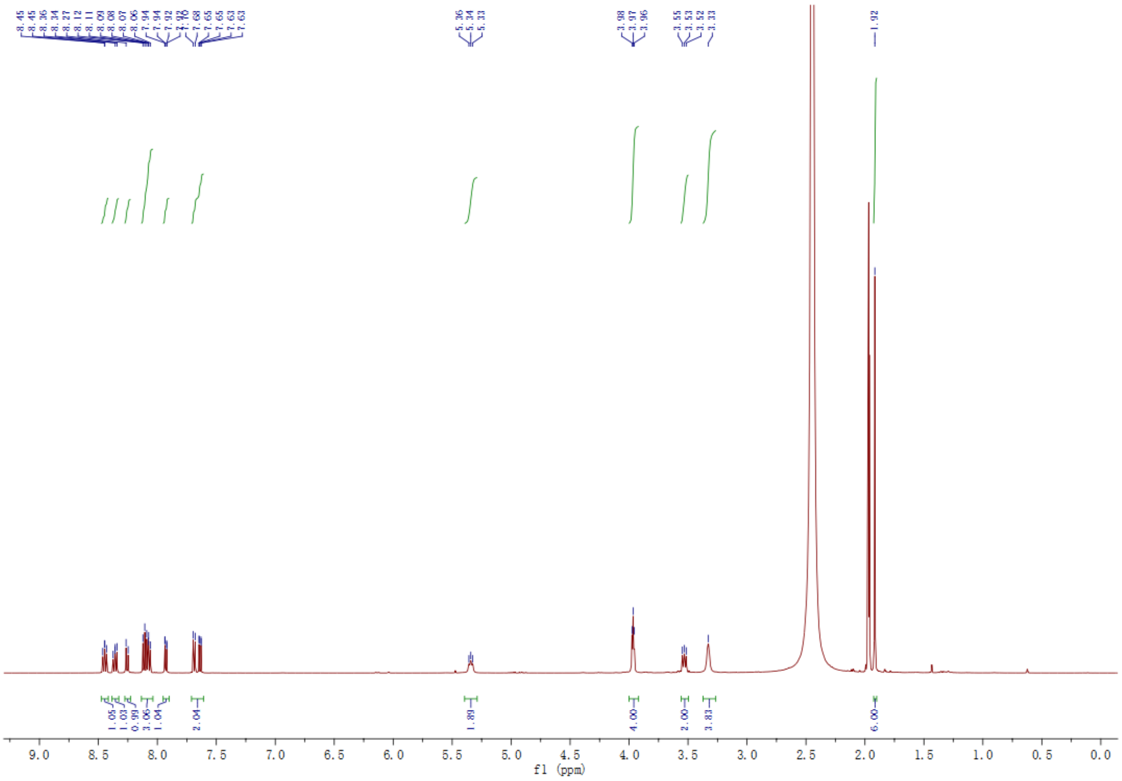

Supplementary Figure 55. ^{1}H NMR spectrum of **7f** in CD_3CN (500 MHz).

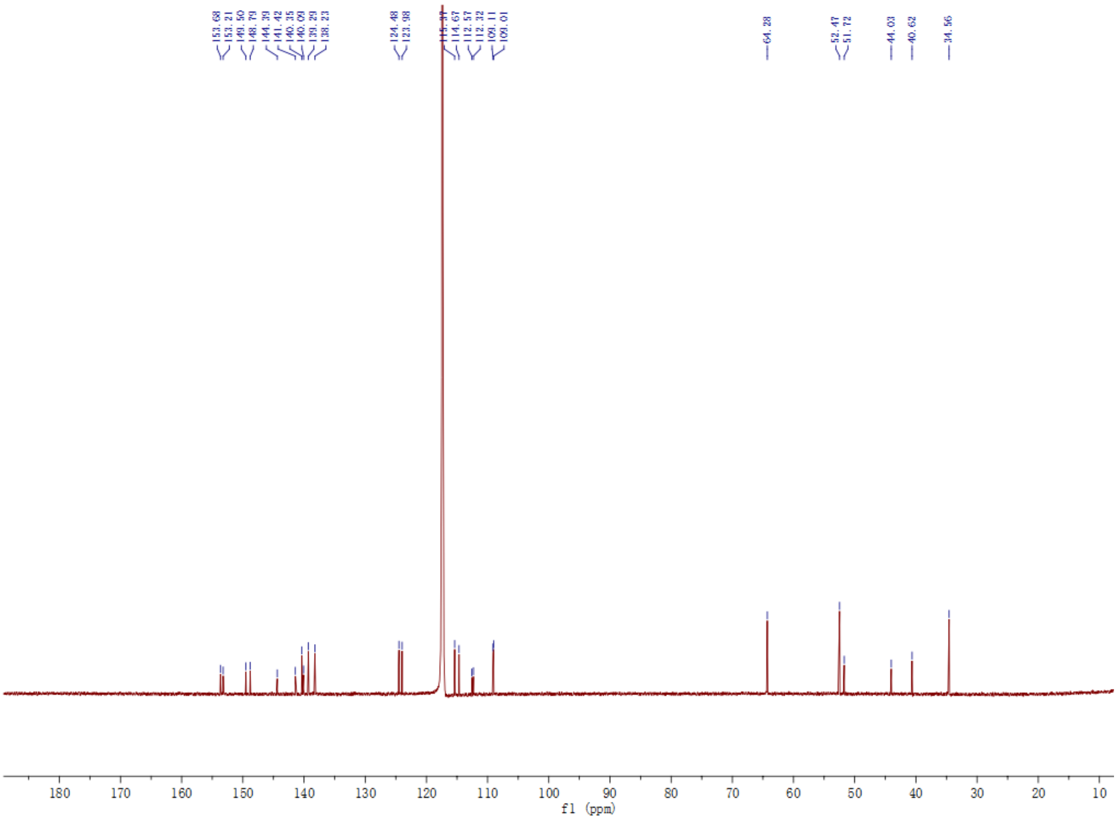

Supplementary Figure 56. ^{13}C NMR spectrum of **7f** in CD_3CN (126 MHz).

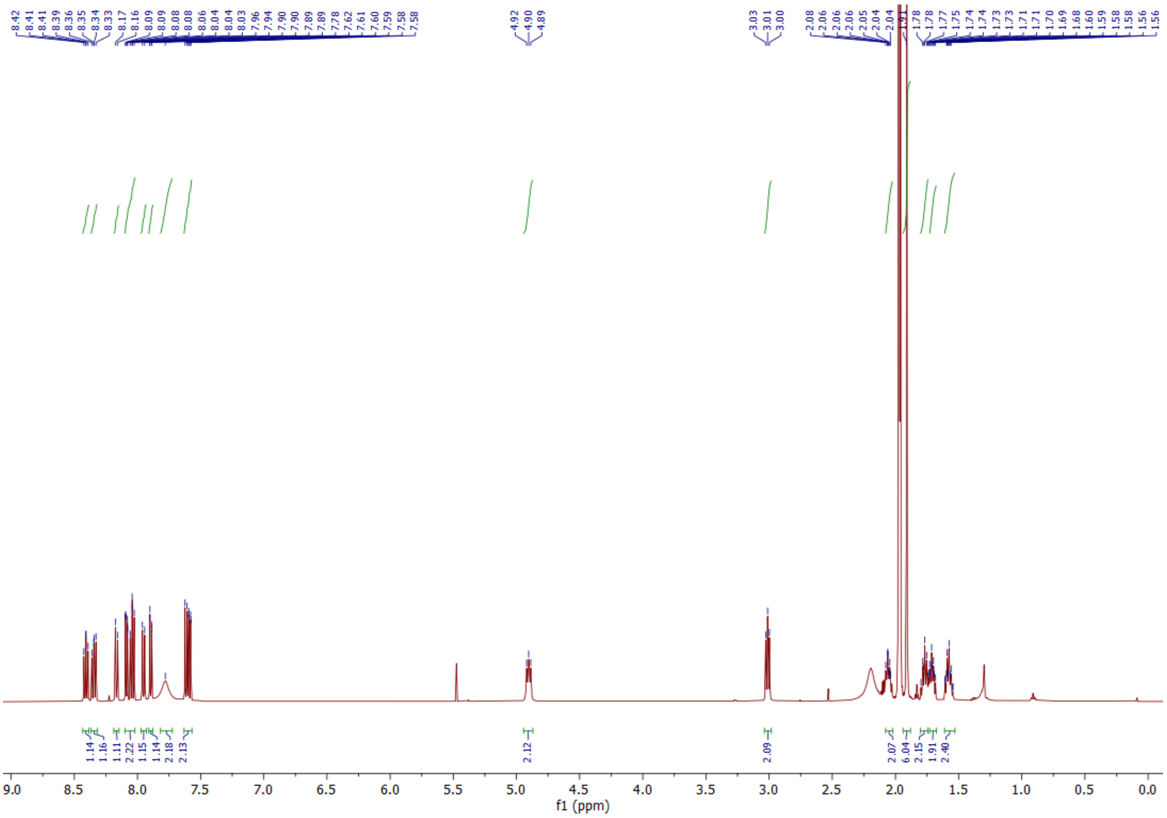

Supplementary Figure 57. ^{1}H NMR spectrum of **7g** in CD_3CN (500 MHz).

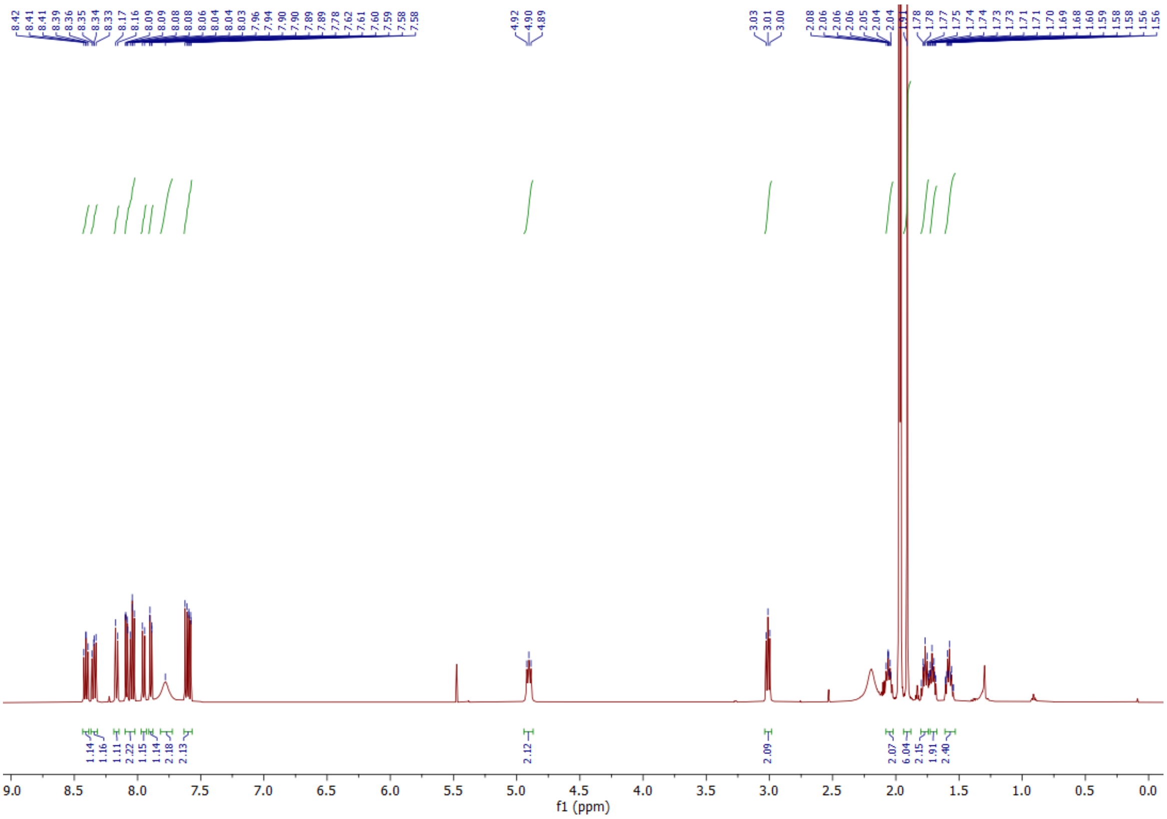

Supplementary Figure 58. ^{13}C NMR spectrum of **7g** in CD_3CN (126 MHz).

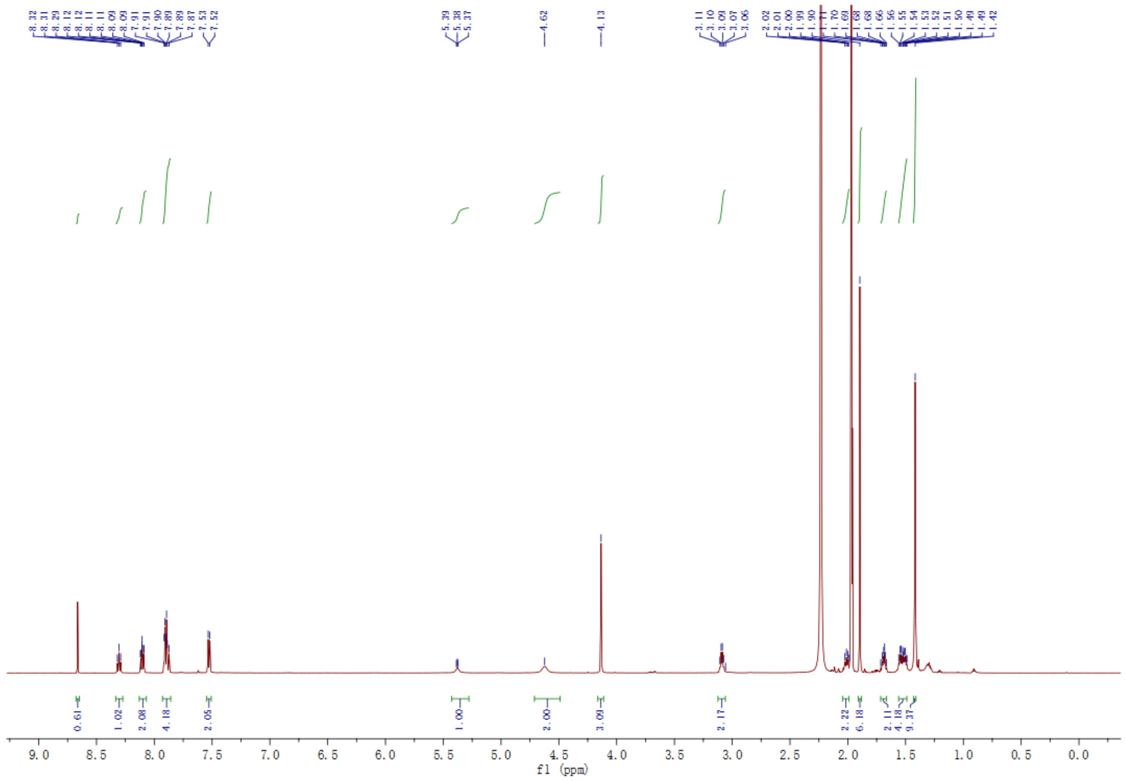

Supplementary Figure 59. ^{1}H NMR spectrum of **8a** in CD_3CN (601 MHz).

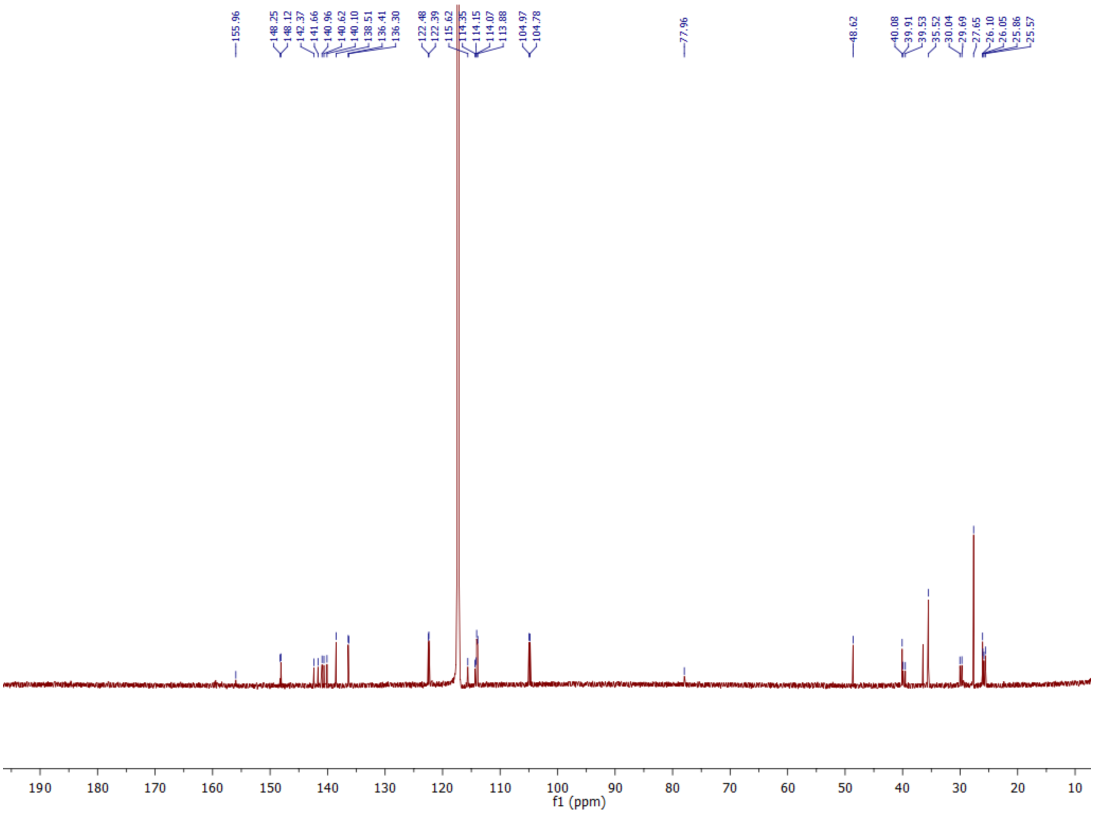

Supplementary Figure 60. ^{13}C NMR spectrum of **8a** in CD_3CN (151 MHz).

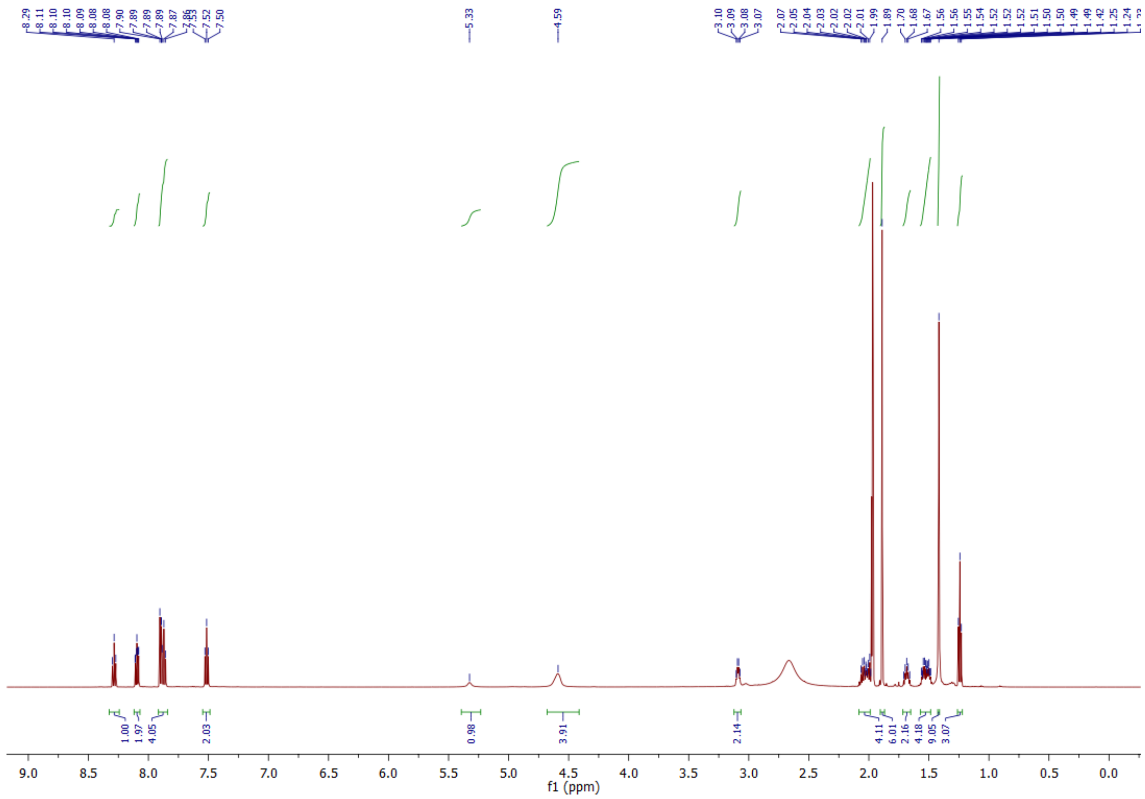

Supplementary Figure 61. ^{1}H NMR spectrum of **8b** in CD_3CN (600 MHz).

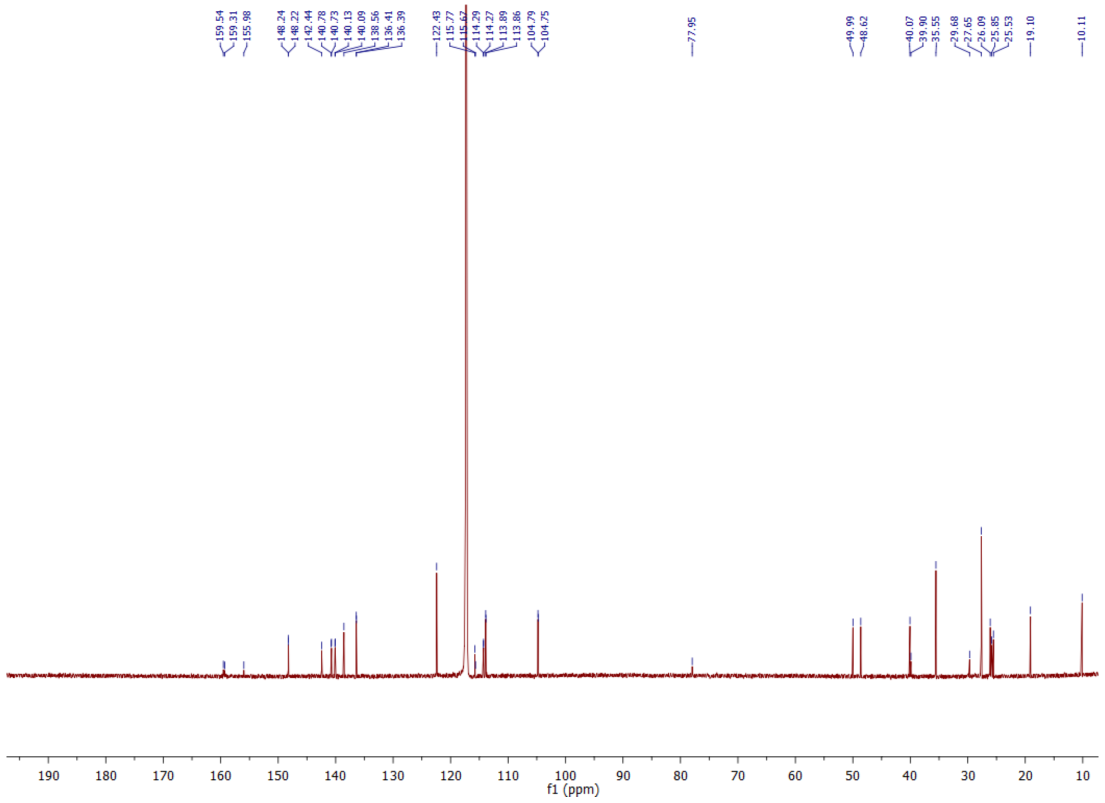

Supplementary Figure 62. ^{13}C NMR spectrum of **8b** in CD_3CN (151 MHz).

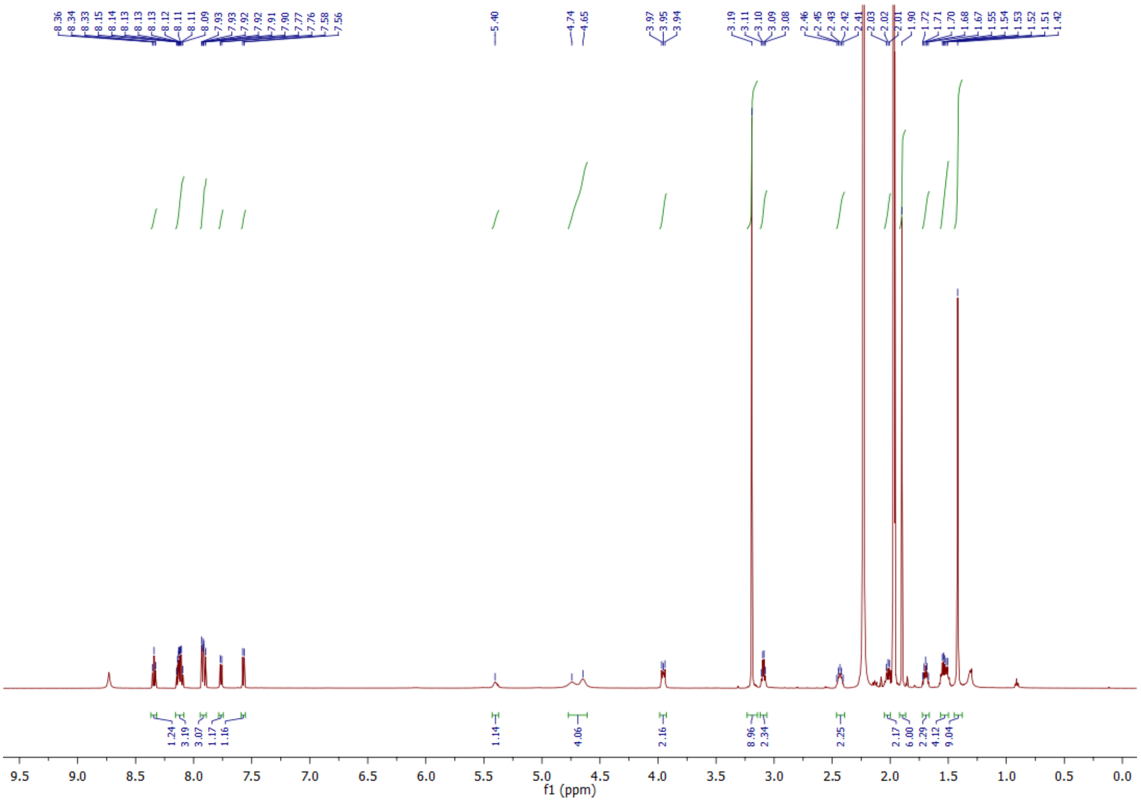

Supplementary Figure 63. ^{1}H NMR spectrum of **8d** in CD_3CN (600 MHz).

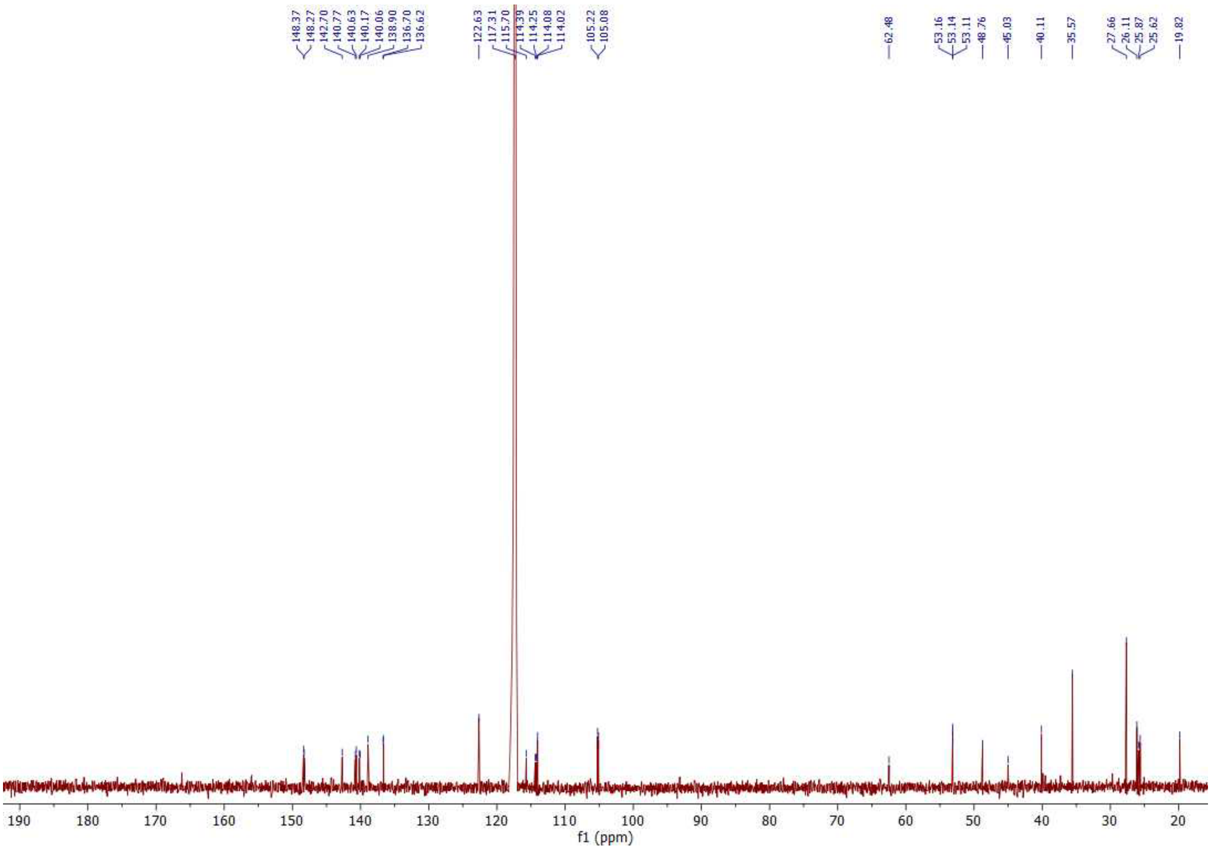

Supplementary Figure 64. ^{13}C NMR spectrum of **8d** in CD_3CN (126 MHz).

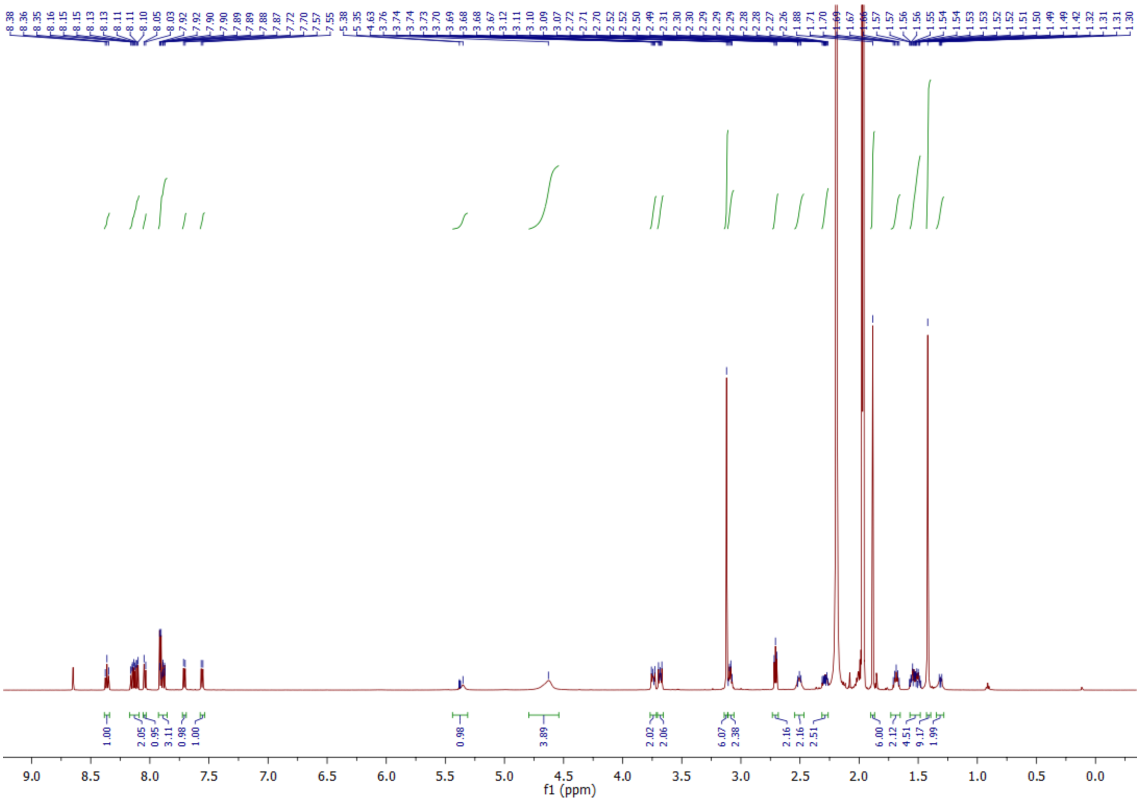

Supplementary Figure 65. ^{1}H NMR spectrum of **8e** in CD_3CN (600 MHz).

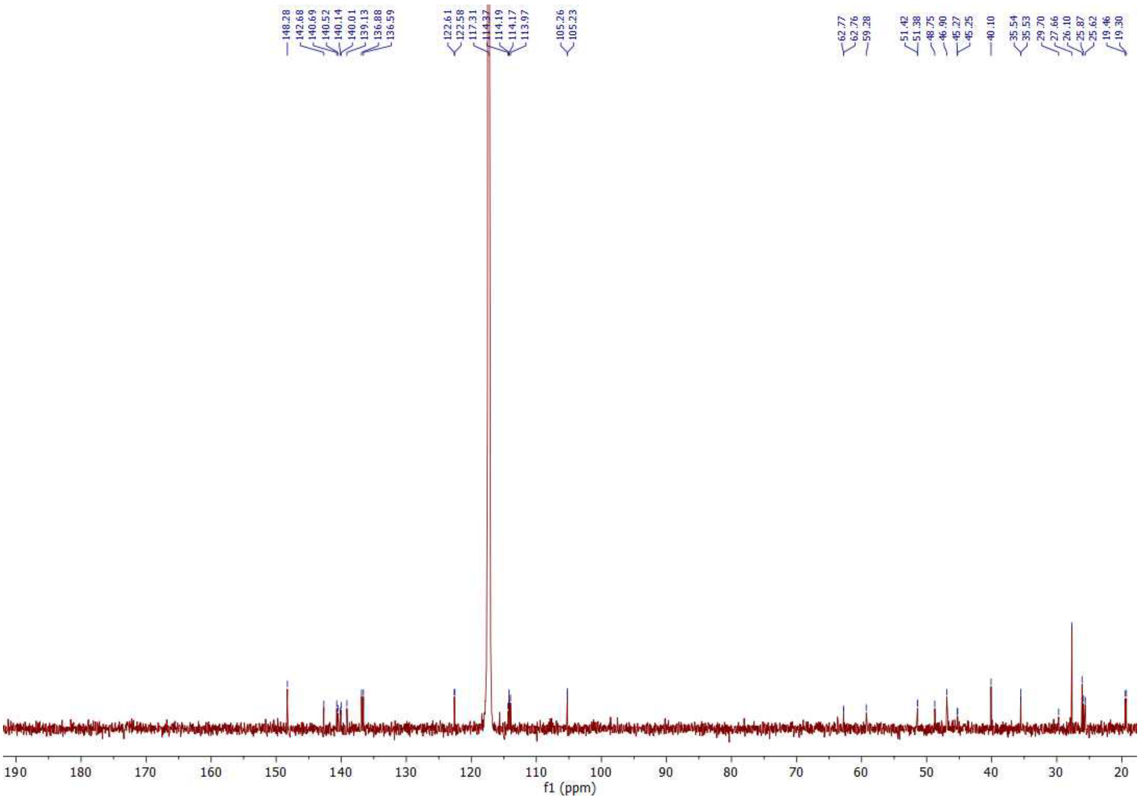

Supplementary Figure 66. ^{13}C NMR spectrum of **8e** in CD_3CN (126 MHz).

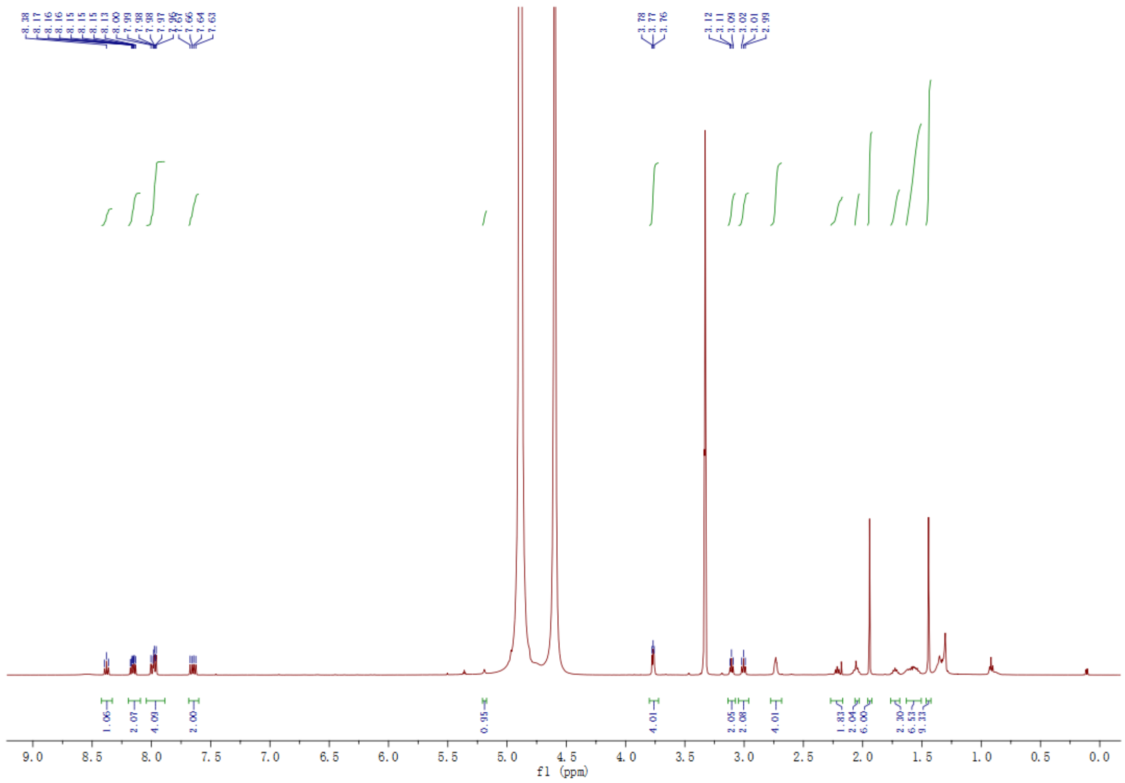

Supplementary Figure 67. ^{1}H NMR spectrum of **8f** in MeOD (500 MHz).

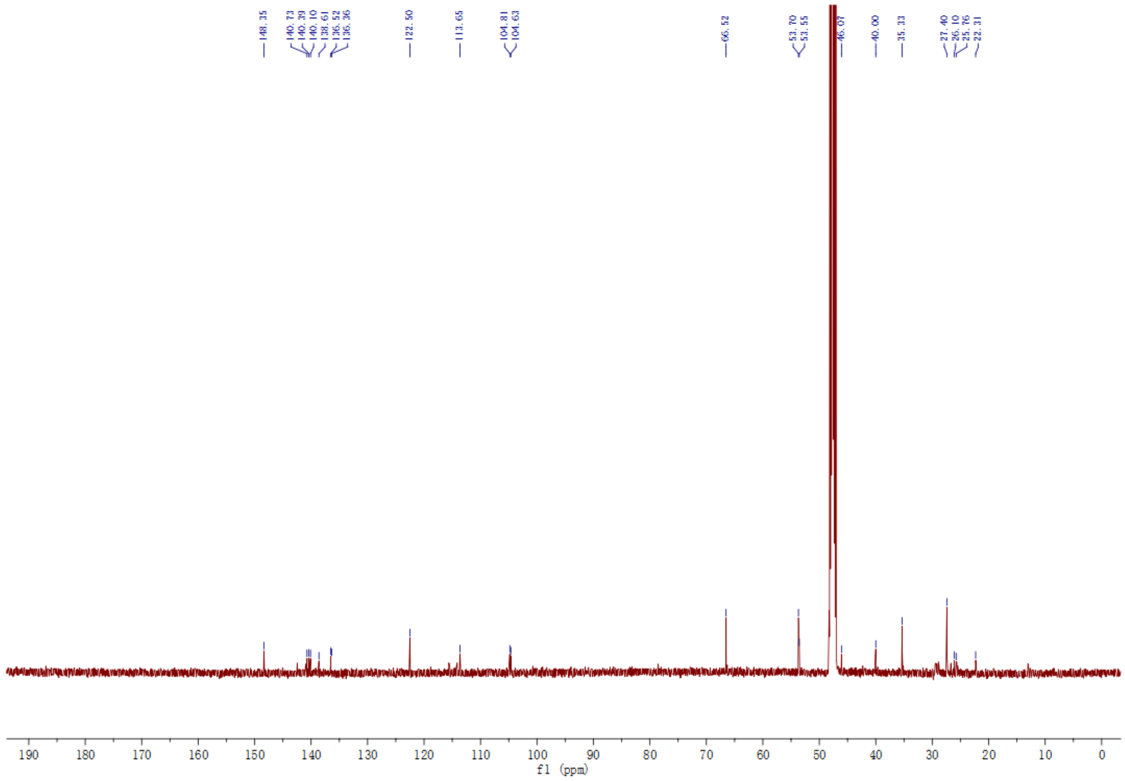

Supplementary Figure 68. ^{13}C NMR spectrum of **8f** in MeOD (126 MHz).
