# Supplementary material for: Lifetime chemical sensor arrays of organic fluorophores for bacterial fingerprinting
Source: Nat Commun. 2026 Apr 28;17:5806. doi: 10.1038/s41467-026-72342-7 (PMC13328534; doi:10.1038/s41467-026-72342-7)
Supplement: Supplementary file 3 — Transparent Peer Review file [file 41467_2026_72342_MOESM3_ESM.pdf]

nature portfolio

Peer Review File

Lifetime chemical sensor arrays of organic fluorophores for bacterial fingerprinting

Corresponding Author: Professor Marc Vendrell

This manuscript has been previously reviewed at another journal. This document only contains information relating to versions considered at Nature Communications.

This file contains all reviewer reports in order by version, followed by all author rebuttals in order by version.

Version 0:

Reviewer comments:

Reviewer #1

(Remarks to the Author)
Dear Authors

I have reviewed this manuscript twice for Nature Chemistry, prior to its transfer to Nature Communications. In these rounds of review the authors have accommodated the comments from me and the other reviews, and optimised the manusript and associated content to a point where no further improvments are relevant.

The manuscript describes the synthesis of several triangulenium dyes and how their long fluorescence lifetime can be used to classify bacteria with antibiotic resistance. I am jealous that I was not the one to do this work, and impressed by the skill and effort the authors have put into the work.

The results, and the usefulness of these long lifetime probes, were replicated with naked dyes and in dye-conjugates. Clearly showing that the methodology is sound and can be expanded to more than just these problematic bacteria.

I see no need for further improvements and suggest that the manuscript is published as is.

Kind regards
Thomas Just Sørensen

Reviewer #4

(Remarks to the Author)
Thanks for the editor's invitation to serve as adjudicative reviewer. In this revised manuscript, Zhou and co-authors presented the first lifetime-based cross-reactive sensor array built from triangulenium (TA) fluorophores. The authors synthesized a diverse library of 20 long-lifetime TA dyes with tunable optical properties and enhanced biocompatibility. They developed a cross-reactive FLIM array that successfully distinguished all seven ESKAPEE bacterial species. Overall, the rational design of the chemical probes is innovative and informative, and the authors have thoroughly optimized fluorophore properties to enhance bacterial membrane permeability.
Here, the reviewers 2 and 3 collectively raised their critical concerns mainly regarding the array specificity, performance in complex real samples, sensitivity, and detection speed. Although some related experiments have been initially carried out by the authors, both reviewers requested more points to be sufficiently resolved. In response, the authors have actually proceeded most of the experiments to prove their concept. Should the authors further provide a few more convincing responses supported by experimental validation to the following minor issues, this work would be suitable for publication in Nature Communications:

1.For Question 3 from Reviewer 2, the validation using spiked human blood samples containing S. aureus or E. coli does demonstrate feasibility in clinical samples. To robustly establish the diagnostic capability of the sensing array, the authors can further consider to evaluate its performance in other clinically relevant body fluids. Given that urinary tract infections

(UTIs) are among the most common bacterial infections globally, testing in urine samples should also be supplemented.

2.In general, the ability to differentiate between structurally similar pathogens in real samples holds significant practical potential. Therefore, it is recommended that the authors could expand their validation in clinical samples beyond the simple model of one Gram-negative and one Gram-positive bacterium or both (answers to Reviewer 2' question 3). Specifically, testing should be extended to groups including two Gram-negative or two Gram-positive bacteria, and further to mixed samples containing three bacterial species (e.g., two Gram-negative and one Gram-positive).

3.For the mechanistic feature of probe lifetime changes, especially for 4b and 8e, the authors have conducted thorough experiments and clearly demonstrated that DNA binding is the crucial mechanism affecting fluorescence lifetime (Supplementary Figure 12). To further support their statement, it is suggested that the authors briefly supply data on fluorescence lifetime changes when TA-peptide probes (PPEP2 and NPEP1) are co-incubated with lipopolysaccharide, peptidoglycans, or similar molecules.

4.For the reviewers' comment on the relatively unclear advantages of this sensing array compared with established techniques (e.g., PCR, MS), it would be great that the authors could perform a blinded test using a mixed multi-bacterial sample. By directly comparing the detection results of this array with those obtained by PCR, the system's rapid readout capability and accuracy could be more directly demonstrated.

Others:
1.As mentioned in this work (Supplementary Figure 23), the proposed detection platform offers a relatively fast assay time (within a few hours) while eliminating the need for extended bacterial culture or highly trained personnel, thus underscoring its practical translational potential. It is recommended that the authors clearly highlight this advantage in the manuscript.

Version 1:

Reviewer comments:

Reviewer #4

(Remarks to the Author)
In this further revised manuscript titled of manuscript "Lifetime chemical sensor arrays of organic fluorophores for bacterial fingerprinting," the authors revised the manuscripts by following the referees' comments. By right, the current manuscript has been greatly improved already and can be thus considered for acceptance in Nat. Comm. with no need further moderation.

Open Access This Peer Review File is licensed under a Creative Commons Attribution 4.0 International License, which permits use, sharing, adaptation, distribution and reproduction in any medium or format, as long as you give appropriate credit to the original author(s) and the source, provide a link to the Creative Commons license, and indicate if changes were made.
In cases where reviewers are anonymous, credit should be given to 'Anonymous Referee' and the source.
The images or other third party material in this Peer Review File are included in the article's Creative Commons license, unless indicated otherwise in a credit line to the material. If material is not included in the article's Creative Commons

license and your intended use is not permitted by statutory regulation or exceeds the permitted use, you will need to obtain permission directly from the copyright holder.
To view a copy of this license, visit https://creativecommons.org/licenses/by/4.0/

Response to Reviewers

Reviewer 4

1. For Question 3 from Reviewer 2, the validation using spiked human blood samples containing S. aureus or E. coli does demonstrate feasibility in clinical samples. To robustly establish the diagnostic capability of the sensing array, the authors can further consider to evaluate its performance in other clinically relevant body fluids. Given that urinary tract infections (UTIs) are among the most common bacterial infections globally, testing in urine samples should also be supplemented.

Answer: We thank the Reviewer for the suggestion. Because we do not have ethics for research with human urine biosamples, we performed experiments in commercially available synthetic urine that was spiked with *P. aeruginosa* (sample U1), *E. coli* (sample U2) or *S. aureus* (sample U3). All samples (U1-U3) were tested with our final 4-member array, which correctly assigned the 3 bacterial species as shown by PCA analysis. These new results are included in the new Supplementary Figure 26.

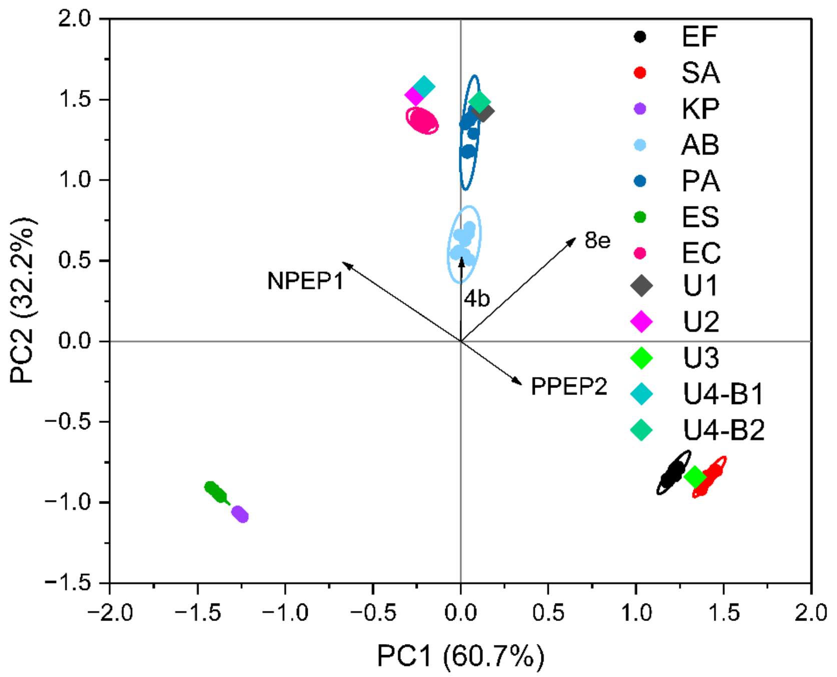

Supplementary Figure 26. PCA plots of the ESKAPEE bacterial panel (dots and solid confidence ellipses) and spiked urine samples (diamonds) using the optimised TA cross-reactive array (**4b**, **8e**, **PPEP1** and **NPEP2**). The training set was built with results of 10 independent images plotted from multiple repeats with 95% confidence intervals.

2. In general, the ability to differentiate between structurally similar pathogens in real samples holds significant practical potential. Therefore, it is recommended that the authors could expand their validation in clinical samples beyond the simple model of one Gram-negative and one Gram-positive bacterium or both (answers to Reviewer 2' question 3). Specifically, testing should be extended to groups including two Gram-negative or two Gram-positive bacteria, and further to mixed samples containing three bacterial species (e.g., two Gram-negative and one Gram-positive).

Answer: We thank the Reviewer for the suggestion. Following the same experimental design than in the previous experiment with urine samples, we spiked a new urine sample (U4) with two gram-negative species (*E. coli* and *P. aeruginosa*) and compared it to the urine samples (U1 for *P. aeruginosa*, U2 for *E. coli*) with individual species. Notably, our array could correctly identify both bacterial species with U4-B1 matching the signature of *E. coli* (same as sample U2) and U4-B2 matching the signature of *P. aeruginosa* (same as sample U1). These new results are included in the new Supplementary Figure 26.

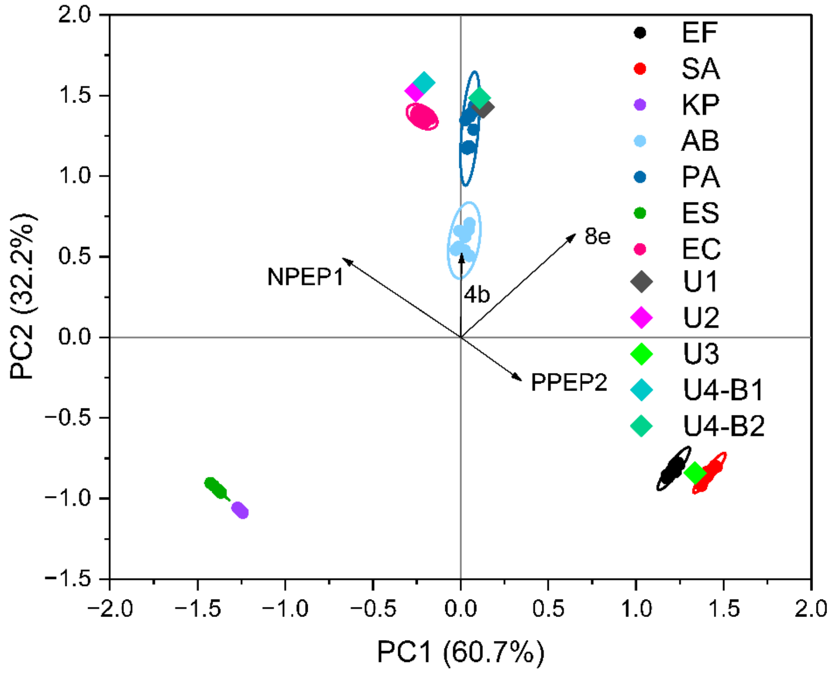

Supplementary Figure 26. PCA plots of the ESKAPEE bacterial panel (dots and solid confidence ellipses) and spiked urine samples (diamonds) using the optimised TA cross-reactive array (**4b**, **8e**, **PPEP1** and **NPEP2**). The training set was built with results of 10 independent images plotted from multiple repeats with 95% confidence intervals.

3. For the mechanistic feature of probe lifetime changes, especially for 4b and 8e, the authors have conducted thorough experiments and clearly demonstrated that DNA binding is the crucial mechanism affecting fluorescence lifetime (Supplementary Figure 12). To further support their statement, it is suggested that the authors briefly supply data on fluorescence lifetime changes when TA-peptide probes (PPEP2 and NPEP1) are co-incubated with lipopolysaccharide, peptidoglycans, or similar molecules.

Answer: We thank the Reviewer for the suggestion. We have measured the fluorescence lifetime of the two peptide probes used in the final array (**PPEP2** and **NPEP1**) in presence of LPS (1 mg mL^{-1}), which is known to be present only in the cell envelope of gram-negative bacteria. We found that **PPEP2** -which only binds gram-positive bacteria- did not show any changes in fluorescence lifetime after incubation with LPS, whereas **NPEP1** -which only binds gram-negative bacteria- showed a substantial increase in fluorescence lifetime after incubation with LPS. These results are in agreement with the fluorescence lifetime imaging

data and confirms the interaction with LPS as a critical factor for the identification of gram-negative bacteria (Figure 1 for Reviewer).

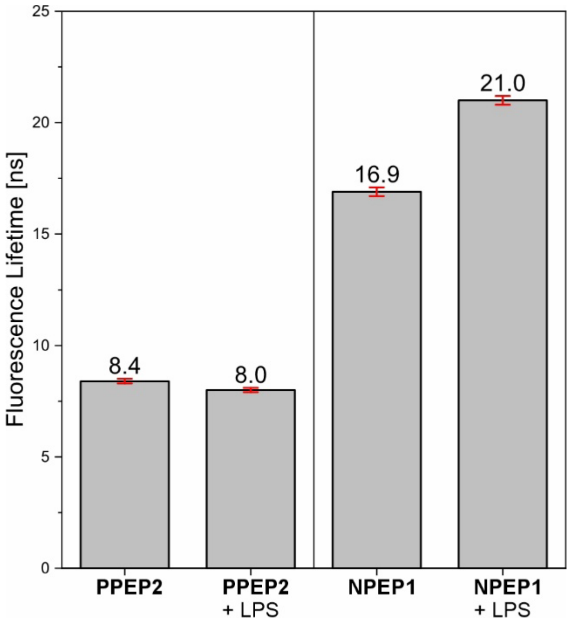

Figure 1 for Reviewer. Fluorescence lifetimes of **PPEP2** and **NPEP1** (both 10 μM) before and after incubation with LPS (1 mg mL^{-1}) in PBS. Values are represented as means and bars represent error of the fit (0.1 ns for **PPEP2**, 0.2 ns for **NPEP1**).

4. For the reviewers' comment on the relatively unclear advantages of this sensing array compared with established techniques (e.g., PCR, MS), it would be great that the authors could perform a blinded test using a mixed multi-bacterial sample. By directly comparing the detection results of this array with those obtained by PCR, the system's rapid readout capability and accuracy could be more directly demonstrated.

Answer: We believe that benchmarking our array against other bacterial identification are out of the scope of this manuscript, where the key development is the design and optimisation of long lifetime-emitting fluorophores and their combination as cross-reactive arrays for distinguishing closely-related cell populations.

5. As mentioned in this work (Supplementary Figure 23), the proposed detection platform offers a relatively fast assay time (within a few hours) while eliminating the need for extended bacterial culture or highly trained personnel, thus underscoring its practical translational potential. It is recommended that the authors clearly highlight this advantage in the manuscript.

Answer: We thank the Reviewer for the comment and highlighted this point in the revised discussion of the manuscript.
